# 6 Circulating miRNAs can be used as Non-invasive Biomarkers for the Detection of Cervical Lesions

**DOI:** 10.7150/jca.51141

**Published:** 2021-06-22

**Authors:** Ruoqi Ning, Silu Meng, Lin Wang, Yao Jia, Fangxu Tang, Haiying Sun, Zhi Zhang, Chong Zhang, Xinran Fan, Bing Xiao, Chunhua Yang, Shuang Li

**Affiliations:** 1 Department of Obstetrics and Gynecology, Tongji Hospital, Tongji Medical College, Huazhong University of Science and Technology, Wuhan, Hubei, 430030, P.R. China.; 2 Department of Pediatrics, Tongji Hospital, Tongji Medical College, Huazhong University of Science and Technology, Wuhan, Hubei, 430030, P.R. China.; 3 National Engineering Research Center for Beijing Biochip Technology, Changping District, Beijing, 102206, P.R. China.; 4 CapitalBio Corporation, Changping District, Beijing, 102206, P.R. China.

**Keywords:** Circulating miRNA, Biomarker, Non-invasive, Cervical cancer, Cervical intraepithelial neoplasia

## Abstract

**Background:** Cervical cancer is the most common malignant tumor in the female reproductive system, while the efficacy of routine screening strategy is unsatisfied. New molecular tests need to be developed. miRNAs participate in many pathologic processes, and circulating miRNAs are promising non-invasive biomarkers in tumors.

**Objectives:** This study aimed to identify the circulating miRNAs associated with both cervical cancer and cervical intraepithelial neoplasia (CIN), and establish a non-invasive classifier for cervical lesions using circulating miRNAs.

**Methods:** This study consisted of 5 steps: miRNAs screening, miRNAs validation, classifier establishment, independent validation and *in silico* analyses. Three cohorts were included in our study: In screening stage, 24 samples including 14 cases and 10 controls were retrieved; In validation stage, 380 samples including 200 cases and 180 controls were recruited; In independent validation stage, 47 samples comprising 26 cases and 21 controls were included. miRNAs were quantified by RT-qPCR. A classifier was built with random forest algorithm using validation samples and selected miRNAs, which were then validated in an independent cohort. To explore the function of selected miRNAs, *in silico* analyses were performed. Target genes of selected miRNAs were predicted by the overlap of three online tools. Enrichment analyses were executed with predicted target genes. Differential analysis of target genes was carried out with open access expression assay datasets of cervical tissues.

**Results:** 6 miRNAs (hsa-miR-26b-5p, hsa-miR-146b-5p, hsa-miR-191-5p, hsa-miR-484, hsa-miR-574-3p, hsa-miR-625-3p) were screened out from 754 miRNAs. They were associated with cervical lesions and were selected to establish a classifier. The accuracy of the classifier were 0.7218 (0.7117, 0.7319) in validation samples, which was 0.7021 in the independent cohort. 958 target genes were predicted and enriched in 23 pathways (MAPK, human papillomavirus infection and Wnt signaling pathway, etc.). 55 genes were identified as the most likely target genes by differential analysis.

**Conclusion:** The 6 circulating miRNAs were related to cervical lesions and could serve as non-invasive biomarkers.

## Introduction

Cervical cancer is the fourth leading cancer in women worldwide, and the estimated number of new cases and deaths in 2018 were 569,847 and 311,365 respectively according to GLOBOCAN 2018 1. Most of the time, cervical cancer starts from a precancerous lesion called cervical intraepithelial neoplasia (CIN) and it usually takes years even decades to develop invasive cancer. CIN is curable and the prognosis of the early stage of cervical cancer is good, while that of the late stage is poor 2. Hence early diagnosis is a highly efficient way to improve the survival rate of cervical cancer. The early detection strategy recommended by screening guidelines is cytology with or without human papillomavirus (HPV) testing depending on age 3, which are invasive and the efficacy is insufficient. For the detection of CINII or severer, the sensitivity of cytology alone is low (53.2%), the specificity of HPV testing alone is moderate (62.7%), and the sensitivity of co-testing ranges from 51.4% to 67.5% 4. Therefore, new non-invasive biomarkers need to be developed to improve the detection efficacy of cervical lesions.

microRNAs (miRNAs) are small regulatory RNAs (about 22nt) that participate in physiologic and pathologic processes by inhibiting or degrading mRNAs of target genes 5. Many studies have shown that differentially expressed miRNAs in tissue are detected in a variety of cancers, which could also be found in extracellular liquid or blood 6. Circulating miRNAs were confirmed stable in both plasma and serum, and expression levels were reproducible and consistent among individuals, which indicated its feasibility as non-invasive biomarkers 7. Promising circulating miRNA classifiers had been established in a range of tumors, such as hepatocellular carcinoma 8. Some studies had depicted the circulating miRNA expression profiles in cervical cancer and CIN, but the results of different studies were inconsistent 9.

Herein this study aimed to identify the plasma miRNAs associated with both cervical cancer and CIN, and establish a non-invasive classifier for cervical lesions using plasma miRNAs.

## Methods

### Study design and participants

Our study consisted of 5 steps: miRNAs screening, miRNAs validation, classifier establishment, independent validation and in silico analyses (Figure S1). The participants, except those of *in silico* analyses, were recruited from Tongji Hospital, Tongji Medical College, Huazhong University of Science and Technology between 2014 and 2020. Inclusion criteria of cases: (1) Adult women aged 20 to 80. (2) Patients diagnosed as CINII, CINIII or cervical squamous cell carcinoma according to biopsy and pathological reports. (3) No history of other malignant tumors or family history of malignant tumors. (4) No therapies including radiotherapy, chemotherapy or surgery have been given. Inclusion criteria of controls: women aged 20 to 80 without cervical lesions nor malignant diseases. Exclusion criteria: pregnant or lactating women. This study was approved by the Ethics Committee of Tongji Hospital, Tongji Medical College, Huazhong University of Science and Technology. All participants joined this study with written informed consents.

### Sample preparation and detection

Venous blood was collected in 4 mL ethylene diamine tetraacetic acid vacuum blood collection tube and centrifuged at 1600 g (4 °C) for 10 min. The supernatant plasma was collected and centrifuged at 12000 g (4 °C) for 10 min. The second supernatant plasma was collected and stored at -80 °C until detection. Total cell-free RNA was purified from plasma using the miRNeasy Serum/Plasma Kit (Cat. no. 217184, QIAGEN) following the handbook. Concentration detection and quality checking of RNA was performed with Thermo NanoDrop^TM^ Lite spectrophotometer and Agilent 2100 respectively. Relative quantitation of miRNA was determined by quantitative reverse transcription polymerase chain reaction (RT-qPCR) using TaqMan® MicroRNA Assays Kits (Applied Biosystems) and 7900HT Fast Real-Time PCR system (Applied Biosystems) according to the manufacturer's instruction.

### Data analysis

The comparison of miRNA levels between two groups in the screening stage was conducted with DataAssist™ ver.3.01 software using the 2^-ΔΔCt^ method, and hsa-miR-16 was used as the internal reference. miRNAs satisfied the following criteria were selected: Detection rate (Proportion of cycle threshold [Ct] ≤ 35 in total samples) ≥ 0.9, fold change (FC) > 2.2 and *P* < 0.03 (Figure S1). In validation stage, the mean Ct of ath-miR-159a, hsa-miR-1228 and hsa-miR-16 was used as the internal reference. The miRNA levels between case and control groups in the validation and independent stages were compared using the 2^-ΔΔCt^ method, and the ΔCt was also compared between groups with the Mann-Whitney U test (SPSS statistics, ver. 25.0, IBM).

Data of ΔCt in the validation stage was used to establish a classifier. 70% of samples were randomly divided into training set and 30% to sub-validation set. A random forest model was established with the training dataset and *randomForest* package of R (Arguments: ntree = 100, mtry = 3, others were default). The model was applied to the sub-validation dataset to evaluate the efficacy of the classifier. 50 times of 7:3 random sampling was conducted and the average efficacy indicators (accuracy, sensitivity, specificity and AUC) were calculated. The classifier was applied to the independent cohort and the efficacy indicators were also calculated. All figures were generated by Prism (Ver.8, GraphPad) and R (Ver. 3.6.0).

### Target gene prediction and enrichment analyses

Target genes of the selected miRNAs were predicted using three online platforms (Diana microT-CDS ver.5.0, miRDB ver.6.0, TargetScanHuman ver.7.2), and only overlapped genes of the three tools were selected as predicted target genes for each miRNA. To explore the related functions and pathways of the predicted target genes, Gene Ontology (GO) and Kyoto Encyclopedia of Genes and Genomes (KEGG) pathway enrichment were analyzed with the *clusterProfiler* package. BH (Benjamini-Hochberg) method was used for multiple test correction, and the thresholds of both GO and KEGG pathway enrichment analyses were q-value < 0.1 and adjusted p-value < 0.2.

### Target gene validation in tissue expression array datasets

To further identified the most likely target genes, Gene Expression Omnibus (GEO) database was exhaustively searched for tissue expression profiling arrays. Datasets inclusion criteria: Both normal and high grade cervical lesions (high grade squamous intraepithelial lesions [HSIL] or cervical cancer) samples were contained, and the sample size of normal was no less than 10. *Limma* package was used to identify the differentially expressed genes (DEGs). Threshold for DEGs: |Log FC| ≥ 1 and adjusted p-value < 0.05. Consistently downregulated target genes from all included datasets (only down or not different results were observed) were presumed as the most likely target genes.

## Results

**Figure 1:**
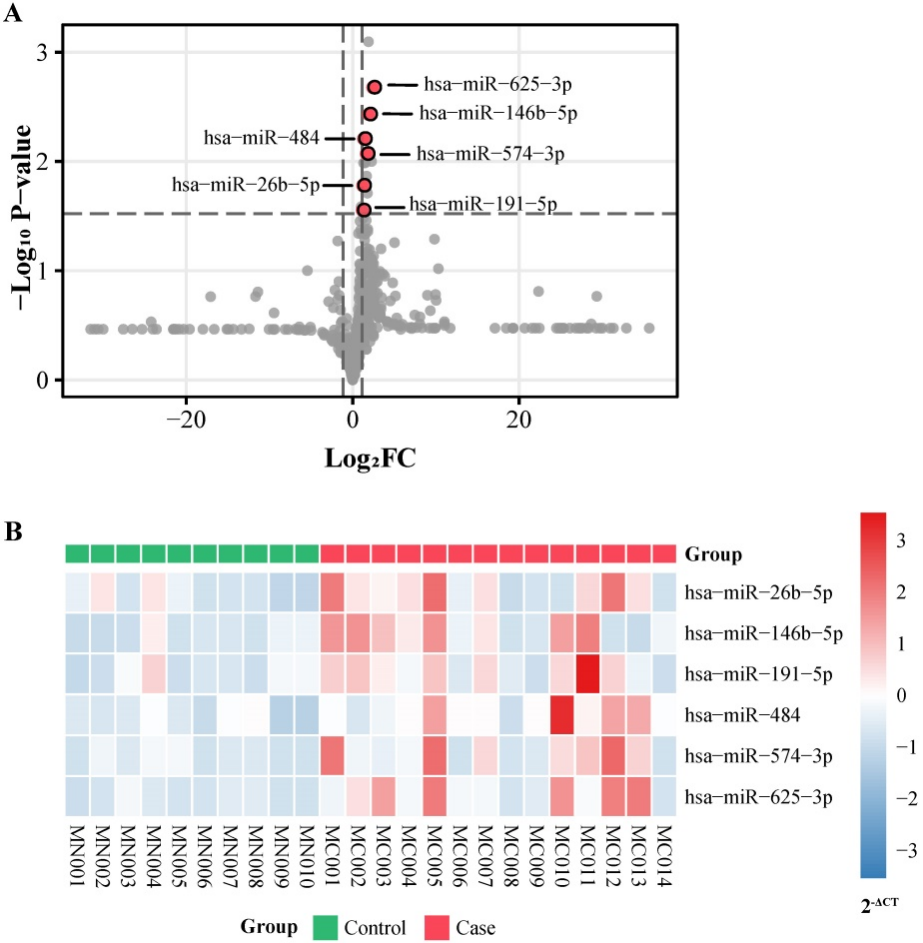
miRNA screening. **A,** p-value and log_2_ FC of all miRNAs detected in screening stage. FC: fold change. **B,** Expression level (2^-ΔCt^) of 6 selected miRNAs in screening samples.

451 participants including 240 cases and 211 controls were recruited in total (Table 1). To screen out the differential miRNAs, 754 human miRNAs were detected among 13 cervical cancer patients, 1 CINIII patient and 10 normal controls in the screening stage (Figure S1). The median ages of cases and controls in the screening set were 47.0 and 49.5 respectively. 6 candidate miRNAs (hsa-miR-26b-5p, hsa-miR-146b-5p, hsa-miR-191-5p, hsa-miR-484, hsa-miR-574-3p, hsa-miR-625-3p) reached the threshold and were selected for validation (Figure 1A, Table S1). All of them expressed higher in the case group (Figure 1B).

**Figure 2:**
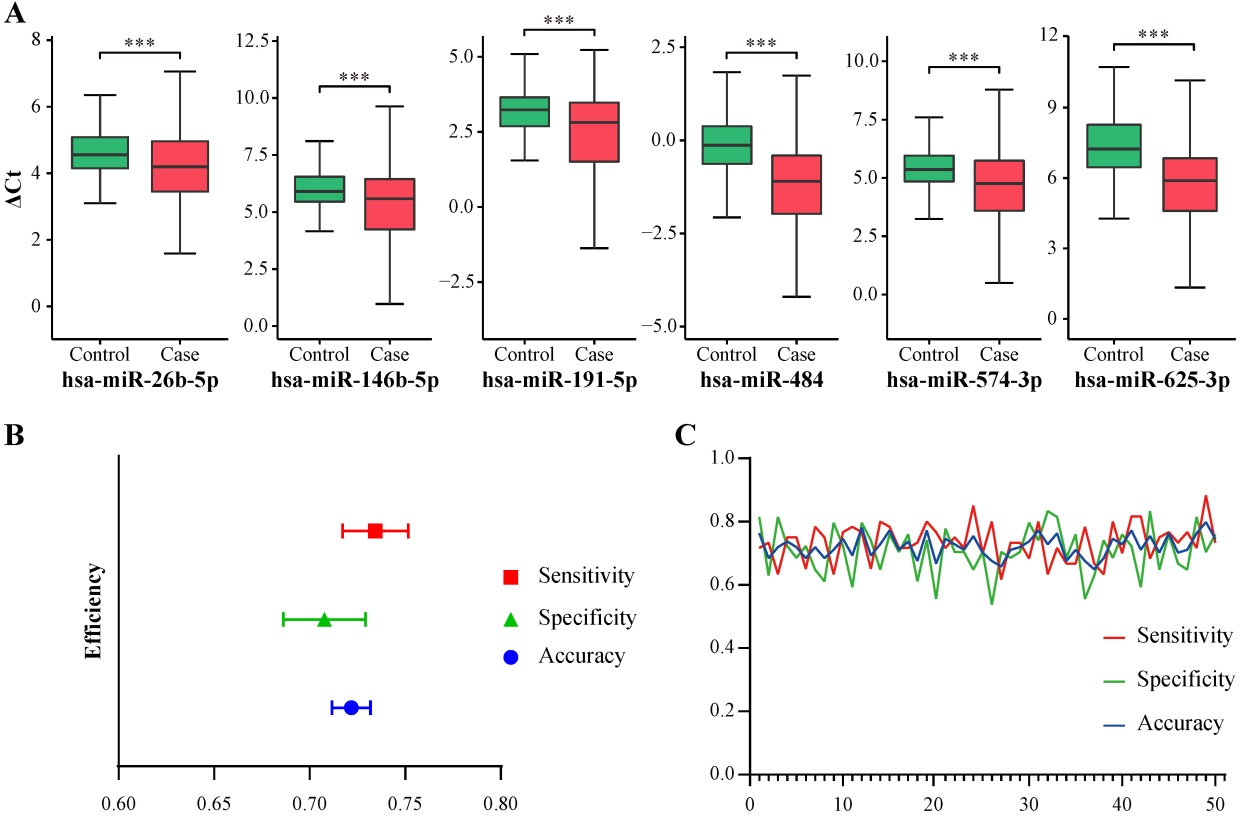
miRNA validation and classifier evaluation. **A,** ΔCt of 6 selected miRNAs in validation samples. ***, *P* < 0.001. **B,** Means and 95% confidence intervals of accuracy, sensitivity and specificity of the classifier generated from 50 random sampling datasets. **C,** Line chart of accuracy, sensitivity and specificity of the classifier generated from 50 random sampling datasets.

The candidate miRNAs were then validated in a validation cohort comprising 180 controls, 100 CIN and 100 cervical cancer patients. The median age of the case group was 44.5 years old, and that of the control group was 42.0 (Table 1). The expression levels (2^-ΔCt^) of the six miRNAs were all significantly higher in the case group (i.e. *P* < 0.001, Table S2). ΔCt of each miRNA were also compared between case and control groups, which represented significant differences (i.e. *P* < 0.001, Figure 2A and Table S2). Then, the six miRNAs were selected to establish the classifier.

Because 2^-ΔCt^ in validation dataset was small (i.e. large amount of values were less than 0.1) and containing extreme values, which may cause loss of information during model establishment, ΔCt was used to build the classifier in our study. There are some studies using ΔCt instead of 2-ΔCt to establish classifier. Liu et al. established a circulating microRNA signature as noninvasive diagnostic and prognostic classifier for nonalcoholic steatohepatitis using -ΔCt 10. Wang et al. built a biomarker model using the ΔCt values of 13 miRNAs to differentiate ADHD patients from control subjects 11. Although ΔCt was not linearly correlated with the relative concentration of miRNA, it was still negatively correlated to the relative concentration and could help to discriminate cases from controls (Figure 2A and Table S2). Random forest algorithm was applied to build the classifier using the total samples in the validation dataset. To train and evaluate the model objectively, 50 times of 7:3 random sampling were conducted and 50 pairs of training (70%) and sub-validation (30%) datasets were retrieved. Models established from training samples were evaluated in corresponding sub-validation samples. 50 sets of evaluation indicators were relatively stable (Figure 2B-C). The means and 95% confidence intervals of area under curve (AUC), accuracy, sensitivity and specificity were 0.7211 (0.7108, 0.7313), 0.7218 (0.7117, 0.7319), 0.7343 (0.7172, 0.7515) and 0.7078 (0.6862, 0.7293) respectively (Table S3).

The selected miRNAs and classifier were further validated in an independent cohort. 26 cases and 21 controls were included (Table 1). Both 2^-ΔCt^ and ΔCt of 6 miRNAs were significantly different (*P* ≤ 0.05) between case and control group (Figure S2A, Table S4). The accuracy and AUC of the classifier were 0.7021 and 0.6896 respectively (Figure S2B, Table S3). Therefore the 6 miRNAs were associated with cervical cancer and CIN. The efficacy of the classifier was moderate.

**Figure 3:**
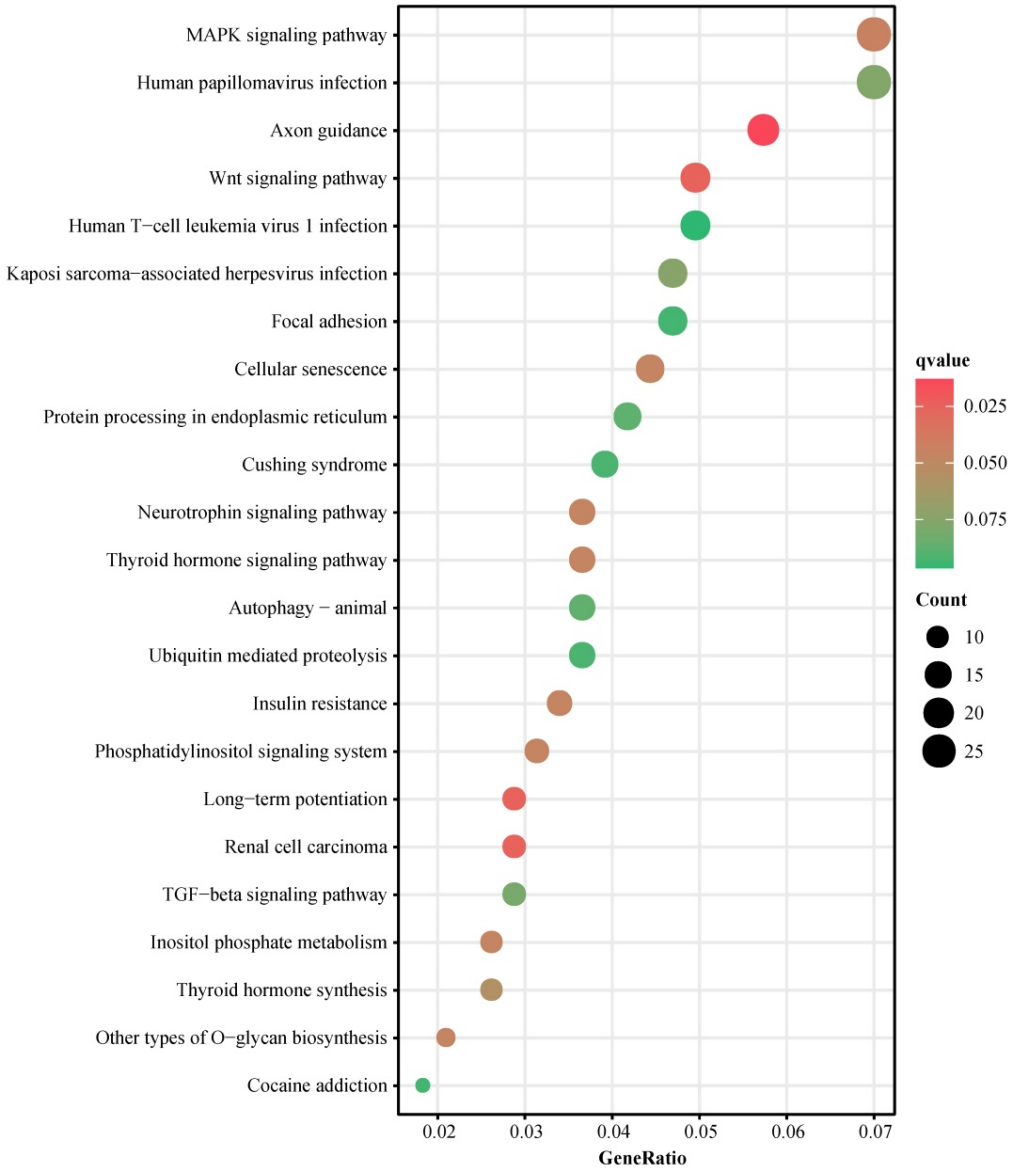
KEGG pathway enrichment analysis. KEGG: Kyoto Encyclopedia of Genes and Genomes.

To explore the functions of the 6 miRNAs in cervical lesion development, *in silico* analyses were performed. Target genes of miRNAs were predicted by the overlap of three online tools. In total, 958 genes were predicted as target genes for 6 miRNAs (Figure S3, Table S5). Number of predicted target genes of single miRNA ranged from 3 (hsa-miR-574-3p) to 558 (hsa-miR-26b-5p). KEGG pathway and GO Enrichment analyses were implemented to investigate the potential mechanisms involved with the 6 miRNAs using the total 958 predicted genes. 23 pathways were enriched including the MAPK pathway, human papillomavirus infection pathway and Wnt signaling pathway (Figure 3, Table S6). 174 biological processes, 17 cellular components and 15 molecular functions were enriched (Figure S4, Table S6).

miRNAs usually function as gene suppressors by inhibiting or degrading the mRNA of target genes. Tumor/lesion cells could excrete aberrant miRNAs to blood. Therefore, dysregulated miRNAs identified from the plasma of patients may have similar inhibitory functions in tumor/lesion cells. The six selected miRNAs were expressed higher in patients, hence the downregulated genes in cervical lesion tissue may be the most likely target genes. GEO database was searched for mRNA expression assay data of cervical tissues. Finally, 5 datasets (GSE63514, GSE9750, GSE7803, GSE52903, and GSE27678) were included in this study (Table S7). 952 of the 958 predicted target genes were covered by at least one of the selected GEO datasets and the mRNA expression profiles were provided in Table S8. 55 genes consistently downregulated in cervical lesions among selected datasets were deduced to be the most likely target genes (Table S9).

## Discussion

6 miRNAs (hsa-miR-26b-5p, hsa-miR-146b-5p, hsa-miR-191-5p, hsa-miR-484, hsa-miR-574-3p, hsa-miR-625-3p) associating with cervical cancer and CIN were identified. Then a classifier constituted with these miRNAs was established, and the efficacy was moderate and stable. These miRNAs may participate in the development of cervical lesions through regulating genes in the cell growth processes, MAPK pathway, human papillomavirus infection pathway, and Wnt signaling pathway, etc.

Recent studies have depicted some dysregulated circulating miRNAs in cervical cancer patients, but most of the results were inconsistent 9. The expressions of miRNAs reported repeatedly by preceding studies, such as hsa-miR-20a-5p, hsa-miR-21-5p, and hsa-miR-486-5p were not differentially expressed in our screening dataset (Table S1). Among 6 miRNAs of our study, only hsa-miR-26b-5p and hsa-miR-191-5p were reported to be candidate biomarkers in the cervical mucus for early detection of CIN 12, while the other four (hsa-miR-146b-5p, hsa-miR-484, hsa-miR-574-3p, hsa-miR-625-3p) were first reported to be associated with cervical lesions.

A multi-stage study design is usually a better way to identify biomarkers 8. In our study, a three-stage investigation of markers including screening, validation and independent validation were conducted. Most of the studies detected limited candidate miRNAs without screening stage 9, which lacked theoretical basis. 754 miRNAs were detected in the screening stage of our study, which was more extensive and objective. Zhang et al. proposed a panel of serum miRNAs as biomarkers for cervical cancer and CIN by screening, training and validation phases. 4 miRNAs (miR-16-2*, miR-195, miR-2861, miR-497) were screened out from 444 miRNAs. The differential diagnosis model was based on logistic regression and constructed with combined samples of training and validation phases. The AUC of the panel was 0.849 (cervical cancer versus healthy controls) and 0.734 (CIN versus healthy controls) 13. Ma et al. conducted a 4-step study and identified a panel of 4 miRNAs (from 179 miRNAs) for diagnosis of cervical cancer. The AUC of the panel in testing and external validation phases were 0.774 and 0.786 respectively 14. There are similarities in our research designs, but the final selected miRNAs of our study were totally different from those of Zhang et al. and Ma et al. 13, 14. A wider spectrum of miRNAs (754) were covered in our study while those of the above studies were much smaller. Although the AUC of our classifier was lower than both of theirs, our evaluation method was more objective than the AUC of logistic regression constructed with total samples in the study of Zhang et al., which may be over evaluated 13. The total small sample size of Ma et al. was much smaller than our study 14.

miRNAs are regulatory molecules that play important roles in HPV infection, cervical cancer initiation, development and progression 9. Target genes of differentially expressed cellular miRNAs in cervical lesions were enriched to pathways involved in cancer development and progression. Such as cell proliferation, cell cycle, angiogenesis, apoptosis, cell migration and invasion 15. The miRNA expression in circulation may originate from blood, tumor and other organ/tissue-specific cells 9. Therefore, the aberrant circulating miRNAs in cervical lesion patients may function the same regulatory effect in tissues. In our study, the target genes of 6 circulating miRNAs were predicted, and the KEGG pathway and GO enrichment analyses were implemented. The predicted target genes enriched in many canonical cervical cancer-related pathways, such as MAPK signaling pathway, human papillomavirus infection pathway, Wnt signaling pathway, etc. GO enrichment analyses revealed that the predicted target genes were involved in cell growth and cell cycle processes which were closely related to tumor development. These results, in turn, supported that the 6 miRNAs may be involved in the cervical lesions development.

hsa-miR-26b-5p locates at nucleus and extracellular space. It takes part in microRNAs in cancer pathway and Parkinson disease pathway. hsa-miR-26b-5p was reported playing important roles in many kinds of tumors, such as esophageal squamous cancer 16 and oral squamous cell carcinoma 17. hsa-miR-26b-5p and hsa-miR-191-5p were reported associated with CIN 12. Among the 41 most likely target genes of hsa-miR-26b-5p, *WNT5A* 18, *EPHA2* 19, *PMI* 20 and *CCND2* 21 were reported functioning as oncogenes in cervical cancer, while *HPGD* 22, *ESR1* 23, *KLF4* 24 function as tumor suppressors. hsa-miR-191-5p locates at nucleus, cytoskeleton and extracellular space. It was reported taking part in the development of many diseases including hepatocellular carcinoma 25 and gastric cancer 26. Among the two most likely target genes of hsa-miR-191-5p, *SATB1* was considered as an oncogenic gene inducing cell cycle arrest with the assistance of p16 and the RB/E2F pathway 27. hsa-miR-191-5p may involve in cervical lesion development by targeting *SATB1*. hsa-miR-146b-5p, hsa-miR-484, hsa-miR-574-3p and hsa-miR-625-3p have not been reported to involve with cervical lesions before. hsa-miR-146b-5p is widely distributed inside and outside the cell and takes part in cell differentiation pathway. It was reported associated with papillary thyroid cancer 28. Among the two the most likely target genes of hsa-miR-146b-5p, *NOVA1* could be downregulated by E6 and E7 genes after HPV infection 29. hsa-miR-146b-5p may partake in the process of HPV infection by regulating *NOVA1*. hsa-miR-484 is widely distributed inside and outside the cell mainly in nucleus and extracellular space. It takes part in cell cycle pathway and play roles in development of many kinds of tumors, such as breast cancer 30 and colorectal cancer 31. Among the 6 most likely target genes, hsa-miR-484 may involve in the development of cervical lesion by targeting *SORBS2*, a tumor suppressor in cervical cancer 32. hsa-miR-625-3p is widely distributed inside or outside the cell and participates in the microRNAs in cancer pathway. Circulating hsa-miR-625-3p was reported as potential biomarkers of malignant pleural mesothelioma 33 and lung tumors 34. Among the 4 most likely target genes of hsa-miR-625-3p, *TGFBR1* was reported correlating with the malignant transformation of the uterine cervix 35, which may explain the association between hsa-miR-625-3p and cervical lesions. hsa-miR-574-3p is widely distributed inside or outside the cell. It was reported as a potential biomarker of epithelial ovarian cancer 36 and a tumor suppressor in prostate cancer 37. Although no target gene of hsa-miR-574-3p was selected from GEO tissue expression array datasets, it might still play roles in pathological processes of cervical lesions indirectly, which needs more researches.

There were some limitations in our study. Cytology and HPV testing were not included in our data analyses because the data was unavailable in many controls. Only one center was included and the sample size of independent validation cohort was small. Multi-center studies with larger sample size should be conducted in the future. It is worth further exploring the mechanisms of the association between the six miRNAs and cervical lesions in subsequent researches. In summary, our study proposed new circulating miRNAs associated with cervical lesions and established a classifier using the 6 circulating miRNAs which may serve as non-invasive biomarkers and improve the efficacy of cervical lesions screening.

## Supplementary Material

Supplementary figures and tables.

## Figures and Tables

**Table 1:** Demographic and clinical characteristics of the participants

Characteristics	Screening (n=24)		Validation (n=380)		Independent validation (n=47)	
	Case (n=14)	Control (n=10)	Case (n=200)	Control (n=180)	Case (n=26)	Control (n=21)
Age^a^ (years)	47.0 (36.0-57.0)	49.5 (37.0-69.0)	44.5 (22.0-71.0)	42.0 (25.0-70.0)	37.0 (31.0-55.0)	48.0 (26.0-69.0)
**Stages^b^**						
Pre-cancerous lesions	1		100		21	
I	8		58		0	
II	5		38		5	
III	0		4		0	
IV	0		0		0	
**Differentiation**						
Well differentiated	1		6		0	
Moderately differentiated	6		36		1	
Poorly differentiated	6		28		3	
NA	1		130		22	
**Lymph node metastasis**						
No	12		162		26	
Yes	2		13		0	
NA	0		25		0	
**Tumor size**						
< 4 cm	6		149		21	
≥ 4 cm	6		25		3	
NA	2		26		2	

^a^Data are medians and ranges. ^b^According to the International Federation of Gynecology and Obstetrics staging of carcinoma of the cervix uteri (2018). Pre-cancerous lesions include CINII and CINIII. Abbreviations: NA: not available.

## Abbreviations

CIN: cervical intraepithelial neoplasia
HPV: human papillomavirus
miRNAs: microRNAs
RT-qPCR: quantitative reverse transcription polymerase chain reaction
AUC: area under curve
FC: fold change
GO: Gene Ontology
KEGG: Kyoto Encyclopedia of Genes and Genomes
BH: Benjamini-Hochberg
GEO: Gene Expression Omnibus
HSIL: high grade squamous intraepithelial lesions
DEGs: differentially expressed genes
