# Supplementary material for: 6 Circulating miRNAs can be used as Non-invasive Biomarkers for the Detection of Cervical Lesions
Source: J Cancer. 2021 Jun 22;12(17):5106–13. doi: 10.7150/jca.51141 (PMC8317520; doi:10.7150/jca.51141)
Supplement: Supplementary file 1 — Supplementary figures and tables. [file jcav12p5106s1.pdf]

Figure S1. Flow chart of the study. DR: detection rate. FC: fold change. GO: Gene Ontology. AUC: area under curve. KEGG: Kyoto Encyclopedia of Genes and Genomes. GEO: Gene Expression Omnibus.

Figure S2. Independent validation of miRNAs and the classifier. A, ΔCt of 6 selected miRNAs in the independent cohort. *, $P < 0.05$. **, $P < 0.01$. ***, $P < 0.001$. B, Receiver operating curve of the classifier in the independent cohort. AUC: area under curve.

Figure S3. Venn of predicted target genes from 3 platforms.

Figure S4. GO enrichment analysis. GO: Gene Ontology.

Table S1. miRNA expression profile in screening stage.

Table S2. miRNA expression profile in validation stage.

Table S3. Efficacy of the classifier.

Table S4. miRNA expression profile in independent validation stage.

Table S5. Predicted target genes.

Table S6a. KEGG enrichment analysis. KEGG: Kyoto Encyclopedia of Genes and Genomes.

Table S6b. GO_BP enrichment analysis. GO: Gene Ontology. BP: Biological Process.

Table S6c. GO_CC enrichment analysis. CC: Cellular Component.

Table S6d. GO_MF enrichment analysis. MF: Molecular Function.

Table S7. GEO expression array datasets involved in this study. GEO: Gene Expression Omnibus.

Table S8a. Predicted target genes covered by the selected GEO datasets. GEO: Gene Expression Omnibus.

Table S8b. Expression profiles of predicted target genes of hsa-miR-26b-5p in GEO datasets.

Table S8c. Expression profiles of predicted target genes of hsa-miR-146b-5p in GEO datasets.

Table S8d. Expression profiles of predicted target genes of hsa-miR-191-5p in GEO datasets.

Table S8e. Expression profiles of predicted target genes of hsa-miR-484 in GEO datasets.

Table S8f. Expression profiles of predicted target genes of hsa-miR-574-3p in GEO datasets.

Table S8g. Expression profiles of predicted target genes of hsa-miR-625-3p in GEO datasets.

Table S9. Most likely target genes selected by differential analysis.

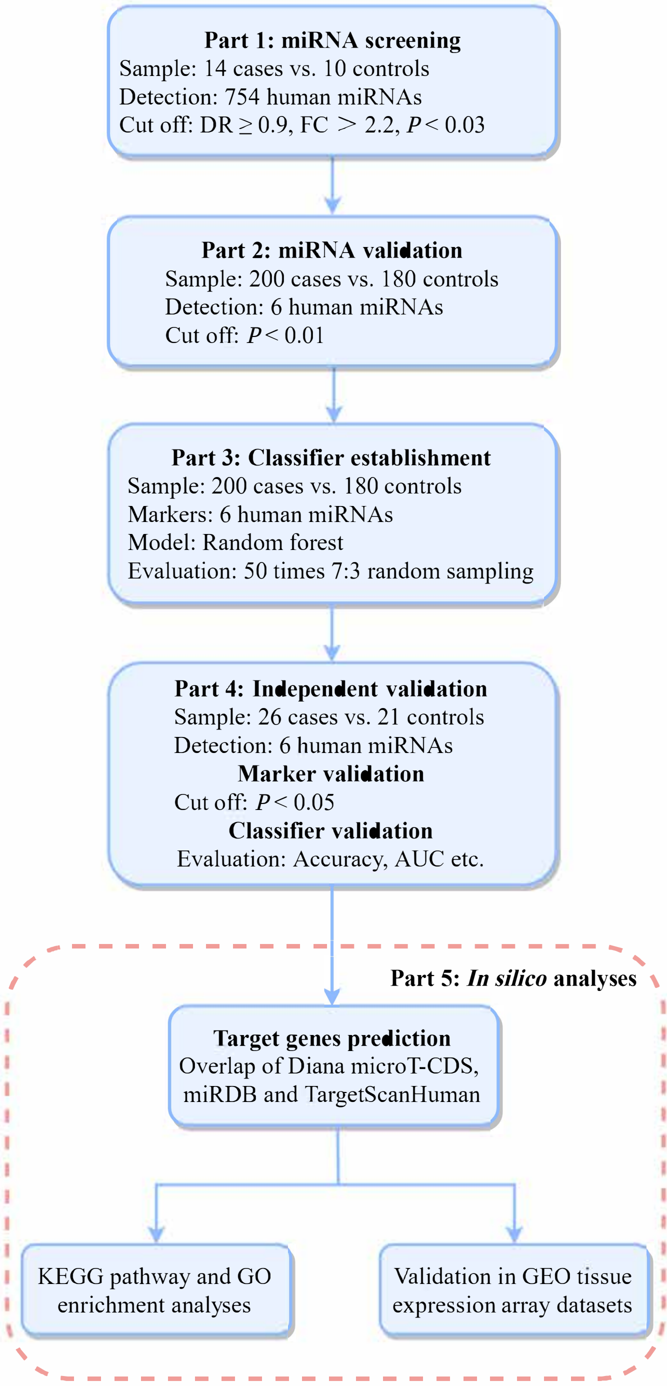

Part 1: miRNA screening
Sample: 14 cases vs. 10 controls
Detection: 754 human miRNAs
Cut off: DR ≥ 0.9, FC > 2.2, P < 0.03
Part 2: miRNA validation
Sample: 200 cases vs. 180 controls
Detection: 6 human miRNAs
Cut off: P < 0.01
Part 3: Classifier establishment
Sample: 200 cases vs. 180 controls
Markers: 6 human miRNAs
Model: Random forest
Evaluation: 50 times 7:3 random sampling
Part 4: Independent validation
Sample: 26 cases vs. 21 controls
Detection: 6 human miRNAs
Marker validation
Cut off: P < 0.05
Classifier validation
Evaluation: Accuracy, AUC etc.
Part 5: In silico analyses
Target genes prediction
Overlap of Diana microT-CDS, miRDB and TargetScanHuman
KEGG pathway and GO enrichment analyses
Validation in GEO tissue expression array datasets

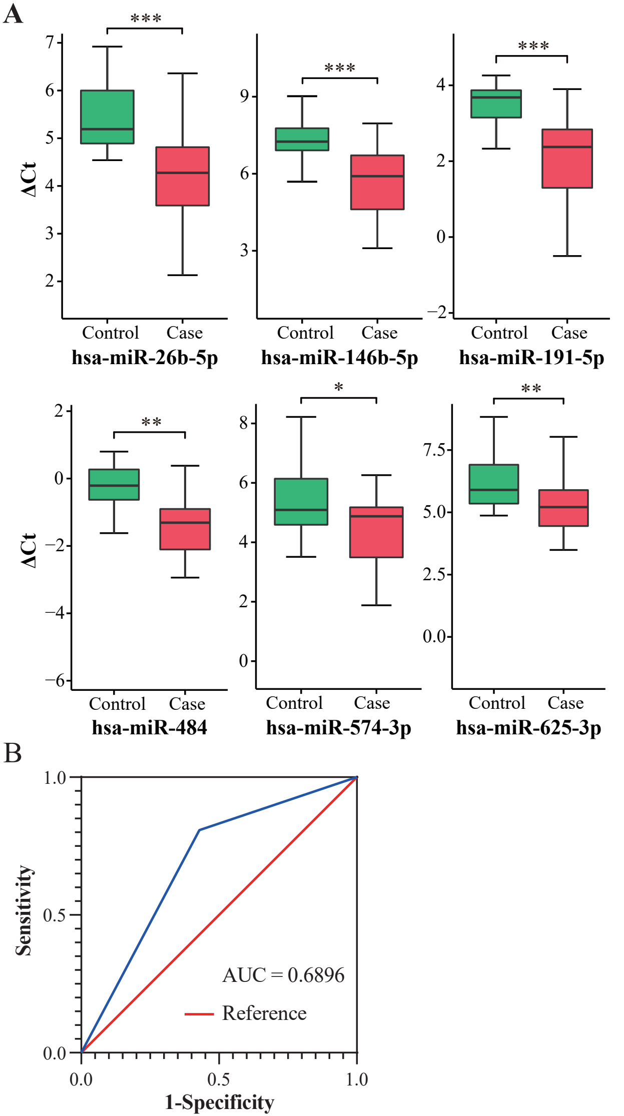
A
ΔCt
***
Control
Case
hsa-miR-26b-5p
***
hsa-miR-146b-5p
***
hsa-miR-191-5p
**
hsa-miR-484
*
hsa-miR-574-3p
**
hsa-miR-625-3p
B
Sensitivity
1-Specificity
AUC = 0.6896
Reference

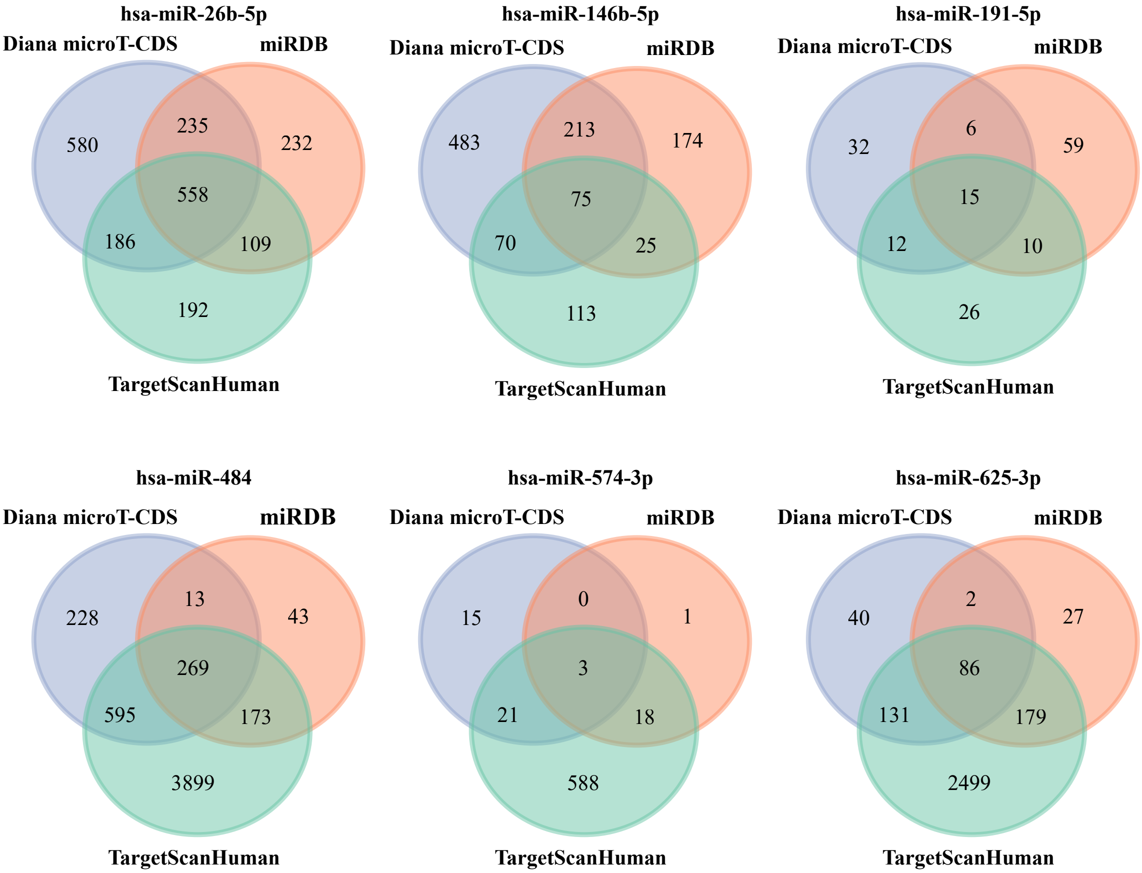
hsa-miR-26b-5p
Diana microT-CDS
miRDB
TargetScanHuman
580
235
232
558
186
109
192
hsa-miR-146b-5p
Diana microT-CDS
miRDB
TargetScanHuman
483
213
174
75
70
25
113
hsa-miR-191-5p
Diana microT-CDS
miRDB
TargetScanHuman
32
6
59
15
12
10
26
hsa-miR-484
Diana microT-CDS
miRDB
TargetScanHuman
228
13
43
269
595
173
3899
hsa-miR-574-3p
Diana microT-CDS
miRDB
TargetScanHuman
15
0
1
3
21
18
588
hsa-miR-625-3p
Diana microT-CDS
miRDB
TargetScanHuman
40
2
27
86
131
179
2499

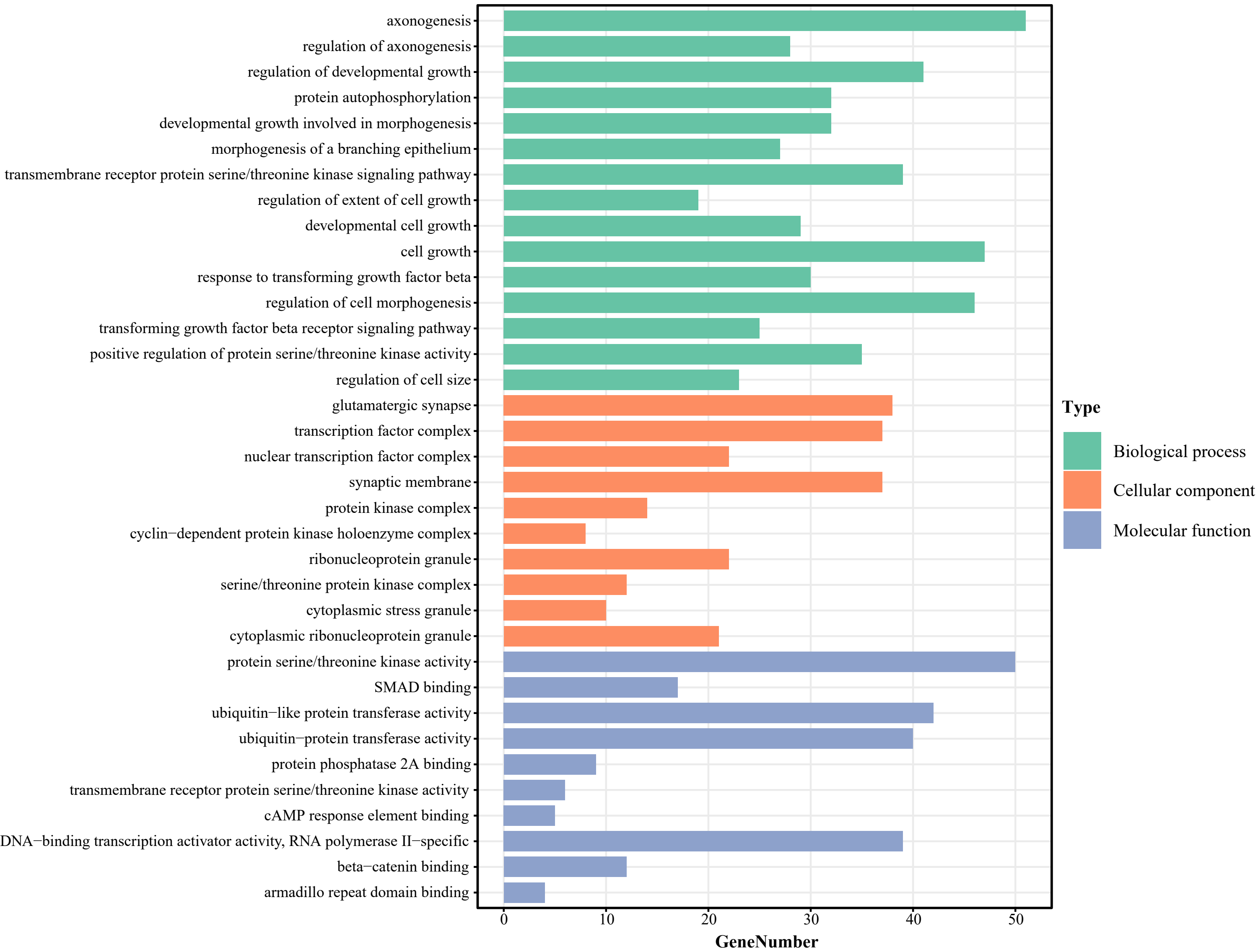
axonogenesis
regulation of axonogenesis
regulation of developmental growth
protein autophosphorylation
developmental growth involved in morphogenesis
morphogenesis of a branching epithelium
transmembrane receptor protein serine/threonine kinase signaling pathway
regulation of extent of cell growth
developmental cell growth
cell growth
response to transforming growth factor beta
regulation of cell morphogenesis
transforming growth factor beta receptor signaling pathway
positive regulation of protein serine/threonine kinase activity
regulation of cell size
glutamatergic synapse
transcription factor complex
nuclear transcription factor complex
synaptic membrane
protein kinase complex
cyclin−dependent protein kinase holoenzyme complex
ribonucleoprotein granule
serine/threonine protein kinase complex
cytoplasmic stress granule
cytoplasmic ribonucleoprotein granule
protein serine/threonine kinase activity
SMAD binding
ubiquitin−like protein transferase activity
ubiquitin−protein transferase activity
protein phosphatase 2A binding
transmembrane receptor protein serine/threonine kinase activity
cAMP response element binding
DNA−binding transcription activator activity, RNA polymerase II−specific
beta−catenin binding
armadillo repeat domain binding
0
10
20
30
40
50
GeneNumber
Type
Biological process
Cellular component
Molecular function

Table S1. miRNA expression profile in screening stage.

Detector	Original miRNA name	New miRNA name	Accession	Type	Detection Rate	Fold Change	P-value
ath-miR159a-000338	ath-miR159a	ath-miR159a	MIMAT0000177	Target	1	1.133772687	0.7577
dme-miR-7-000268	dme-miR-7	dme-miR-7-5p	MIMAT0000112	Target	0.5	0.755656268	0.6666
hsa-let-7a#-002307	hsa-let-7a*	hsa-let-7a-3p	MIMAT0004481	Target	0	2.404083395	0.0289
hsa-let-7a-000377	hsa-let-7a	hsa-let-7a-5p	MIMAT0000062	Target	0.208333333	628732.6712	0.3356
hsa-let-7b#-002404	hsa-let-7b*	hsa-let-7b-3p	MIMAT0004482	Target	0	2.404083395	0.0289
hsa-let-7b-002619	hsa-let-7b	hsa-let-7b	MI0000063	Target	1	3.086263855	0.0345
hsa-let-7c#-002405	hsa-let-7c*	NA	NA	Target	0	2.404083395	0.0289
hsa-let-7c-000379	hsa-let-7c	hsa-let-7c-5p	MIMAT0000064	Target	0.916666667	1.367581918	0.4511
hsa-let-7d-002283	hsa-let-7d	hsa-let-7d	MI0000065	Target	0.916666667	1.44756162	0.3529
hsa-let-7e#-002407	hsa-let-7e*	hsa-let-7e-3p	MIMAT0004485	Target	0	0.77074256	0.762
hsa-let-7e-002406	hsa-let-7e	hsa-let-7e	MI0000066	Target	0.916666667	3.262079086	0.0774
hsa-let-7f-000382	hsa-let-7f	hsa-let-7f-5p	MIMAT0000067	Target	0	2.404083395	0.0289
hsa-let-7f-1#-002417	hsa-let-7f-1*	hsa-let-7f-1-3p	MIMAT0004486	Target	0	2.404083395	0.0289
hsa-let-7f-2#-002418	hsa-let-7f-2*	hsa-let-7f-2-3p	MIMAT0004487	Target	0	2.404083395	0.0289
hsa-let-7g#-002118	hsa-let-7g*	hsa-let-7g-3p	MIMAT0004584	Target	0	2.404083395	0.0289
hsa-let-7g-002282	hsa-let-7g	hsa-let-7g	MI0000433	Target	0.791666667	3.553305646	0.0436
hsa-let-7i#-002172	hsa-let-7i*	hsa-let-7i-3p	MIMAT0004585	Target	0	0.737659929	0.6904
hsa-miR-1-002222	hsa-miR-1	hsa-miR-1	MIMAT0000416	Target	0.166666667	5.464879173	0.2253
hsa-miR-100#-002142	hsa-miR-100*	hsa-miR-100-3p	MIMAT0004512	Target	0.041666667	4107010.969	0.3356
hsa-miR-100-000437	hsa-miR-100	hsa-miR-100-5p	MIMAT0000098	Target	0.833333333	10.57828412	0.2246
hsa-miR-101#-002143	hsa-miR-101*	hsa-miR-101-5p	MIMAT0004513	Target	0	2.404083395	0.0289
hsa-miR-101-002253	hsa-miR-101	hsa-miR-101-3p	MIMAT0000099	Target	1	0.903312181	0.7233
hsa-miR-103-000439	hsa-miR-103	hsa-miR-103a-3p	MIMAT0000101	Target	0.208333333	905.2951517	0.0514
hsa-miR-105#-002168	hsa-miR-105*	hsa-miR-105-3p	MIMAT0004516	Target	0	2.404083395	0.0289
hsa-miR-105-002167	hsa-miR-105	hsa-miR-105-5p	MIMAT0000102	Target	0.041666667	0.624969934	0.6226
hsa-miR-106a#-002170	hsa-miR-106a*	hsa-miR-106a-3p	MIMAT0004517	Target	0	2.404083395	0.0289
hsa-miR-106a-002169	hsa-miR-106a	hsa-miR-106a-5p	MIMAT0000103	Target	0.958333333	1.874784653	0.0661
hsa-miR-106b#-002380	hsa-miR-106b*	hsa-miR-106b-3p	MIMAT0004672	Target	0	2.404083395	0.0289
hsa-miR-106b-000442	hsa-miR-106b	hsa-miR-106b-5p	MIMAT0000680	Target	1	2.412284193	0.0329
hsa-miR-107-000443	hsa-miR-107	hsa-miR-107	MIMAT0000104	Target	0.208333333	358554.3954	0.3356
hsa-miR-10a#-002288	hsa-miR-10a*	hsa-miR-10a-3p	MIMAT0004555	Target	0	1.813043681	0.1005
hsa-miR-10a-000387	hsa-miR-10a	hsa-miR-10a-5p	MIMAT0000253	Target	0.916666667	1.354430371	0.6048
hsa-miR-10b#-002315	hsa-miR-10b*	hsa-miR-10b-3p	MIMAT0004556	Target	1	3.763003274	0.2509
hsa-miR-10b-002218	hsa-miR-10b	hsa-miR-10b-5p	MIMAT0000254	Target	0.083333333	4.066337602	0.321
hsa-miR-1178-002777	hsa-miR-1178	hsa-miR-1178	MIMAT0005823	Target	0.041666667	5.02745E-09	0.3434
hsa-miR-1179-002776	hsa-miR-1179	hsa-miR-1179	MIMAT0005824	Target	0	2.404083395	0.0289
hsa-miR-1180-002847	hsa-miR-1180	hsa-miR-1180	MIMAT0005825	Target	0.541666667	0.800881207	0.6137
hsa-miR-1182-002830	hsa-miR-1182	hsa-miR-1182	MIMAT0005827	Target	0	2.404083395	0.0289
hsa-miR-1183-002841	hsa-miR-1183	hsa-miR-1183	MIMAT0005828	Target	0.041666667	10.02590674	0.2497
hsa-miR-1184-002842	hsa-miR-1184	hsa-miR-1184	MIMAT0005829	Target	0	2.404083395	0.0289
hsa-miR-1197-002810	hsa-miR-1197	hsa-miR-1197	MIMAT0005955	Target	0	2.404083395	0.0289
hsa-miR-1200-002829	hsa-miR-1200	hsa-miR-1200	MIMAT0005863	Target	0	2.404083395	0.0289
hsa-miR-1201-002781	ath-miR-1201	NA	NA	Target	0	2.404083395	0.0289
hsa-miR-1203-002877	hsa-miR-1203	hsa-miR-1203	MIMAT0005866	Target	0	2.404083395	0.0289
hsa-miR-1204-002872	hsa-miR-1204	hsa-miR-1204	MIMAT0005868	Target	0	2.404083395	0.0289

hsa-miR-1205-002778	hsa-miR-1205	hsa-miR-1205	MIMAT0005869	Target	0	2.404083395	0.0289
hsa-miR-1206-002878	hsa-miR-1206	hsa-miR-1206	MIMAT0005870	Target	0	2.404083395	0.0289
hsa-miR-1208-002880	hsa-miR-1208	hsa-miR-1208	MIMAT0005873	Target	0	2.404083395	0.0289
hsa-miR-122#-002130	hsa-miR-122*	hsa-miR-122-3p	MIMAT0004590	Target	0.083333333	42991014.56	0.3356
hsa-miR-122-002245	hsa-miR-122	hsa-miR-122-5p	MIMAT0000421	Target	0.833333333	5.108117099	0.0909
hsa-miR-1224-3p-002752	hsa-miR-1224-3p	NA	NA	Target	0	2.404083395	0.0289
hsa-miR-1225-3p-002766	hsa-miR-1225-3p	NA	NA	Target	0	2.404083395	0.0289
hsa-miR-1226#-002758	hsa-miR-1226*	hsa-miR-1226-5p	MIMAT0005576	Target	0	2.404083395	0.0289
hsa-miR-1227-002769	hsa-miR-1227	hsa-miR-1227	MIMAT0005580	Target	0.833333333	3.947638752	0.1688
hsa-miR-1228#-002763	hsa-miR-1228*	hsa-miR-1228-5p	MIMAT0005582	Target	0	2.404083395	0.0289
hsa-miR-1233-002768	hsa-miR-1233	hsa-miR-1233	MIMAT0005588	Target	0.083333333	3.141540841	0.5254
hsa-miR-1236-002761	hsa-miR-1236	hsa-miR-1236	MIMAT0005591	Target	0	2.404083395	0.0289
hsa-miR-1238-002927	hsa-miR-1238	hsa-miR-1238	MIMAT0005593	Target	0	2.404083395	0.0289
hsa-miR-124#-002197	hsa-miR-124*	hsa-miR-124-5p	MIMAT0004591	Target	0	2.404083395	0.0289
hsa-miR-1243-002854	hsa-miR-1243	hsa-miR-1243	MIMAT0005894	Target	1	2.155820302	0.1256
hsa-miR-1244-002791	hsa-miR-1244	hsa-miR-1244	MIMAT0005896	Target	0	1.232086195	0.8028
hsa-miR-1245-002823	hsa-miR-1245	hsa-miR-1245a	MIMAT0005897	Target	0	2.404083395	0.0289
hsa-miR-1247-002893	hsa-miR-1247	hsa-miR-1247-5p	MIMAT0005899	Target	0	2.404083395	0.0289
hsa-miR-1248-002870	hsa-miR-1248	hsa-miR-1248	MIMAT0005900	Target	0	1.865903868	0.0888
hsa-miR-1249-002868	hsa-miR-1249	hsa-miR-1249	MIMAT0005901	Target	0.166666667	0.354911409	0.407
hsa-miR-1250-002887	hsa-miR-1250	hsa-miR-1250	MIMAT0005902	Target	0	2.404083395	0.0289
hsa-miR-1251-002820	hsa-miR-1251	hsa-miR-1251	MIMAT0005903	Target	0	2.404083395	0.0289
hsa-miR-1252-002860	hsa-miR-1252	hsa-miR-1252	MIMAT0005944	Target	0	2.404083395	0.0289
hsa-miR-1253-002894	hsa-miR-1253	hsa-miR-1253	MIMAT0005904	Target	0	2.404083395	0.0289
hsa-miR-1254-002818	hsa-miR-1254	hsa-miR-1254	MIMAT0005905	Target	0.041666667	0.478922572	0.574
hsa-miR-1255A-002805	hsa-miR-1255A	NA	NA	Target	0	2.404083395	0.0289
hsa-miR-1255B-002801	hsa-miR-1255B	NA	NA	Target	0.583333333	3.168945765	0.1861
hsa-miR-1256-002850	hsa-miR-1256	hsa-miR-1256	MIMAT0005907	Target	0	0.547639564	0.4644
hsa-miR-1257-002910	hsa-miR-1257	hsa-miR-1257	MIMAT0005908	Target	0	2.404083395	0.0289
hsa-miR-1259-002796	hsa-miR-1259	NA	NA	Target	0	2.404083395	0.0289
hsa-miR-125a-3p-002199	hsa-miR-125a-3p	hsa-miR-125a-3p	MIMAT0004602	Target	0.083333333	5.20599E-07	0.3434
hsa-miR-125a-5p-002198	hsa-miR-125a-5p	hsa-miR-125a-5p	MIMAT0000443	Target	0.208333333	3.24735E-07	0.3434
hsa-miR-125b-000449	hsa-miR-125b	hsa-miR-125b-5p	MIMAT0000423	Target	0.875	1.748242186	0.2524
hsa-miR-125b-1#-002378	hsa-miR-125b-1*	hsa-miR-125b-1-3p	MIMAT0004592	Target	0.125	4.036105833	0.3129
hsa-miR-125b-2#-002158	hsa-miR-125b-2*	hsa-miR-125b-2-3p	MIMAT0004603	Target	0	2.404083395	0.0289
hsa-miR-126#-000451	hsa-miR-126*	hsa-miR-126-5p	MIMAT0000444	Target	1	1.090875792	0.7478
hsa-miR-126-002228	hsa-miR-126	hsa-miR-126-3p	MIMAT0000445	Target	1	1.768291552	0.2906
hsa-miR-1260-002896	hsa-miR-1260	hsa-miR-1260a	MIMAT0005911	Target	0.083333333	997.5931409	0.1652
hsa-miR-1262-002852	hsa-miR-1262	hsa-miR-1262	MIMAT0005914	Target	0	4.106217617	0.1195
hsa-miR-1263-002784	hsa-miR-1263	hsa-miR-1263	MIMAT0005915	Target	0	2.404083395	0.0289
hsa-miR-1264-002799	hsa-miR-1264	hsa-miR-1264	MIMAT0005791	Target	0	2.404083395	0.0289
hsa-miR-1265-002790	hsa-miR-1265	hsa-miR-1265	MIMAT0005918	Target	0.041666667	9.531828275	0.2464
hsa-miR-1267-002885	hsa-miR-1267	hsa-miR-1267	MIMAT0005921	Target	0	8.577288428	0.2626
hsa-miR-1269-002789	hsa-miR-1269	hsa-miR-1269a	MIMAT0005923	Target	0	2.404083395	0.0289
hsa-miR-127-000452	hsa-miR-127	hsa-miR-127-3p	MIMAT0000446	Target	0.333333333	0.638869678	0.5897
hsa-miR-127-5p-002229	hsa-miR-127-5p	hsa-miR-127-5p	MIMAT0004604	Target	0.041666667	198.3197631	0.3324
hsa-miR-1270-002807	hsa-miR-1270	hsa-miR-1270	MIMAT0005924	Target	0.083333333	0.804268516	0.8021

hsa-miR-1271-002779	hsa-miR-1271	hsa-miR-1271-5p	MIMAT0005796	Target	0.083333333	0.541226097	0.3824
hsa-miR-1272-002845	hsa-miR-1272	hsa-miR-1272	MIMAT0005925	Target	0	2.404083395	0.0289
hsa-miR-1274A-002883	hsa-miR-1274A	NA	NA	Target	1	5.749992295	0.0861
hsa-miR-1274B-002884	hsa-miR-1274B	NA	NA	Target	1	6.28281396	0.1557
hsa-miR-1275-002840	hsa-miR-1275	hsa-miR-1275	MIMAT0005929	Target	0.083333333	0.001426595	0.2436
hsa-miR-1276-002843	hsa-miR-1276	hsa-miR-1276	MIMAT0005930	Target	0.25	1.49742311	0.5278
hsa-miR-1278-002851	hsa-miR-1278	hsa-miR-1278	MIMAT0005936	Target	0.041666667	116841652	0.3356
hsa-miR-1282-002803	hsa-miR-1282	hsa-miR-1282	MIMAT0005940	Target	0	3.522624092	0.2606
hsa-miR-1283-002890	hsa-miR-1283	hsa-miR-1283	MIMAT0005799	Target	0	2.404083395	0.0289
hsa-miR-1284-002903	hsa-miR-1284	hsa-miR-1284	MIMAT0005941	Target	0	2.404083395	0.0289
hsa-miR-1285-002822	hsa-miR-1285	hsa-miR-1285-3p	MIMAT0005876	Target	0.541666667	7.710951932	0.3319
hsa-miR-1286-002773	hsa-miR-1286	hsa-miR-1286	MIMAT0005877	Target	0	2.404083395	0.0289
hsa-miR-1288-002832	hsa-miR-1288	hsa-miR-1288	MIMAT0005942	Target	0	2.404083395	0.0289
hsa-miR-1289-002871	hsa-miR-1289	hsa-miR-1289	MIMAT0005879	Target	0	1.142124542	0.8128
hsa-miR-128a-002216	hsa-miR-128a	hsa-miR-128	MIMAT0000424	Target	0.916666667	2.690599528	0.1039
hsa-miR-129#-002298	hsa-miR-129*	hsa-miR-129-1-3p	MIMAT0004548	Target	0	6.299654577	0.2121
hsa-miR-129-000590	hsa-miR-129	hsa-miR-129-5p	MIMAT0000242	Target	0.041666667	1080062308	0.3356
hsa-miR-1290-002863	hsa-miR-1290	hsa-miR-1290	MIMAT0005880	Target	0.666666667	7.281695953	0.1046
hsa-miR-1291-002838	hsa-miR-1291	hsa-miR-1291	MIMAT0005881	Target	0.916666667	2.350833344	0.3015
hsa-miR-1292-002824	hsa-miR-1292	hsa-miR-1292	MIMAT0005943	Target	0	2.404083395	0.0289
hsa-miR-1293-002905	hsa-miR-1293	hsa-miR-1293	MIMAT0005883	Target	0	2.404083395	0.0289
hsa-miR-1294-002785	hsa-miR-1294	hsa-miR-1294	MIMAT0005884	Target	0	2.404083395	0.0289
hsa-miR-1296-002908	hsa-miR-1296	hsa-miR-1296	MIMAT0005794	Target	0	2.404083395	0.0289
hsa-miR-1298-002861	hsa-miR-1298	hsa-miR-1298	MIMAT0005800	Target	0	2.404083395	0.0289
hsa-miR-1300-002902	hsa-miR-1300	NA	NA	Target	0.458333333	4.391932497	0.2472
hsa-miR-1301-002827	hsa-miR-1301	hsa-miR-1301	MIMAT0005797	Target	0	2.404083395	0.0289
hsa-miR-1302-002901	hsa-miR-1302	hsa-miR-1302	MIMAT0005890	Target	0	2.404083395	0.0289
hsa-miR-1303-002792	hsa-miR-1303	hsa-miR-1303	MIMAT0005891	Target	0.625	4.821354287	0.3259
hsa-miR-1304-002874	hsa-miR-1304	hsa-miR-1304-5p	MIMAT0005892	Target	0	2.404083395	0.0289
hsa-miR-1305-002867	hsa-miR-1305	hsa-miR-1305	MIMAT0005893	Target	0.166666667	0.968886281	0.9693
hsa-miR-130a#-002131	hsa-miR-130a*	hsa-miR-130a-5p	MIMAT0004593	Target	0	2.404083395	0.0289
hsa-miR-130a-000454	hsa-miR-130a	hsa-miR-130a-3p	MIMAT0000425	Target	1	0.326992676	0.4194
hsa-miR-130b#-002114	hsa-miR-130b*	hsa-miR-130b-5p	MIMAT0004680	Target	0.041666667	337363207.5	0.3356
hsa-miR-130b-000456	hsa-miR-130b	hsa-miR-130b-3p	MIMAT0000691	Target	1	1.274204185	0.6399
hsa-miR-132#-002132	hsa-miR-132*	hsa-miR-132-5p	MIMAT0004594	Target	0.041666667	0.003566384	0.3443
hsa-miR-132-000457	hsa-miR-132	hsa-miR-132-3p	MIMAT0000426	Target	0.958333333	1.510683949	0.4508
hsa-miR-1324-002815	hsa-miR-1324	hsa-miR-1324	MIMAT0005956	Target	0	2.404083395	0.0289
hsa-miR-133a-002246	hsa-miR-133a	hsa-miR-133a	MIMAT0000427	Target	0.916666667	2.01896635	0.1382
hsa-miR-133b-002247	hsa-miR-133b	hsa-miR-133b	MIMAT0000770	Target	0.041666667	0.517173099	0.5651
hsa-miR-135a-000460	hsa-miR-135a	hsa-miR-135a-5p	MIMAT0000428	Target	0.333333333	0.839479065	0.7544
hsa-miR-135b#-002159	hsa-miR-135b*	hsa-miR-135b-3p	MIMAT0004698	Target	0	2.404083395	0.0289
hsa-miR-135b-002261	hsa-miR-135b	hsa-miR-135b-5p	MIMAT0000758	Target	0.041666667	0.916894665	0.9192
hsa-miR-136#-002100	hsa-miR-136*	hsa-miR-136-3p	MIMAT0004606	Target	0.125	0.93050657	0.9061
hsa-miR-136-000592	hsa-miR-136	hsa-miR-136-5p	MIMAT0000448	Target	0.291666667	1.076359732	0.9036
hsa-miR-138-002284	hsa-miR-138	hsa-miR-138-5p	MIMAT0000430	Target	0.166666667	0.808963044	0.7758
hsa-miR-138-2#-002144	hsa-miR-138-2*	hsa-miR-138-2-3p	MIMAT0004596	Target	0	2.404083395	0.0289
hsa-miR-139-3p-002313	hsa-miR-139-3p	hsa-miR-139-3p	MIMAT0004552	Target	0.083333333	506.7286578	0.1677

hsa-miR-139-5p-002289	hsa-miR-139-5p	hsa-miR-139-5p	MIMAT0000250	Target	1	1.771881849	0.2035
hsa-miR-140-3p-002234	hsa-miR-140-3p	hsa-miR-140-3p	MIMAT0004597	Target	0.75	1.730062839	0.2756
hsa-miR-141#-002145	hsa-miR-141*	hsa-miR-141-5p	MIMAT0004598	Target	0.041666667	644276.3587	0.3356
hsa-miR-141-000463	hsa-miR-141	hsa-miR-141-3p	MIMAT0000432	Target	0.333333333	10.63791843	0.0631
hsa-miR-142-3p-000464	hsa-miR-142-3p	hsa-miR-142-3p	MIMAT0000434	Target	0.875	1.63689162	0.3624
hsa-miR-142-5p-002248	hsa-miR-142-5p	hsa-miR-142-5p	MIMAT0000433	Target	0.291666667	1.892776274	0.3804
hsa-miR-143#-002146	hsa-miR-143*	hsa-miR-143-5p	MIMAT0004599	Target	0	2.404083395	0.0289
hsa-miR-143-002249	hsa-miR-143	hsa-miR-143-3p	MIMAT0000435	Target	0.708333333	2.824122934	0.137
hsa-miR-144#-002148	hsa-miR-144*	hsa-miR-144-5p	MIMAT0004600	Target	1	1.08684784	0.8593
hsa-miR-144-002676	hsa-miR-144	hsa-miR-144-3p	MIMAT0000436	Target	0.083333333	1.683370519	0.7286
hsa-miR-145#-002149	hsa-miR-145*	hsa-miR-145-3p	MIMAT0004601	Target	0.708333333	1.38380376	0.4503
hsa-miR-145-002278	hsa-miR-145	hsa-miR-145-5p	MIMAT0000437	Target	0.75	3.872321431	0.3256
hsa-miR-146a#-002163	hsa-miR-146a*	hsa-miR-146a-3p	MIMAT0004608	Target	0	2.404083395	0.0289
hsa-miR-146a-000468	hsa-miR-146a	hsa-miR-146a-5p	MIMAT0000449	Target	1	1.572561988	0.2262
hsa-miR-146b-001097	hsa-miR-146b	hsa-miR-146b-5p	MIMAT0002809	Target	0.916666667	3.853801754	0.0037
hsa-miR-146b-3p-002361	hsa-miR-146b-3p	hsa-miR-146b-3p	MIMAT0004766	Target	0.125	5647509.168	0.3356
hsa-miR-147-000469	hsa-miR-147	hsa-miR-147a	MIMAT0000251	Target	0	0.780358394	0.6791
hsa-miR-147b-002262	hsa-miR-147b	hsa-miR-147b	MIMAT0004928	Target	0.083333333	7.26929E-06	0.1728
hsa-miR-148a#-002134	hsa-miR-148a*	hsa-miR-148a-5p	MIMAT0004549	Target	0	2.404083395	0.0289
hsa-miR-148a-000470	hsa-miR-148a	hsa-miR-148a-3p	MIMAT0000243	Target	1	1.225266294	0.7187
hsa-miR-148b#-002160	hsa-miR-148b*	hsa-miR-148b-5p	MIMAT0004699	Target	0.041666667	1.750188145	0.5312
hsa-miR-148b-000471	hsa-miR-148b	hsa-miR-148b-3p	MIMAT0000759	Target	0.666666667	0.694442216	0.4323
hsa-miR-149#-002164	hsa-miR-149*	hsa-miR-149-3p	MIMAT0004609	Target	0	2.404083395	0.0289
hsa-miR-149-002255	hsa-miR-149	hsa-miR-149-5p	MIMAT0000450	Target	0	2.404083395	0.0289
hsa-miR-150-000473	hsa-miR-150	hsa-miR-150-5p	MIMAT0000451	Target	1	1.946755252	0.0422
hsa-miR-151-3p-002254	hsa-miR-151-3p	hsa-miR-151a-3p	MIMAT0000757	Target	1	1.621387462	0.0456
hsa-miR-151-5P-002642	hsa-miR-151-5p	NA	NA	Target	0.916666667	1.224860768	0.4937
hsa-miR-152-000475	hsa-miR-152	hsa-miR-152	MIMAT0000438	Target	0.875	0.883214523	0.7285
hsa-miR-154#-000478	hsa-miR-154*	hsa-miR-154-3p	MIMAT0000453	Target	0	2.404083395	0.0289
hsa-miR-154-000477	hsa-miR-154	hsa-miR-154-5p	MIMAT0000452	Target	0	2.404083395	0.0289
hsa-miR-155#-002287	hsa-miR-155*	hsa-miR-155-3p	MIMAT0004658	Target	0	2.404083395	0.0289
hsa-miR-155-002623	hsa-miR-155	hsa-miR-155-5p	MIMAT0000646	Target	0.625	1.160457098	0.8464
hsa-miR-15a#-002419	hsa-miR-15a*	hsa-miR-15a-3p	MIMAT0004488	Target	0.458333333	0.315417156	0.1256
hsa-miR-15a-000389	hsa-miR-15a	hsa-miR-15a-5p	MIMAT0000068	Target	0.083333333	268.1442758	0.3071
hsa-miR-15b#-002173	hsa-miR-15b*	hsa-miR-15b-3p	MIMAT0004586	Target	0	2.404083395	0.0289
hsa-miR-15b-000390	hsa-miR-15b	hsa-miR-15b-5p	MIMAT0000417	Target	0.958333333	0.756965701	0.5381
hsa-miR-16-000391	hsa-miR-16	hsa-miR-16-5p	MIMAT0000069	Selected Control	1	1	1
hsa-miR-16-1#-002420	hsa-miR-16-1*	hsa-miR-16-1-3p	MIMAT0004489	Target	0.125	0.305604372	0.4655
hsa-miR-16-2#-002171	hsa-miR-16-2*	hsa-miR-16-2-3p	MIMAT0004518	Target	0	2.404083395	0.0289
hsa-miR-17#-002421	hsa-miR-17*	hsa-miR-17-3p	MIMAT0000071	Target	0	7.815710971	0.1255
hsa-miR-17-002308	hsa-miR-17	hsa-miR-17-5p	MIMAT0000070	Target	1	1.224951926	0.4631
hsa-miR-181a-000480	hsa-miR-181a	hsa-miR-181a-5p	MIMAT0000256	Target	0.916666667	1.116673961	0.7933
hsa-miR-181a-2#-002317	hsa-miR-181a-2*	hsa-miR-181a-2-3p	MIMAT0004558	Target	0.333333333	5.4004718	0.1232
hsa-miR-181c#-002333	hsa-miR-181c*	hsa-miR-181c-3p	MIMAT0004559	Target	0	2.404083395	0.0289
hsa-miR-181c-000482	hsa-miR-181c	hsa-miR-181c-5p	MIMAT0000258	Target	0.083333333	0.289502165	0.1827
hsa-miR-182#-000483	hsa-miR-182*	hsa-miR-182-3p	MIMAT0000260	Target	0.041666667	53676482963	0.3356
hsa-miR-182-002334	hsa-miR-182	hsa-miR-182-5p	MIMAT0000259	Target	0.083333333	2.81263E-05	0.3434

hsa-miR-1825-002907	hsa-miR-1825	hsa-miR-1825	MIMAT0006765	Target	0.083333333	1043.386072	0.1869
hsa-miR-1826-002873	hsa-miR-1826	NA	NA	Target	0	2.404083395	0.0289
hsa-miR-183#-002270	hsa-miR-183*	hsa-miR-183-3p	MIMAT0004560	Target	0.25	0.364923119	0.2285
hsa-miR-183-002269	hsa-miR-183	hsa-miR-183-5p	MIMAT0000261	Target	0.125	0.334349936	0.3572
hsa-miR-184-000485	hsa-miR-184	hsa-miR-184	MIMAT0000454	Target	0.75	3.9958E-07	0.3434
hsa-miR-185#-002104	hsa-miR-185*	hsa-miR-185-3p	MIMAT0004611	Target	0	2.404083395	0.0289
hsa-miR-185-002271	hsa-miR-185	hsa-miR-185-5p	MIMAT0000455	Target	1	2.400236192	0.366
hsa-miR-186#-002105	hsa-miR-186*	hsa-miR-186-3p	MIMAT0004612	Target	0	2.404083395	0.0289
hsa-miR-186-002285	hsa-miR-186	hsa-miR-186-5p	MIMAT0000456	Target	0.875	0.359852018	0.5521
hsa-miR-188-3p-002106	hsa-miR-188-3p	hsa-miR-188-3p	MIMAT0004613	Target	0.041666667	323.0955465	0.3336
hsa-miR-18a#-002423	hsa-miR-18a*	hsa-miR-18a-3p	MIMAT0002891	Target	0.083333333	2.134669184	0.2889
hsa-miR-18a-002422	hsa-miR-18a	hsa-miR-18a-5p	MIMAT0000072	Target	0.625	4.765470481	0.0805
hsa-miR-18b#-002310	hsa-miR-18b*	hsa-miR-18b-3p	MIMAT0004751	Target	0	2.404083395	0.0289
hsa-miR-18b-002217	hsa-miR-18b	hsa-miR-18b-5p	MIMAT0001412	Target	0.083333333	0.30439637	0.5196
hsa-miR-190-000489	hsa-miR-190	hsa-miR-190a	MIMAT0000458	Target	0.041666667	0.605220894	0.5084
hsa-miR-190b-002263	hsa-miR-190b	hsa-miR-190b	MIMAT0004929	Target	0.333333333	1.556668137	0.4321
hsa-miR-191#-002678	hsa-miR-191*	hsa-miR-191-3p	MIMAT0001618	Target	0	1.123449935	0.8449
hsa-miR-191-002299	hsa-miR-191	hsa-miR-191-5p	MIMAT0000440	Target	1	2.258403154	0.028
hsa-miR-192#-002272	hsa-miR-192*	hsa-miR-192-3p	MIMAT0004543	Target	0.041666667	2.397030021	0.1987
hsa-miR-192-000491	hsa-miR-192	hsa-miR-192-5p	MIMAT0000222	Target	1	1.637705556	0.3049
hsa-miR-193a-3p-002250	hsa-miR-193a-3p	hsa-miR-193a-3p	MIMAT0000459	Target	0.041666667	38.95046879	0.3165
hsa-miR-193a-5p-002281	hsa-miR-193a-5p	hsa-miR-193a-5p	MIMAT0004614	Target	0.916666667	3.401011801	0.0624
hsa-miR-193b#-002366	hsa-miR-193b*	hsa-miR-193b-5p	MIMAT0004767	Target	0.083333333	0.192793918	0.445
hsa-miR-193b-002367	hsa-miR-193b	hsa-miR-193b-3p	MIMAT0002819	Target	0.875	1.130191004	0.8725
hsa-miR-194#-002379	hsa-miR-194*	hsa-miR-194-3p	MIMAT0004671	Target	0	2.404083395	0.0289
hsa-miR-194-000493	hsa-miR-194	hsa-miR-194-5p	MIMAT0000460	Target	0.666666667	169789881.5	0.3356
hsa-miR-195#-002107	hsa-miR-195*	hsa-miR-195-3p	MIMAT0004615	Target	0	2.404083395	0.0289
hsa-miR-195-000494	hsa-miR-195	hsa-miR-195-5p	MIMAT0000461	Target	1	1.123133682	0.6166
hsa-miR-196a#-002336	hsa-miR-196a*	hsa-miR-196a-3p	MIMAT0004562	Target	0	2.404083395	0.0289
hsa-miR-196b-002215	hsa-miR-196b	hsa-miR-196b-5p	MIMAT0001080	Target	0.083333333	1.491741005	0.4949
hsa-miR-197-000497	hsa-miR-197	hsa-miR-197-3p	MIMAT0000227	Target	1	0.329005597	0.4676
hsa-miR-198-002273	hsa-miR-198	hsa-miR-198	MIMAT0000228	Target	0	2.404083395	0.0289
hsa-miR-199a-000498	hsa-miR-199a	hsa-miR-199a-5p	MIMAT0000231	Target	0.041666667	18.99144208	0.3211
hsa-miR-199a-3p-002304	hsa-miR-199a-3p	hsa-miR-199a-3p	MIMAT0000232	Target	0.958333333	1.804027256	0.1287
hsa-miR-199b-000500	hsa-miR-199b	hsa-miR-199b-5p	MIMAT0000263	Target	0	2.404083395	0.0289
hsa-miR-19a#-002424	hsa-miR-19a*	hsa-miR-19a-5p	MIMAT0004490	Target	0	2.404083395	0.0289
hsa-miR-19a-000395	hsa-miR-19a	hsa-miR-19a-3p	MIMAT0000073	Target	0.791666667	9.45336E-05	0.3435
hsa-miR-19b-000396	hsa-miR-19b	hsa-miR-19b-3p	MIMAT0000074	Target	1	1.00698352	0.9775
hsa-miR-19b-1#-002425	hsa-miR-19b-1*	hsa-miR-19b-1-5p	MIMAT0004491	Target	0.625	1.876736403	0.2295
hsa-miR-200a#-001011	hsa-miR-200a*	hsa-miR-200a-5p	MIMAT0001620	Target	0	2.404083395	0.0289
hsa-miR-200a-000502	hsa-miR-200a	hsa-miR-200a-3p	MIMAT0000682	Target	0	5.716444609	0.177
hsa-miR-200b#-002274	hsa-miR-200b*	hsa-miR-200b-5p	MIMAT0004571	Target	0	2.404083395	0.0289
hsa-miR-200b-002251	hsa-miR-200b	hsa-miR-200b-3p	MIMAT0000318	Target	0	2.404083395	0.0289
hsa-miR-200c#-002286	hsa-miR-200c*	hsa-miR-200c-5p	MIMAT0004657	Target	0	2.404083395	0.0289
hsa-miR-200c-002300	hsa-miR-200c	hsa-miR-200c-3p	MIMAT0000617	Target	0.208333333	14.47787068	0.1122
hsa-miR-202#-002362	hsa-miR-202*	hsa-miR-202-5p	MIMAT0002810	Target	0	2.404083395	0.0289
hsa-miR-202-002363	hsa-miR-202	hsa-miR-202-3p	MIMAT0002811	Target	0.208333333	0.598960553	0.2726

hsa-miR-203-000507	hsa-miR-203	hsa-miR-203	MIMAT0000264	Target	0.875	4.251081613	0.1066
hsa-miR-204-000508	hsa-miR-204	hsa-miR-204-5p	MIMAT0000265	Target	0.916666667	1.755098767	0.1495
hsa-miR-205-000509	hsa-miR-205	hsa-miR-205-5p	MIMAT0000266	Target	0.166666667	3.160523171	0.2878
hsa-miR-206-000510	hsa-miR-206	hsa-miR-206	MIMAT0000462	Target	0.541666667	1.697223603	0.4372
hsa-miR-208-000511	hsa-miR-208	hsa-miR-208a	MIMAT0000241	Target	0.208333333	3.541374593	0.3611
hsa-miR-208b-002290	hsa-miR-208b	hsa-miR-208b	MIMAT0004960	Target	0	1.735339535	0.1706
hsa-miR-20a#-002437	hsa-miR-20a*	hsa-miR-20a-3p	MIMAT0004493	Target	0.041666667	1.065805201	0.9099
hsa-miR-20a-000580	hsa-miR-20a	hsa-miR-20a-5p	MIMAT0000075	Target	1	1.798326844	0.0866
hsa-miR-20b#-002311	hsa-miR-20b*	hsa-miR-20b-3p	MIMAT0004752	Target	0	2.404083395	0.0289
hsa-miR-20b-001014	hsa-miR-20b	hsa-miR-20b-5p	MIMAT0001413	Target	1	1.331388398	0.3331
hsa-miR-21#-002438	hsa-miR-21*	hsa-miR-21-3p	MIMAT0004494	Target	0	2.404083395	0.0289
hsa-miR-21-000397	hsa-miR-21	hsa-miR-21-5p	MIMAT0000076	Target	0.916666667	0.675457267	0.4131
hsa-miR-210-000512	hsa-miR-210	hsa-miR-210	MIMAT0000267	Target	0.125	2085.586603	0.2928
hsa-miR-211-000514	hsa-miR-211	hsa-miR-211-5p	MIMAT0000268	Target	0.25	0.227203517	0.1504
hsa-miR-212-000515	hsa-miR-212	hsa-miR-212-3p	MIMAT0000269	Target	0.291666667	9.730045356	0.1087
hsa-miR-213-000516	hsa-miR-213	hsa-miR-181a-3p	MIMAT0000270	Target	0.041666667	198423603.4	0.3356
hsa-miR-214#-002293	hsa-miR-214*	hsa-miR-214-5p	MIMAT0004564	Target	0.041666667	1.520524865	0.5048
hsa-miR-214-002306	hsa-miR-214	hsa-miR-214-3p	MIMAT0000271	Target	0.708333333	0.775214836	0.7101
hsa-miR-215-000518	hsa-miR-215	hsa-miR-215	MIMAT0000272	Target	0.708333333	1.973452993	0.2743
hsa-miR-216a-002220	hsa-miR-216a	hsa-miR-216a	MIMAT0000273	Target	0	2.404083395	0.0289
hsa-miR-216b-002326	hsa-miR-216b	hsa-miR-216b	MIMAT0004959	Target	0	2.787749815	0.0101
hsa-miR-217-002337	hsa-miR-217	hsa-miR-217	MIMAT0000274	Target	0	2.404083395	0.0289
hsa-miR-218-000521	hsa-miR-218	hsa-miR-218-5p	MIMAT0000275	Target	0.666666667	1.651564012	0.5194
hsa-miR-218-1#-002094	hsa-miR-218-1*	hsa-miR-218-1-3p	MIMAT0004565	Target	0	2.404083395	0.0289
hsa-miR-218-2#-002294	hsa-miR-218-2*	hsa-miR-218-2-3p	MIMAT0004566	Target	0	2.404083395	0.0289
hsa-miR-219-000522	hsa-miR-219	hsa-miR-219-5p	MIMAT0000276	Target	0.041666667	34284460.72	0.3356
hsa-miR-219-1-3p-002095	hsa-miR-219-1-3p	hsa-miR-219-1-3p	MIMAT0004567	Target	0	1.079669797	0.8979
hsa-miR-219-2-3p-002390	hsa-miR-219-2-3p	hsa-miR-219-2-3p	MIMAT0004675	Target	0	2.404083395	0.0289
hsa-miR-22#-002301	hsa-miR-22*	hsa-miR-22-5p	MIMAT0004495	Target	0.666666667	3.44361959	0.1402
hsa-miR-22-000398	hsa-miR-22	hsa-miR-22-3p	MIMAT0000077	Target	0.25	54.91177529	0.3442
hsa-miR-220-000523	hsa-miR-220	NA	NA	Target	0.041666667	5.098124616	0.3875
hsa-miR-220b-002206	hsa-miR-220b	NA	NA	Target	0	2.404083395	0.0289
hsa-miR-220c-002211	hsa-miR-220c	NA	NA	Target	0	2.404083395	0.0289
hsa-miR-221#-002096	hsa-miR-221*	hsa-miR-221-5p	MIMAT0004568	Target	0	2.404083395	0.0289
hsa-miR-221-000524	hsa-miR-221	hsa-miR-221-3p	MIMAT0000278	Target	0.75	3.042641356	0.0877
hsa-miR-222#-002097	hsa-miR-222*	hsa-miR-222-5p	MIMAT0004569	Target	0	2.404083395	0.0289
hsa-miR-222-002276	hsa-miR-222	hsa-miR-222-3p	MIMAT0000279	Target	0.958333333	3356.31696	0.3354
hsa-miR-223#-002098	hsa-miR-223*	hsa-miR-223-5p	MIMAT0004570	Target	1	1.669847034	0.1804
hsa-miR-223-002295	hsa-miR-223	hsa-miR-223-3p	MIMAT0000280	Target	1	2.773186103	0.042
hsa-miR-224-002099	hsa-miR-224	hsa-miR-224-5p	MIMAT0000281	Target	0.666666667	2.698353392	0.0525
hsa-miR-23a#-002439	hsa-miR-23a*	hsa-miR-23a-5p	MIMAT0004496	Target	0.166666667	2.673437368	0.3382
hsa-miR-23a-000399	hsa-miR-23a	hsa-miR-23a-3p	MIMAT0000078	Target	0.291666667	0.971748498	0.971
hsa-miR-23b#-002126	hsa-miR-23b*	hsa-miR-23b-5p	MIMAT0004587	Target	0	2.404083395	0.0289
hsa-miR-23b-000400	hsa-miR-23b	hsa-miR-23b-3p	MIMAT0000418	Target	0	4.101899827	0.0864
hsa-miR-24-000402	hsa-miR-24	hsa-miR-24-3p	MIMAT0000080	Target	0.958333333	1.474214358	0.2855
hsa-miR-24-1#-002440	hsa-miR-24-1*	hsa-miR-24-1-5p	MIMAT0000079	Target	0	2.404083395	0.0289
hsa-miR-24-2#-002441	hsa-miR-24-2*	hsa-miR-24-2-5p	MIMAT0004497	Target	0	2.404083395	0.0289

hsa-miR-25#-002442	hsa-miR-25*	hsa-miR-25-5p	MIMAT0004498	Target	0.041666667	0.549892902	0.5989
hsa-miR-25-000403	hsa-miR-25	hsa-miR-25-3p	MIMAT0000081	Target	1	0.98686976	0.9594
hsa-miR-26a-000405	hsa-miR-26a	hsa-miR-26a-5p	MIMAT0000082	Target	1	1.911790225	0.1518
hsa-miR-26a-1#-002443	hsa-miR-26a-1*	hsa-miR-26a-1-3p	MIMAT0004499	Target	0	1.373991908	0.5755
hsa-miR-26a-2#-002115	hsa-miR-26a-2*	hsa-miR-26a-2-3p	MIMAT0004681	Target	0	2.404083395	0.0289
hsa-miR-26b#-002444	hsa-miR-26b*	hsa-miR-26b-3p	MIMAT0004500	Target	0.25	2.101343065	0.1696
hsa-miR-26b-000407	hsa-miR-26b	hsa-miR-26b-5p	MIMAT0000083	Target	1	2.332225079	0.0166
hsa-miR-27a#-002445	hsa-miR-27a*	hsa-miR-27a-5p	MIMAT0004501	Target	0.208333333	0.841973695	0.7935
hsa-miR-27a-000408	hsa-miR-27a	hsa-miR-27a-3p	MIMAT0000084	Target	1	1.421343862	0.4358
hsa-miR-27b#-002174	hsa-miR-27b*	hsa-miR-27b-5p	MIMAT0004588	Target	0.041666667	595.1220149	0.3351
hsa-miR-27b-000409	hsa-miR-27b	hsa-miR-27b-3p	MIMAT0000419	Target	0.875	1.367108239	0.5376
hsa-miR-28-000411	hsa-miR-28	hsa-miR-28-5p	MIMAT0000085	Target	0.666666667	0.911295075	0.8081
hsa-miR-28-3p-002446	hsa-miR-28-3p	hsa-miR-28-3p	MIMAT0004502	Target	1	1.940970878	0.097
hsa-miR-296-000527	hsa-miR-296	hsa-miR-296-5p	MIMAT0000690	Target	0.958333333	1.092158653	0.8567
hsa-miR-296-3p-002101	hsa-miR-296-3p	hsa-miR-296-3p	MIMAT0004679	Target	0	9.015235628	0.2535
hsa-miR-298-002190	hsa-miR-298	hsa-miR-298	MIMAT0004901	Target	0	2.404083395	0.0289
hsa-miR-299-3p-001015	hsa-miR-299-3p	hsa-miR-299-3p	MIMAT0000687	Target	0	2.404083395	0.0289
hsa-miR-299-5p-000600	hsa-miR-299-5p	hsa-miR-299-5p	MIMAT0002890	Target	0.041666667	1.25263E-06	0.3434
hsa-miR-29a#-002447	hsa-miR-29a*	hsa-miR-29a-5p	MIMAT0004503	Target	0.041666667	0.38122952	0.3016
hsa-miR-29a-002112	hsa-miR-29a	hsa-miR-29a-3p	MIMAT0000086	Target	1	2.023762002	0.0259
hsa-miR-29b-000413	hsa-miR-29b	hsa-miR-29b-3p	MIMAT0000100	Target	0.25	1.331861391	0.748
hsa-miR-29b-1#-002165	hsa-miR-29b-1*	hsa-miR-29b-1-5p	MIMAT0004514	Target	0	2.404083395	0.0289
hsa-miR-29b-2#-002166	hsa-miR-29b-2*	hsa-miR-29b-2-5p	MIMAT0004515	Target	0.041666667	9.552491981	0.2508
hsa-miR-29c-000587	hsa-miR-29c	hsa-miR-29c-3p	MIMAT0000681	Target	0.291666667	4.77698E-05	0.3434
hsa-miR-301-000528	hsa-miR-301	hsa-miR-301a-3p	MIMAT0000688	Target	0.833333333	1.2186446	0.6541
hsa-miR-301b-002392	hsa-miR-301b	hsa-miR-301b	MIMAT0004958	Target	0.041666667	3.547439172	0.365
hsa-miR-302a#-002381	hsa-miR-302a*	hsa-miR-302a-5p	MIMAT0000683	Target	0	2.404083395	0.0289
hsa-miR-302a-000529	hsa-miR-302a	hsa-miR-302a-3p	MIMAT0000684	Target	0.208333333	0.000372345	0.1565
hsa-miR-302b#-002119	hsa-miR-302b*	hsa-miR-302b-5p	MIMAT0000714	Target	0	2.404083395	0.0289
hsa-miR-302b-000531	hsa-miR-302b	hsa-miR-302b-3p	MIMAT0000715	Target	0.041666667	0.411701948	0.4961
hsa-miR-302c#-000534	hsa-miR-302c*	hsa-miR-302c-5p	MIMAT0000716	Target	0.041666667	154183208.2	0.3356
hsa-miR-302c-000533	hsa-miR-302c	hsa-miR-302c-3p	MIMAT0000717	Target	0.583333333	5.683733023	0.0734
hsa-miR-302d#-002120	hsa-miR-302d*	hsa-miR-302d-5p	MIMAT0004685	Target	0	2.404083395	0.0289
hsa-miR-302d-000535	hsa-miR-302d	hsa-miR-302d-3p	MIMAT0000718	Target	0.041666667	0.413551883	0.2907
hsa-miR-30a-3p-000416	hsa-miR-30a-3p	hsa-miR-30a-3p	MIMAT0000088	Target	1	1.323153482	0.4207
hsa-miR-30a-5p-000417	hsa-miR-30a-5p	hsa-miR-30a-5p	MIMAT0000087	Target	1	0.97299214	0.9152
hsa-miR-30b#-002129	hsa-miR-30b*	hsa-miR-30b-3p	MIMAT0004589	Target	0	2.404083395	0.0289
hsa-miR-30b-000602	hsa-miR-30b	hsa-miR-30b-5p	MIMAT0000420	Target	1	1.467826824	0.3386
hsa-miR-30c-000419	hsa-miR-30c	hsa-miR-30c-5p	MIMAT0000244	Target	1	0.000304878	0.1721
hsa-miR-30c-1#-002108	hsa-miR-30c-1*	hsa-miR-30c-1-3p	MIMAT0004674	Target	0	2.404083395	0.0289
hsa-miR-30c-2#-002110	hsa-miR-30c-2*	hsa-miR-30c-2-3p	MIMAT0004550	Target	0	2.404083395	0.0289
hsa-miR-30d#-002305	hsa-miR-30d*	hsa-miR-30d-3p	MIMAT0004551	Target	0.083333333	0.324066238	0.1677
hsa-miR-30d-000420	hsa-miR-30d	hsa-miR-30d-5p	MIMAT0000245	Target	1	1.249772884	0.3655
hsa-miR-30e-3p-000422	hsa-miR-30e-3p	hsa-miR-30e-3p	MIMAT0000693	Target	1	1.720731944	0.1431
hsa-miR-31#-002113	hsa-miR-31*	hsa-miR-31-3p	MIMAT0004504	Target	0.166666667	1.069982261	0.9435
hsa-miR-31-002279	hsa-miR-31	hsa-miR-31-5p	MIMAT0000089	Target	0.125	0.023011097	0.0998
hsa-miR-32#-002111	hsa-miR-32*	hsa-miR-32-3p	MIMAT0004505	Target	0	2.404083395	0.0289

hsa-miR-32-002109	hsa-miR-32	hsa-miR-32-5p	MIMAT0000090	Target	0.083333333	139895.4705	0.3356
hsa-miR-320-002277	hsa-miR-320	hsa-miR-320a	MIMAT0000510	Target	1	1.113357426	0.6779
hsa-miR-320B-002844	hsa-miR-320B	NA	NA	Target	1	0.815570325	0.4217
hsa-miR-323-3p-002227	hsa-miR-323-3p	hsa-miR-323a-3p	MIMAT0000755	Target	0.291666667	23455750.76	0.3356
hsa-miR-324-3p-002161	hsa-miR-324-3p	hsa-miR-324-3p	MIMAT0000762	Target	1	1.844550215	0.0352
hsa-miR-324-5p-000539	hsa-miR-324-5p	hsa-miR-324-5p	MIMAT0000761	Target	0.916666667	2.35644332	0.2312
hsa-miR-325-000540	hsa-miR-325	hsa-miR-325	MIMAT0000771	Target	0	2.404083395	0.0289
hsa-miR-326-000542	hsa-miR-326	hsa-miR-326	MIMAT0000756	Target	0.083333333	35.1369356	0.1702
hsa-miR-328-000543	hsa-miR-328	hsa-miR-328	MIMAT0000752	Target	1	2.643069241	0.1622
hsa-miR-329-001101	hsa-miR-329	hsa-miR-329	MIMAT0001629	Target	0.041666667	0.004674401	0.3447
hsa-miR-330-000544	hsa-miR-330	hsa-miR-330-3p	MIMAT0000751	Target	0.166666667	800.3147207	0.3361
hsa-miR-330-5p-002230	hsa-miR-330-5p	hsa-miR-330-5p	MIMAT0004693	Target	0	2.404083395	0.0289
hsa-miR-331-000545	hsa-miR-331	hsa-miR-331-3p	MIMAT0000760	Target	0.875	2.855809828	0.0463
hsa-miR-331-5p-002233	hsa-miR-331-5p	hsa-miR-331-5p	MIMAT0004700	Target	0.041666667	1681530.58	0.3356
hsa-miR-335#-002185	hsa-miR-335*	hsa-miR-335-3p	MIMAT0004703	Target	0	2.404083395	0.0289
hsa-miR-335-000546	hsa-miR-335	hsa-miR-335-5p	MIMAT0000765	Target	1	1.345035348	0.3792
hsa-miR-337-3p-002157	hsa-miR-337-3p	hsa-miR-337-3p	MIMAT0000754	Target	0	2.404083395	0.0289
hsa-miR-337-5p-002156	hsa-miR-337-5p	hsa-miR-337-5p	MIMAT0004695	Target	0.041666667	0.782375893	0.7538
hsa-miR-338-3p-002252	hsa-miR-338-3p	hsa-miR-338-3p	MIMAT0000763	Target	0	2.678186853	0.4487
hsa-miR-338-5P-002658	hsa-miR-338-5P	NA	NA	Target	1	1.336112944	0.5194
hsa-miR-339-3p-002184	hsa-miR-339-3p	hsa-miR-339-3p	MIMAT0004702	Target	0.791666667	1.016343312	0.9668
hsa-miR-339-5p-002257	hsa-miR-339-5p	hsa-miR-339-5p	MIMAT0000764	Target	0	2.404083395	0.0289
hsa-miR-33a#-002136	hsa-miR-33a*	hsa-miR-33a-3p	MIMAT0004506	Target	0.041666667	110.4752652	0.3311
hsa-miR-33a-002135	hsa-miR-33a	hsa-miR-33a-5p	MIMAT0000091	Target	0	2.404083395	0.0289
hsa-miR-33b-002085	hsa-miR-33b	hsa-miR-33b-5p	MIMAT0003301	Target	0	2.404083395	0.0289
hsa-miR-340#-002259	hsa-miR-340*	hsa-miR-340-3p	MIMAT0000750	Target	0.208333333	0.795214735	0.7295
hsa-miR-340-002258	hsa-miR-340	hsa-miR-340-5p	MIMAT0004692	Target	0.375	1.920536819	0.3473
hsa-miR-342-3p-002260	hsa-miR-342-3p	hsa-miR-342-3p	MIMAT0000753	Target	0.916666667	1.54788304	0.3466
hsa-miR-342-5p-002147	hsa-miR-342-5p	hsa-miR-342-5p	MIMAT0004694	Target	0	2.404083395	0.0289
hsa-miR-345-002186	hsa-miR-345	hsa-miR-345-5p	MIMAT0000772	Target	1	1.536943007	0.2938
hsa-miR-346-000553	hsa-miR-346	hsa-miR-346	MIMAT0000773	Target	0	2.404083395	0.0289
hsa-miR-34a#-002316	hsa-miR-34a*	hsa-miR-34a-3p	MIMAT0004557	Target	0.916666667	1.54593675	0.319
hsa-miR-34a-000426	hsa-miR-34a	hsa-miR-34a-5p	MIMAT0000255	Target	0.541666667	22.99376212	0.189
hsa-miR-34b-000427	hsa-miR-34b	hsa-miR-34b-5p	MIMAT0000685	Target	0.041666667	6.106587713	0.1788
hsa-miR-34b-002102	hsa-miR-34b	hsa-miR-34b-3p	MIMAT0004676	Target	0.625	2.718756758	0.1884
hsa-miR-34c-000428	hsa-miR-34c	hsa-miR-34c-5p	MIMAT0000686	Target	0.041666667	883748731.2	0.3356
hsa-miR-361-000554	hsa-miR-361	hsa-miR-361-5p	MIMAT0000703	Target	0.75	1.675657205	0.3364
hsa-miR-361-3p-002116	hsa-miR-361-3p	hsa-miR-361-3p	MIMAT0004682	Target	0	2.404083395	0.0289
hsa-miR-362-001273	hsa-miR-362	hsa-miR-362-5p	MIMAT0000705	Target	0.208333333	4.856536197	0.0776
hsa-miR-362-3p-002117	hsa-miR-362-3p	hsa-miR-362-3p	MIMAT0004683	Target	0.166666667	2.288710806	0.2852
hsa-miR-363#-001283	hsa-miR-363*	hsa-miR-363-5p	MIMAT0003385	Target	0	2.404083395	0.0289
hsa-miR-363-001271	hsa-miR-363	hsa-miR-363-3p	MIMAT0000707	Target	0.291666667	0.892134511	0.8927
hsa-miR-365-001020	hsa-miR-365	hsa-miR-365a-3p	MIMAT0000710	Target	0.791666667	4.076677668	0.0641
hsa-miR-367#-002121	hsa-miR-367*	hsa-miR-367-5p	MIMAT0004686	Target	0	2.404083395	0.0289
hsa-miR-367-000555	hsa-miR-367	hsa-miR-367-3p	MIMAT0000719	Target	0.333333333	1.432851919	0.6617
hsa-miR-369-3p-000557	hsa-miR-369-3p	hsa-miR-369-3p	MIMAT0000721	Target	0	2.404083395	0.0289
hsa-miR-369-5p-001021	hsa-miR-369-5p	hsa-miR-369-5p	MIMAT0001621	Target	0	2.404083395	0.0289

hsa-miR-370-002275	hsa-miR-370	hsa-miR-370	MIMAT0000722	Target	0	2.404083395	0.0289
hsa-miR-371-3p-002124	hsa-miR-371-3p	hsa-miR-371a-3p	MIMAT0000723	Target	0	2.404083395	0.0289
hsa-miR-372-000560	hsa-miR-372	hsa-miR-372	MIMAT0000724	Target	0.208333333	0.029382671	0.3282
hsa-miR-373-000561	hsa-miR-373	hsa-miR-373-3p	MIMAT0000726	Target	0	8.860103627	0.27
hsa-miR-374-000563	hsa-miR-374	hsa-miR-374a-5p	MIMAT0000727	Target	0.958333333	1.465298351	0.3769
hsa-miR-374a#-002125	hsa-miR-374a*	hsa-miR-374a-3p	MIMAT0004688	Target	0	2.404083395	0.0289
hsa-miR-374b#-002391	hsa-miR-374b*	hsa-miR-374b-3p	MIMAT0004956	Target	0.041666667	0.011726068	0.3472
hsa-miR-375-000564	hsa-miR-375	hsa-miR-375	MIMAT0000728	Target	0.958333333	3.496423711	0.0416
hsa-miR-376a#-002127	hsa-miR-376a*	hsa-miR-376a-5p	MIMAT0003386	Target	0	2.404083395	0.0289
hsa-miR-376a-000565	hsa-miR-376a	hsa-miR-376a-3p	MIMAT0000729	Target	0.625	0.963365976	0.9553
hsa-miR-376b-001102	hsa-miR-376b	hsa-miR-376b	MIMAT0002172	Target	0.458333333	1.812873226	0.3795
hsa-miR-376c-002122	hsa-miR-376c	hsa-miR-376c	MIMAT0000720	Target	0.708333333	2.074032598	0.1587
hsa-miR-377#-002128	hsa-miR-377*	hsa-miR-377-5p	MIMAT0004689	Target	0	2.404083395	0.0289
hsa-miR-377-000566	hsa-miR-377	hsa-miR-377-3p	MIMAT0000730	Target	0.375	4.719448732	0.1762
hsa-miR-378-000567	hsa-miR-378	hsa-miR-378a-5p	MIMAT0000731	Target	0.583333333	1.11184316	0.8542
hsa-miR-378-002243	hsa-miR-378	hsa-miR-378a-3p	MIMAT0000732	Target	1	1.140697118	0.742
hsa-miR-380-3p-000569	hsa-miR-380-3p	hsa-miR-380-3p	MIMAT0000735	Target	0	2.404083395	0.0289
hsa-miR-380-5p-000570	hsa-miR-380-5p	hsa-miR-380-5p	MIMAT0000734	Target	0.041666667	2.39987E-08	0.3434
hsa-miR-381-000571	hsa-miR-381	hsa-miR-381	MIMAT0000736	Target	0.541666667	0.010293605	0.3478
hsa-miR-382-000572	hsa-miR-382	hsa-miR-382-5p	MIMAT0000737	Target	0	2.404083395	0.0289
hsa-miR-383-000573	hsa-miR-383	hsa-miR-383	MIMAT0000738	Target	0	2.404083395	0.0289
hsa-miR-384-000574	hsa-miR-384	hsa-miR-384	MIMAT0001075	Target	0.041666667	0.389851361	0.4817
hsa-miR-409-3p-002332	hsa-miR-409-3p	hsa-miR-409-3p	MIMAT0001639	Target	0.541666667	32.24163041	0.3062
hsa-miR-409-5p-002331	hsa-miR-409-5p	hsa-miR-409-5p	MIMAT0001638	Target	0	2.404083395	0.0289
hsa-miR-410-001274	hsa-miR-410	hsa-miR-410	MIMAT0002171	Target	0.416666667	1.882825383	0.2819
hsa-miR-411#-002238	hsa-miR-411*	hsa-miR-411-3p	MIMAT0004813	Target	0	2.404083395	0.0289
hsa-miR-411-001610	hsa-miR-411	hsa-miR-411-5p	MIMAT0003329	Target	0.125	0.779075365	0.7676
hsa-miR-412-001023	hsa-miR-412	hsa-miR-412	MIMAT0002170	Target	0	2.404083395	0.0289
hsa-miR-422a-002297	hsa-miR-422a	hsa-miR-422a	MIMAT0001339	Target	0.541666667	1.8407764	0.2863
hsa-miR-423-5p-002340	hsa-miR-423-5p	hsa-miR-423-5p	MIMAT0004748	Target	0.958333333	0.633467723	0.3157
hsa-miR-424#-002309	hsa-miR-424*	hsa-miR-424-3p	MIMAT0004749	Target	0	6.640759931	0.2169
hsa-miR-424-000604	hsa-miR-424	hsa-miR-424-5p	MIMAT0001341	Target	0.083333333	5.680452969	0.3983
hsa-miR-425#-002302	hsa-miR-425*	hsa-miR-425-3p	MIMAT0001343	Target	0.333333333	12.47570303	0.1072
hsa-miR-425-5p-001516	hsa-miR-425-5p	hsa-miR-425-5p	MIMAT0003393	Target	0.541666667	0.658100258	0.6138
hsa-miR-429-001024	hsa-miR-429	hsa-miR-429	MIMAT0001536	Target	0.041666667	3.89703921	0.1964
hsa-miR-431#-002312	hsa-miR-431*	hsa-miR-431-3p	MIMAT0004757	Target	0	2.404083395	0.0289
hsa-miR-431-001979	hsa-miR-431	hsa-miR-431-5p	MIMAT0001625	Target	0.041666667	134.0497779	0.3304
hsa-miR-432#-001027	hsa-miR-432*	hsa-miR-432-3p	MIMAT0002815	Target	0.041666667	641602000.1	0.3356
hsa-miR-432-001026	hsa-miR-432	hsa-miR-432-5p	MIMAT0002814	Target	0.166666667	973.409006	0.3353
hsa-miR-433-001028	hsa-miR-433	hsa-miR-433	MIMAT0001627	Target	0	1.805986748	0.1201
hsa-miR-448-001029	hsa-miR-448	hsa-miR-448	MIMAT0001532	Target	0	2.404083395	0.0289
hsa-miR-449-001030	hsa-miR-449	hsa-miR-449a	MIMAT0001541	Target	0	2.404083395	0.0289
hsa-miR-449b-001608	hsa-miR-449b	hsa-miR-449b-5p	MIMAT0003327	Target	0	0.638369312	0.649
hsa-miR-450a-002303	hsa-miR-450a	hsa-miR-450a-5p	MIMAT0001545	Target	0	2.404083395	0.0289
hsa-miR-450b-3p-002208	hsa-miR-450b-3p	hsa-miR-450b-3p	MIMAT0004910	Target	0	2.404083395	0.0289
hsa-miR-450b-5p-002207	hsa-miR-450b-5p	hsa-miR-450b-5p	MIMAT0004909	Target	0	2.404083395	0.0289
hsa-miR-452#-002330	hsa-miR-452*	hsa-miR-452-3p	MIMAT0001636	Target	0	2.404083395	0.0289

hsa-miR-452-002329	hsa-miR-452	hsa-miR-452-5p	MIMAT0001635	Target	0.166666667	620458.3353	0.3356
hsa-miR-453-002318	hsa-miR-453	hsa-miR-323b-5p	MIMAT0001630	Target	0	2.404083395	0.0289
hsa-miR-454#-001996	hsa-miR-454*	hsa-miR-454-5p	MIMAT0003884	Target	0	3.73334567	0.0008
hsa-miR-454-002323	hsa-miR-454	hsa-miR-454-3p	MIMAT0003885	Target	0.833333333	1.515045532	0.3641
hsa-miR-455-001280	hsa-miR-455	hsa-miR-455-5p	MIMAT0003150	Target	0.166666667	2.726767804	0.224
hsa-miR-455-3p-002244	hsa-miR-455-3p	hsa-miR-455-3p	MIMAT0004784	Target	0.083333333	63.34536146	0.3095
hsa-miR-483-3p-002339	hsa-miR-483-3p	hsa-miR-483-3p	MIMAT0002173	Target	0.083333333	2044.477239	0.3043
hsa-miR-483-5p-002338	hsa-miR-483-5p	hsa-miR-483-5p	MIMAT0004761	Target	1	4.094370778	0.1586
hsa-miR-484-001821	hsa-miR-484	hsa-miR-484	MIMAT0002174	Target	1	2.505336297	0.0062
hsa-miR-485-3p-001277	hsa-miR-485-3p	hsa-miR-485-3p	MIMAT0002176	Target	0.25	0.597843763	0.687
hsa-miR-485-5p-001036	hsa-miR-485-5p	hsa-miR-485-5p	MIMAT0002175	Target	0	2.404083395	0.0289
hsa-miR-486-001278	hsa-miR-486	hsa-miR-486-5p	MIMAT0002177	Target	1	1.111496364	0.5733
hsa-miR-486-3p-002093	hsa-miR-486-3p	hsa-miR-486-3p	MIMAT0004762	Target	0.75	0.736594601	0.5852
hsa-miR-487a-001279	hsa-miR-487a	hsa-miR-487a	MIMAT0002178	Target	0	2.404083395	0.0289
hsa-miR-487b-001285	hsa-miR-487b	hsa-miR-487b	MIMAT0003180	Target	0	1.34262291	0.7198
hsa-miR-488-001106	hsa-miR-488	hsa-miR-488-5p	MIMAT0002804	Target	0.125	1.425312254	0.7811
hsa-miR-488-002357	hsa-miR-488	hsa-miR-488-3p	MIMAT0004763	Target	0.041666667	0.019786094	0.3504
hsa-miR-489-002358	hsa-miR-489	hsa-miR-489	MIMAT0002805	Target	0.166666667	2.070333327	0.3169
hsa-miR-490-001037	hsa-miR-490	hsa-miR-490-3p	MIMAT0002806	Target	0.083333333	0.093360682	0.3898
hsa-miR-491-3p-002360	hsa-miR-491-3p	hsa-miR-491-3p	MIMAT0004765	Target	0	2.404083395	0.0289
hsa-miR-492-001039	hsa-miR-492	hsa-miR-492	MIMAT0002812	Target	0	2.404083395	0.0289
hsa-miR-493-002364	hsa-miR-493	hsa-miR-493-3p	MIMAT0003161	Target	0	2.404083395	0.0289
hsa-miR-494-002365	hsa-miR-494	hsa-miR-494	MIMAT0002816	Target	0	0.887897861	0.8422
hsa-miR-497#-002368	hsa-miR-497*	hsa-miR-497-3p	MIMAT0004768	Target	0	2.404083395	0.0289
hsa-miR-497-001043	hsa-miR-497	hsa-miR-497-5p	MIMAT0002820	Target	0.125	0.570619585	0.5366
hsa-miR-499-3p-002427	hsa-miR-499-3p	hsa-miR-499a-3p	MIMAT0004772	Target	0	2.404083395	0.0289
hsa-miR-500-001046	hsa-miR-500	hsa-miR-500a-3p	MIMAT0002871	Target	0.083333333	5.12182E-08	0.2938
hsa-miR-500-002428	hsa-miR-500	hsa-miR-500a-5p	MIMAT0004773	Target	0.25	6.74659559	0.2495
hsa-miR-501-001047	hsa-miR-501	hsa-miR-501-5p	MIMAT0002872	Target	0	2.404083395	0.0289
hsa-miR-501-3p-002435	hsa-miR-501-3p	hsa-miR-501-3p	MIMAT0004774	Target	0.083333333	0.097357528	0.3794
hsa-miR-502-001109	hsa-miR-502	hsa-miR-502-5p	MIMAT0002873	Target	0.291666667	0.90429458	0.9124
hsa-miR-502-3p-002083	hsa-miR-502-3p	hsa-miR-502-3p	MIMAT0004775	Target	0.25	1.649508648	0.2547
hsa-miR-503-001048	hsa-miR-503	hsa-miR-503	MIMAT0002874	Target	0	2.404083395	0.0289
hsa-miR-504-002084	hsa-miR-504	hsa-miR-504	MIMAT0002875	Target	0	2.404083395	0.0289
hsa-miR-505#-002087	hsa-miR-505*	hsa-miR-505-5p	MIMAT0004776	Target	0.833333333	0.992780528	0.9834
hsa-miR-505-002089	hsa-miR-505	hsa-miR-505-3p	MIMAT0002876	Target	0.125	5.659161638	0.3852
hsa-miR-506-001050	hsa-miR-506	hsa-miR-506-3p	MIMAT0002878	Target	0	2.404083395	0.0289
hsa-miR-507-001051	hsa-miR-507	hsa-miR-507	MIMAT0002879	Target	0	2.404083395	0.0289
hsa-miR-508-001052	hsa-miR-508	hsa-miR-508-3p	MIMAT0002880	Target	0	2.404083395	0.0289
hsa-miR-508-5p-002092	hsa-miR-508-5p	hsa-miR-508-5p	MIMAT0004778	Target	0	2.404083395	0.0289
hsa-miR-509-3-5p-002155	hsa-miR-509-3-5p	hsa-miR-509-3-5p	MIMAT0004975	Target	0	2.404083395	0.0289
hsa-miR-509-5p-002235	hsa-miR-509-5p	hsa-miR-509-5p	MIMAT0004779	Target	0.125	0.001496118	0.344
hsa-miR-510-002241	hsa-miR-510	hsa-miR-510	MIMAT0002882	Target	0	2.404083395	0.0289
hsa-miR-511-001111	hsa-miR-511	hsa-miR-511	MIMAT0002808	Target	0.375	0.697634916	0.4939
hsa-miR-512-3p-001823	hsa-miR-512-3p	hsa-miR-512-3p	MIMAT0002823	Target	0	2.404083395	0.0289
hsa-miR-512-5p-001145	hsa-miR-512-5p	hsa-miR-512-5p	MIMAT0002822	Target	0	2.404083395	0.0289
hsa-miR-513-5p-002090	hsa-miR-513-5p	hsa-miR-513a-5p	MIMAT0002877	Target	0	2.404083395	0.0289

hsa-miR-513B-002757	hsa-miR-513B	NA	NA	Target	0	2.404083395	0.0289
hsa-miR-513C-002756	hsa-miR-513C	NA	NA	Target	0	2.404083395	0.0289
hsa-miR-515-3p-002369	hsa-miR-515-3p	hsa-miR-515-3p	MIMAT0002827	Target	0.458333333	32.53036932	0.0553
hsa-miR-515-5p-001112	hsa-miR-515-5p	hsa-miR-515-5p	MIMAT0002826	Target	0	1.055517942	0.9297
hsa-miR-516-3p-001149	hsa-miR-516-3p	hsa-miR-516b-3p	MIMAT0002860	Target	0.041666667	5.27281746	0.1073
hsa-miR-516a-5p-002416	hsa-miR-516a-5p	hsa-miR-516a-5p	MIMAT0004770	Target	0	2.404083395	0.0289
hsa-miR-516b-001150	hsa-miR-516b	hsa-miR-516b-5p	MIMAT0002859	Target	0	2.404083395	0.0289
hsa-miR-517#-001113	hsa-miR-517*	hsa-miR-517-5p	MIMAT0002851	Target	0	2.404083395	0.0289
hsa-miR-517a-002402	hsa-miR-517a	hsa-miR-517a-3p	MIMAT0002852	Target	0	2.358815512	0.3694
hsa-miR-517b-001152	hsa-miR-517b	hsa-miR-517b-3p	MIMAT0002857	Target	0	2.404083395	0.0289
hsa-miR-517c-001153	hsa-miR-517c	hsa-miR-517c-3p	MIMAT0002866	Target	0.041666667	4.268219894	0.0773
hsa-miR-518a-3p-002397	hsa-miR-518a-3p	hsa-miR-518a-3p	MIMAT0002863	Target	0	2.404083395	0.0289
hsa-miR-518a-5p-002396	hsa-miR-518a-5p	hsa-miR-518a-5p	MIMAT0005457	Target	0	2.404083395	0.0289
hsa-miR-518b-001156	hsa-miR-518b	hsa-miR-518b	MIMAT0002844	Target	0.166666667	3.03314161	0.5222
hsa-miR-518c#-001158	hsa-miR-518c*	hsa-miR-518c-5p	MIMAT0002847	Target	0	2.404083395	0.0289
hsa-miR-518c-002401	hsa-miR-518c	hsa-miR-518c-3p	MIMAT0002848	Target	0	2.404083395	0.0289
hsa-miR-518d-001159	hsa-miR-518d	hsa-miR-518d-3p	MIMAT0002864	Target	0.25	3.823121456	0.2994
hsa-miR-518d-5p-002389	hsa-miR-518d-5p	hsa-miR-518d-5p	MIMAT0005456	Target	0	2.404083395	0.0289
hsa-miR-518e#-002371	hsa-miR-518e*	hsa-miR-518e-5p	MIMAT0005450	Target	0	2.404083395	0.0289
hsa-miR-518e-002395	hsa-miR-518e	hsa-miR-518e-3p	MIMAT0002861	Target	0	3.64493689	0.1975
hsa-miR-518f#-002387	hsa-miR-518f*	hsa-miR-518f-5p	MIMAT0002841	Target	0	2.404083395	0.0289
hsa-miR-518f-002388	hsa-miR-518f	hsa-miR-518f-3p	MIMAT0002842	Target	0.208333333	5.989919269	0.2289
hsa-miR-519a-002415	hsa-miR-519a	hsa-miR-519a-3p	MIMAT0002869	Target	0	2.547858656	0.2287
hsa-miR-519b-3p-002384	hsa-miR-519b-3p	hsa-miR-519b-3p	MIMAT0002837	Target	0.041666667	6.829337495	0.2211
hsa-miR-519c-001163	hsa-miR-519c	hsa-miR-519c-3p	MIMAT0002832	Target	0	2.101078167	0.0459
hsa-miR-519d-002403	hsa-miR-519d	hsa-miR-519d	MIMAT0002853	Target	0	2.483749819	0.0103
hsa-miR-519e#-001166	hsa-miR-519e*	hsa-miR-519e-5p	MIMAT0002828	Target	0	2.404083395	0.0289
hsa-miR-519e-002370	hsa-miR-519e	hsa-miR-519e-3p	MIMAT0002829	Target	0	2.404083395	0.0289
hsa-miR-520a#-001168	hsa-miR-520a*	hsa-miR-520a-5p	MIMAT0002833	Target	0	2.404083395	0.0289
hsa-miR-520a-001167	hsa-miR-520a	hsa-miR-520a-3p	MIMAT0002834	Target	0	1.499961515	0.3226
hsa-miR-520b-001116	hsa-miR-520b	hsa-miR-520b	MIMAT0002843	Target	0.25	1.51229859	0.6246
hsa-miR-520c-3p-002400	hsa-miR-520c-3p	hsa-miR-520c-3p	MIMAT0002846	Target	0.25	4.218252061	0.4457
hsa-miR-520D-3P-002743	hsa-miR-520D-3P	NA	NA	Target	0.333333333	0.135208592	0.1917
hsa-miR-520d-5p-002393	hsa-miR-520d-5p	hsa-miR-520d-5p	MIMAT0002855	Target	0.041666667	0.031258847	0.3519
hsa-miR-520e-001119	hsa-miR-520e	hsa-miR-520e	MIMAT0002825	Target	0.291666667	0.195550239	0.3884
hsa-miR-520f-001120	hsa-miR-520f	hsa-miR-520f	MIMAT0002830	Target	0	2.404083395	0.0289
hsa-miR-520g-001121	hsa-miR-520g	hsa-miR-520g	MIMAT0002858	Target	0.041666667	959.0152356	0.3348
hsa-miR-520h-001170	hsa-miR-520h	hsa-miR-520h	MIMAT0002867	Target	0	2.404083395	0.0289
hsa-miR-521-001122	hsa-miR-521	hsa-miR-521	MIMAT0002854	Target	0	1.761939312	0.4248
hsa-miR-522-002413	hsa-miR-522	hsa-miR-522-3p	MIMAT0002868	Target	0	2.404083395	0.0289
hsa-miR-523-002386	hsa-miR-523	hsa-miR-523-3p	MIMAT0002840	Target	0.375	2.445765873	0.4435
hsa-miR-524-001173	hsa-miR-524	hsa-miR-524-3p	MIMAT0002850	Target	0.416666667	3.441010282	0.1762
hsa-miR-524-5p-001982	hsa-miR-524-5p	hsa-miR-524-5p	MIMAT0002849	Target	0	2.404083395	0.0289
hsa-miR-525-001174	hsa-miR-525	hsa-miR-525-5p	MIMAT0002838	Target	0	2.404083395	0.0289
hsa-miR-525-3p-002385	hsa-miR-525-3p	hsa-miR-525-3p	MIMAT0002839	Target	0	2.404083395	0.0289
hsa-miR-526b-002382	hsa-miR-526b	hsa-miR-526b-5p	MIMAT0002835	Target	0	2.404083395	0.0289
hsa-miR-532-001518	hsa-miR-532	hsa-miR-532-5p	MIMAT0002888	Target	0.916666667	1.254526185	0.5833

hsa-miR-532-3p-002355	hsa-miR-532-3p	hsa-miR-532-3p	MIMAT0004780	Target	0.583333333	3.18509E-05	0.3434
hsa-miR-539-001286	hsa-miR-539	hsa-miR-539-5p	MIMAT0003163	Target	0.125	48.96732898	0.3188
hsa-miR-541#-002200	hsa-miR-541*	hsa-miR-541-5p	MIMAT0004919	Target	0	2.404083395	0.0289
hsa-miR-541-002201	hsa-miR-541	hsa-miR-541-3p	MIMAT0004920	Target	0	2.404083395	0.0289
hsa-miR-542-3p-001284	hsa-miR-542-3p	hsa-miR-542-3p	MIMAT0003389	Target	0	2.404083395	0.0289
hsa-miR-542-5p-002240	hsa-miR-542-5p	hsa-miR-542-5p	MIMAT0003340	Target	0	2.404083395	0.0289
hsa-miR-543-002376	hsa-miR-543	hsa-miR-543	MIMAT0004954	Target	0.083333333	0.199165014	0.2184
hsa-miR-544-002265	hsa-miR-544	hsa-miR-544a	MIMAT0003164	Target	0	2.404083395	0.0289
hsa-miR-545#-002266	hsa-miR-545*	hsa-miR-545-5p	MIMAT0004785	Target	0	3.419380706	0.0195
hsa-miR-545-002267	hsa-miR-545	hsa-miR-545-3p	MIMAT0003165	Target	0.083333333	1.841166949	0.4169
hsa-miR-548a-001538	hsa-miR-548a	hsa-miR-548a-3p	MIMAT0003251	Target	0.208333333	5.157200091	0.0853
hsa-miR-548a-5p-002412	hsa-miR-548a-5p	hsa-miR-548a-5p	MIMAT0004803	Target	0	2.404083395	0.0289
hsa-miR-548b-001541	hsa-miR-548b	hsa-miR-548b-3p	MIMAT0003254	Target	0	2.404083395	0.0289
hsa-miR-548b-5p-002408	hsa-miR-548b-5p	hsa-miR-548b-5p	MIMAT0004798	Target	0.208333333	0.762335966	0.7162
hsa-miR-548c-001590	hsa-miR-548c	hsa-miR-548c-3p	MIMAT0003285	Target	0.166666667	3.786638159	0.0714
hsa-miR-548c-5p-002429	hsa-miR-548c-5p	hsa-miR-548c-5p	MIMAT0004806	Target	0.208333333	1.078535607	0.903
hsa-miR-548d-001605	hsa-miR-548d	hsa-miR-548d-3p	MIMAT0003323	Target	0.041666667	18.16894893	0.2926
hsa-miR-548d-5p-002237	hsa-miR-548d-5p	hsa-miR-548d-5p	MIMAT0004812	Target	0.208333333	2.741105239	0.3373
hsa-miR-548E-002881	hsa-miR-548E	NA	NA	Target	0	2.404083395	0.0289
hsa-miR-548G-002879	hsa-miR-548G	NA	NA	Target	0	2.404083395	0.0289
hsa-miR-548H-002816	hsa-miR-548H	NA	NA	Target	0	1.502389947	0.3422
hsa-miR-548I-002909	hsa-miR-548I	NA	NA	Target	0	8.239884037	0.2591
hsa-miR-548J-002783	hsa-miR-548J	NA	NA	Target	0	1.508495568	0.3138
hsa-miR-548K-002819	hsa-miR-548K	NA	NA	Target	0	2.404083395	0.0289
hsa-miR-548L-002904	hsa-miR-548L	NA	NA	Target	0	8.989328892	0.2665
hsa-miR-548M-002775	hsa-miR-548M	NA	NA	Target	0	4.34215396	0.1337
hsa-miR-548N-002888	hsa-miR-548N	NA	NA	Target	0	2.404083395	0.0289
hsa-miR-548P-002798	hsa-miR-548P	NA	NA	Target	0	2.404083395	0.0289
hsa-miR-549-001511	hsa-miR-549	hsa-miR-549	MIMAT0003333	Target	0	2.404083395	0.0289
hsa-miR-550-001544	hsa-miR-550	hsa-miR-550a-3p	MIMAT0003257	Target	0	1.005391322	0.9939
hsa-miR-550-002410	hsa-miR-550	hsa-miR-550a-5p	MIMAT0004800	Target	0.083333333	1.438779638	0.5902
hsa-miR-551a-001519	hsa-miR-551a	hsa-miR-551a	MIMAT0003214	Target	0.041666667	0.216698729	0.3484
hsa-miR-551b#-002346	hsa-miR-551b*	hsa-miR-551b-5p	MIMAT0004794	Target	0.5	1.970292651	0.2303
hsa-miR-551b-001535	hsa-miR-551b	hsa-miR-551b-3p	MIMAT0003233	Target	0	2.404083395	0.0289
hsa-miR-552-001520	hsa-miR-552	hsa-miR-552	MIMAT0003215	Target	0	2.404083395	0.0289
hsa-miR-553-001521	hsa-miR-553	hsa-miR-553	MIMAT0003216	Target	0	2.404083395	0.0289
hsa-miR-554-001522	hsa-miR-554	hsa-miR-554	MIMAT0003217	Target	0	2.404083395	0.0289
hsa-miR-555-001523	hsa-miR-555	hsa-miR-555	MIMAT0003219	Target	0	2.404083395	0.0289
hsa-miR-556-3p-002345	hsa-miR-556-3p	hsa-miR-556-3p	MIMAT0004793	Target	0	2.404083395	0.0289
hsa-miR-556-5p-002344	hsa-miR-556-5p	hsa-miR-556-5p	MIMAT0003220	Target	0	2.404083395	0.0289
hsa-miR-557-001525	hsa-miR-557	hsa-miR-557	MIMAT0003221	Target	0.083333333	9.41824E-10	0.3434
hsa-miR-558-001526	hsa-miR-558	hsa-miR-558	MIMAT0003222	Target	0	2.404083395	0.0289
hsa-miR-559-001527	hsa-miR-559	hsa-miR-559	MIMAT0003223	Target	0	0.832016907	0.8159
hsa-miR-561-001528	hsa-miR-561	hsa-miR-561-3p	MIMAT0003225	Target	0.041666667	71779395.7	0.3356
hsa-miR-562-001529	hsa-miR-562	hsa-miR-562	MIMAT0003226	Target	0	2.404083395	0.0289
hsa-miR-563-001530	hsa-miR-563	hsa-miR-563	MIMAT0003227	Target	0	2.404083395	0.0289
hsa-miR-564-001531	hsa-miR-564	hsa-miR-564	MIMAT0003228	Target	0.083333333	0.017152046	0.3512

hsa-miR-566-001533	hsa-miR-566	hsa-miR-566	MIMAT0003230	Target	0.083333333	290471514	0.3356
hsa-miR-567-001534	hsa-miR-567	hsa-miR-567	MIMAT0003231	Target	0.083333333	0.088808637	0.378
hsa-miR-569-001536	hsa-miR-569	hsa-miR-569	MIMAT0003234	Target	0.041666667	0.07982362	0.3658
hsa-miR-570-002347	hsa-miR-570	hsa-miR-570-3p	MIMAT0003235	Target	0.083333333	0.366504447	0.5635
hsa-miR-571-001613	hsa-miR-571	hsa-miR-571	MIMAT0003236	Target	0.041666667	7.465457686	0.2216
hsa-miR-572-001614	hsa-miR-572	hsa-miR-572	MIMAT0003237	Target	0.625	3.843008905	0.2797
hsa-miR-573-001615	hsa-miR-573	hsa-miR-573	MIMAT0003238	Target	0.166666667	28.02790219	0.1292
hsa-miR-574-3p-002349	hsa-miR-574-3p	hsa-miR-574-3p	MIMAT0003239	Target	1	3.153933475	0.0085
hsa-miR-575-001617	hsa-miR-575	hsa-miR-575	MIMAT0003240	Target	0.041666667	5.90022E-08	0.3434
hsa-miR-576-3p-002351	hsa-miR-576-3p	hsa-miR-576-3p	MIMAT0004796	Target	0.291666667	0.948007875	0.921
hsa-miR-576-5p-002350	hsa-miR-576-5p	hsa-miR-576-5p	MIMAT0003241	Target	0.041666667	3.887372098	0.3442
hsa-miR-577-002675	hsa-miR-577	hsa-miR-577	MIMAT0003242	Target	0.041666667	1.063176846	0.9153
hsa-miR-578-001619	hsa-miR-578	hsa-miR-578	MIMAT0003243	Target	0	2.404083395	0.0289
hsa-miR-579-002398	hsa-miR-579	hsa-miR-579	MIMAT0003244	Target	0.666666667	0.287736719	0.0534
hsa-miR-580-001621	hsa-miR-580	hsa-miR-580	MIMAT0003245	Target	0	2.404083395	0.0289
hsa-miR-581-001622	hsa-miR-581	hsa-miR-581	MIMAT0003246	Target	0	2.404083395	0.0289
hsa-miR-582-3p-002399	hsa-miR-582-3p	hsa-miR-582-3p	MIMAT0004797	Target	0	5.06044905	0.15
hsa-miR-582-5p-001983	hsa-miR-582-5p	hsa-miR-582-5p	MIMAT0003247	Target	0	2.404083395	0.0289
hsa-miR-583-001623	hsa-miR-583	hsa-miR-583	MIMAT0003248	Target	0	2.404083395	0.0289
hsa-miR-584-001624	hsa-miR-584	hsa-miR-584-5p	MIMAT0003249	Target	0.041666667	28.72070072	0.3127
hsa-miR-585-001625	hsa-miR-585	hsa-miR-585	MIMAT0003250	Target	0	2.404083395	0.0289
hsa-miR-586-001539	hsa-miR-586	hsa-miR-586	MIMAT0003252	Target	0.041666667	7984207951	0.3356
hsa-miR-587-001540	hsa-miR-587	hsa-miR-587	MIMAT0003253	Target	0.125	29078892.17	0.3356
hsa-miR-588-001542	hsa-miR-588	hsa-miR-588	MIMAT0003255	Target	0.083333333	0.181065262	0.4371
hsa-miR-589-001543	hsa-miR-589	hsa-miR-589-3p	MIMAT0003256	Target	0	2.404083395	0.0289
hsa-miR-589-002409	hsa-miR-589	hsa-miR-589-5p	MIMAT0004799	Target	0	2.809670584	0.3466
hsa-miR-590-3P-002677	hsa-miR-590-3p	NA	NA	Target	0.166666667	1.900089676	0.1523
hsa-miR-590-5p-001984	hsa-miR-590-5p	hsa-miR-590-5p	MIMAT0003258	Target	1	1.084311957	0.8001
hsa-miR-591-001545	hsa-miR-591	hsa-miR-591	MIMAT0003259	Target	0	2.729631869	0.0461
hsa-miR-592-001546	hsa-miR-592	hsa-miR-592	MIMAT0003260	Target	0.041666667	0.001329268	0.344
hsa-miR-593-001547	hsa-miR-593	hsa-miR-593-5p	MIMAT0003261	Target	0.041666667	7.99852E-08	0.3434
hsa-miR-593-002411	hsa-miR-593	hsa-miR-593-3p	MIMAT0004802	Target	0	2.404083395	0.0289
hsa-miR-595-001987	hsa-miR-595	hsa-miR-595	MIMAT0003263	Target	0.041666667	6.17878E-10	0.3434
hsa-miR-596-001550	hsa-miR-596	hsa-miR-596	MIMAT0003264	Target	0.041666667	0.000947754	0.3437
hsa-miR-597-001551	hsa-miR-597	hsa-miR-597	MIMAT0003265	Target	0.958333333	2.700830272	0.0837
hsa-miR-598-001988	hsa-miR-598	hsa-miR-598	MIMAT0003266	Target	0.833333333	175.7698021	0.2546
hsa-miR-599-001554	hsa-miR-599	hsa-miR-599	MIMAT0003267	Target	0	1.108472455	0.8663
hsa-miR-600-001556	hsa-miR-600	hsa-miR-600	MIMAT0003268	Target	0	2.404083395	0.0289
hsa-miR-601-001558	hsa-miR-601	hsa-miR-601	MIMAT0003269	Target	0.75	1.613642699	0.4657
hsa-miR-603-001566	hsa-miR-603	hsa-miR-603	MIMAT0003271	Target	0.041666667	1.4069856	0.4945
hsa-miR-604-001567	hsa-miR-604	hsa-miR-604	MIMAT0003272	Target	0	0.973224616	0.9725
hsa-miR-605-001568	hsa-miR-605	hsa-miR-605	MIMAT0003273	Target	0.166666667	643.8116827	0.232
hsa-miR-606-001569	hsa-miR-606	hsa-miR-606	MIMAT0003274	Target	0	2.404083395	0.0289
hsa-miR-607-001570	hsa-miR-607	hsa-miR-607	MIMAT0003275	Target	0.041666667	4.60358E-10	0.3434
hsa-miR-608-001571	hsa-miR-608	hsa-miR-608	MIMAT0003276	Target	0.041666667	40908788.52	0.3356
hsa-miR-609-001573	hsa-miR-609	hsa-miR-609	MIMAT0003277	Target	0.083333333	136.1533508	0.339
hsa-miR-613-001586	hsa-miR-613	hsa-miR-613	MIMAT0003281	Target	0.083333333	425518031.1	0.3074

hsa-miR-614-001587	hsa-miR-614	hsa-miR-614	MIMAT0003282	Target	0.041666667	3.29693E-07	0.3434
hsa-miR-615-5p-002353	hsa-miR-615-5p	hsa-miR-615-5p	MIMAT0004804	Target	0	2.404083395	0.0289
hsa-miR-616-001589	hsa-miR-616	hsa-miR-616-5p	MIMAT0003284	Target	0.083333333	1.442020478	0.6397
hsa-miR-616-002414	hsa-miR-616	hsa-miR-616-3p	MIMAT0004805	Target	0.083333333	39.34277079	0.271
hsa-miR-617-001591	hsa-miR-617	hsa-miR-617	MIMAT0003286	Target	0.041666667	2710056521	0.3356
hsa-miR-618-001593	hsa-miR-618	hsa-miR-618	MIMAT0003287	Target	0.166666667	6.327273919	0.2428
hsa-miR-620-002672	hsa-miR-620	hsa-miR-620	MIMAT0003289	Target	0	2.404083395	0.0289
hsa-miR-621-001598	hsa-miR-621	hsa-miR-621	MIMAT0003290	Target	0	2.404083395	0.0289
hsa-miR-622-001553	hsa-miR-622	hsa-miR-622	MIMAT0003291	Target	0	0.855408994	0.8326
hsa-miR-623-001555	hsa-miR-623	hsa-miR-623	MIMAT0003292	Target	0.166666667	0.441543742	0.4401
hsa-miR-624-001557	hsa-miR-624	hsa-miR-624-5p	MIMAT0003293	Target	0.083333333	2055811692	0.3356
hsa-miR-624-002430	hsa-miR-624	hsa-miR-624-3p	MIMAT0004807	Target	0.041666667	55.11719714	0.3228
hsa-miR-625#-002432	hsa-miR-625*	hsa-miR-625-3p	MIMAT0004808	Target	1	5.411715766	0.0021
hsa-miR-625-002431	hsa-miR-625	hsa-miR-625-5p	MIMAT0003294	Target	0.041666667	1.367197246	0.6863
hsa-miR-626-001559	hsa-miR-626	hsa-miR-626	MIMAT0003295	Target	0	2.404083395	0.0289
hsa-miR-627-001560	hsa-miR-627	hsa-miR-627	MIMAT0003296	Target	0	1.139193354	0.8467
hsa-miR-628-3p-002434	hsa-miR-628-3p	hsa-miR-628-3p	MIMAT0003297	Target	0.416666667	0.925466625	0.9092
hsa-miR-628-5p-002433	hsa-miR-628-5p	hsa-miR-628-5p	MIMAT0004809	Target	0.166666667	34537834.6	0.3356
hsa-miR-629-001562	hsa-miR-629	hsa-miR-629-3p	MIMAT0003298	Target	0.541666667	4.605537359	0.0772
hsa-miR-629-002436	hsa-miR-629	hsa-miR-629-5p	MIMAT0004810	Target	0.5	0.710798752	0.5507
hsa-miR-630-001563	hsa-miR-630	hsa-miR-630	MIMAT0003299	Target	0.041666667	0.689517912	0.6725
hsa-miR-631-001564	hsa-miR-631	hsa-miR-631	MIMAT0003300	Target	0	2.404083395	0.0289
hsa-miR-633-001574	hsa-miR-633	hsa-miR-633	MIMAT0003303	Target	0.041666667	3.32023E-10	0.3434
hsa-miR-634-001576	hsa-miR-634	hsa-miR-634	MIMAT0003304	Target	0	2.404083395	0.0289
hsa-miR-635-001578	hsa-miR-635	hsa-miR-635	MIMAT0003305	Target	0	2.404083395	0.0289
hsa-miR-636-002088	hsa-miR-636	hsa-miR-636	MIMAT0003306	Target	1	1.212160593	0.8938
hsa-miR-637-001581	hsa-miR-637	hsa-miR-637	MIMAT0003307	Target	0	2.404083395	0.0289
hsa-miR-638-001582	hsa-miR-638	hsa-miR-638	MIMAT0003308	Target	0.625	1.257105067	0.6996
hsa-miR-639-001583	hsa-miR-639	hsa-miR-639	MIMAT0003309	Target	0	2.404083395	0.0289
hsa-miR-640-001584	hsa-miR-640	hsa-miR-640	MIMAT0003310	Target	0	2.404083395	0.0289
hsa-miR-641-001585	hsa-miR-641	hsa-miR-641	MIMAT0003311	Target	0	2.404083395	0.0289
hsa-miR-642-001592	hsa-miR-642	hsa-miR-642a-5p	MIMAT0003312	Target	0.583333333	3.181036066	0.0136
hsa-miR-643-001594	hsa-miR-643	hsa-miR-643	MIMAT0003313	Target	0.041666667	4.332302392	0.2119
hsa-miR-644-001596	hsa-miR-644	hsa-miR-644a	MIMAT0003314	Target	0	2.404083395	0.0289
hsa-miR-645-001597	hsa-miR-645	hsa-miR-645	MIMAT0003315	Target	0.958333333	1.40960493	0.4267
hsa-miR-646-001599	hsa-miR-646	hsa-miR-646	MIMAT0003316	Target	0	2.404083395	0.0289
hsa-miR-647-001600	hsa-miR-647	hsa-miR-647	MIMAT0003317	Target	0	2.404083395	0.0289
hsa-miR-648-001601	hsa-miR-648	hsa-miR-648	MIMAT0003318	Target	0	2.404083395	0.0289
hsa-miR-649-001602	hsa-miR-649	hsa-miR-649	MIMAT0003319	Target	0	2.404083395	0.0289
hsa-miR-650-001603	hsa-miR-650	hsa-miR-650	MIMAT0003320	Target	0	2.404083395	0.0289
hsa-miR-651-001604	hsa-miR-651	hsa-miR-651	MIMAT0003321	Target	0	1.487936169	0.3355
hsa-miR-652-002352	hsa-miR-652	hsa-miR-652-3p	MIMAT0003322	Target	0.25	7.859683156	0.1602
hsa-miR-653-002292	hsa-miR-653	hsa-miR-653	MIMAT0003328	Target	0	2.835553911	0.0103
hsa-miR-654-001611	hsa-miR-654	hsa-miR-654-5p	MIMAT0003330	Target	0	2.404083395	0.0289
hsa-miR-654-3p-002239	hsa-miR-654-3p	hsa-miR-654-3p	MIMAT0004814	Target	0.083333333	1.178379033	0.8992
hsa-miR-655-001612	hsa-miR-655	hsa-miR-655	MIMAT0003331	Target	0.041666667	3.302274014	0.0932
hsa-miR-656-001510	hsa-miR-656	hsa-miR-656	MIMAT0003332	Target	0.041666667	17.24617567	0.2921

hsa-miR-657-001512	hsa-miR-657	hsa-miR-657	MIMAT0003335	Target	0.416666667	3.668736734	0.0973
hsa-miR-658-001513	hsa-miR-658	hsa-miR-658	MIMAT0003336	Target	0	2.404083395	0.0289
hsa-miR-659-001514	hsa-miR-659	hsa-miR-659-3p	MIMAT0003337	Target	0	2.404083395	0.0289
hsa-miR-660-001515	hsa-miR-660	hsa-miR-660-5p	MIMAT0003338	Target	1	1.034911018	0.8996
hsa-miR-661-001606	hsa-miR-661	hsa-miR-661	MIMAT0003324	Target	0.125	2130.010486	0.3172
hsa-miR-662-001607	hsa-miR-662	hsa-miR-662	MIMAT0003325	Target	0	2.404083395	0.0289
hsa-miR-663B-002857	hsa-miR-663B	NA	NA	Target	0	2.404083395	0.0289
hsa-miR-664-002897	hsa-miR-664	hsa-miR-664-3p	MIMAT0005949	Target	0	2.404083395	0.0289
hsa-miR-665-002681	hsa-miR-665	hsa-miR-665	MIMAT0004952	Target	0	2.404083395	0.0289
hsa-miR-668-001992	hsa-miR-668	hsa-miR-668	MIMAT0003881	Target	0	2.404083395	0.0289
hsa-miR-671-3p-002322	hsa-miR-671-3p	hsa-miR-671-3p	MIMAT0004819	Target	0.125	4.815596064	0.01
hsa-miR-672-002327	hsa-miR-672	NA	NA	Target	0	2.404083395	0.0289
hsa-miR-674-002021	hsa-miR-674	NA	NA	Target	0	2.404083395	0.0289
hsa-miR-675-002005	hsa-miR-675	hsa-miR-675-5p	MIMAT0004284	Target	0	2.404083395	0.0289
hsa-miR-7-2#-002314	hsa-miR-7-2*	hsa-miR-7-2-3p	MIMAT0004554	Target	0	2.404083395	0.0289
hsa-miR-708#-002342	hsa-miR-708*	hsa-miR-708-3p	MIMAT0004927	Target	0	2.404083395	0.0289
hsa-miR-708-002341	hsa-miR-708	hsa-miR-708-5p	MIMAT0004926	Target	0.083333333	0.627357398	0.6638
hsa-miR-720-002895	hsa-miR-720	hsa-miR-720	MIMAT0005954	Target	1	4.84552035	0.1447
hsa-miR-744#-002325	hsa-miR-744*	hsa-miR-744-3p	MIMAT0004946	Target	0	5.956698742	0.1998
hsa-miR-744-002324	hsa-miR-744	hsa-miR-744-5p	MIMAT0004945	Target	0.75	1.373267922	0.4763
hsa-miR-758-001990	hsa-miR-758	hsa-miR-758	MIMAT0003879	Target	0.083333333	0.449745385	0.5007
hsa-miR-765-002643	hsa-miR-765	hsa-miR-765	MIMAT0003945	Target	0	0.969296321	0.9688
hsa-miR-766-001986	hsa-miR-766	hsa-miR-766-3p	MIMAT0003888	Target	0.458333333	3074548.664	0.3356
hsa-miR-767-3p-001995	hsa-miR-767-3p	hsa-miR-767-3p	MIMAT0003883	Target	0	2.404083395	0.0289
hsa-miR-767-5p-001993	hsa-miR-767-5p	hsa-miR-767-5p	MIMAT0003882	Target	0	2.404083395	0.0289
hsa-miR-769-3p-002003	hsa-miR-769-3p	hsa-miR-769-3p	MIMAT0003887	Target	0	5.026831976	0.1946
hsa-miR-769-5p-001998	hsa-miR-769-5p	hsa-miR-769-5p	MIMAT0003886	Target	0.041666667	10.59492968	0.2117
hsa-miR-770-5p-002002	hsa-miR-770-5p	hsa-miR-770-5p	MIMAT0003948	Target	0	2.404083395	0.0289
hsa-miR-802-002004	hsa-miR-802	hsa-miR-802	MIMAT0004185	Target	0	2.844729883	0.3555
hsa-miR-871-002354	hsa-miR-871	NA	NA	Target	0	2.404083395	0.0289
hsa-miR-872-002264	hsa-miR-872	NA	NA	Target	0	2.404083395	0.0289
hsa-miR-873-002356	hsa-miR-873	hsa-miR-873-5p	MIMAT0004953	Target	0	0.518615606	0.4288
hsa-miR-874-002268	hsa-miR-874	hsa-miR-874	MIMAT0004911	Target	0	2.404083395	0.0289
hsa-miR-875-3p-002204	hsa-miR-875-3p	hsa-miR-875-3p	MIMAT0004923	Target	0	2.404083395	0.0289
hsa-miR-875-5p-002203	hsa-miR-875-5p	hsa-miR-875-5p	MIMAT0004922	Target	0.416666667	5290099.295	0.1548
hsa-miR-876-3p-002225	hsa-miR-876-3p	hsa-miR-876-3p	MIMAT0004925	Target	0	2.404083395	0.0289
hsa-miR-876-5p-002205	hsa-miR-876-5p	hsa-miR-876-5p	MIMAT0004924	Target	0	2.404083395	0.0289
hsa-miR-885-3p-002372	hsa-miR-885-3p	hsa-miR-885-3p	MIMAT0004948	Target	0	2.404083395	0.0289
hsa-miR-885-5p-002296	hsa-miR-885-5p	hsa-miR-885-5p	MIMAT0004947	Target	1	3.250037561	0.0677
hsa-miR-886-3p-002194	hsa-miR-886-3p	NA	NA	Target	0.75	2.07612785	0.1087
hsa-miR-886-5p-002193	hsa-miR-886-5p	NA	NA	Target	0.291666667	0.828852244	0.7229
hsa-miR-887-002374	hsa-miR-887	hsa-miR-887	MIMAT0004951	Target	0	2.404083395	0.0289
hsa-miR-888#-002213	hsa-miR-888*	hsa-miR-888-3p	MIMAT0004917	Target	0	2.404083395	0.0289
hsa-miR-888-002212	hsa-miR-888	hsa-miR-888-5p	MIMAT0004916	Target	0.041666667	3.806E-06	0.3434
hsa-miR-889-002202	hsa-miR-889	hsa-miR-889	MIMAT0004921	Target	0	2.404083395	0.0289
hsa-miR-890-002209	hsa-miR-890	hsa-miR-890	MIMAT0004912	Target	0.125	1260.264619	0.0958
hsa-miR-891a-002191	hsa-miR-891a	hsa-miR-891a	MIMAT0004902	Target	0	10.28250679	0.2668

hsa-miR-891b-002210	hsa-miR-891b	hsa-miR-891b	MIMAT0004913	Target	0	2.404083395	0.0289
hsa-miR-892a-002195	hsa-miR-892a	hsa-miR-892a	MIMAT0004907	Target	0	2.404083395	0.0289
hsa-miR-892b-002214	hsa-miR-892b	hsa-miR-892b	MIMAT0004918	Target	0.041666667	4.309462127	0.095
hsa-miR-9#-002231	hsa-miR-9*	hsa-miR-9-3p	MIMAT0000442	Target	0.583333333	1.102695829	0.8342
hsa-miR-9-000583	hsa-miR-9	hsa-miR-9-5p	MIMAT0000441	Target	0.458333333	11.05856029	0.1756
hsa-miR-920-002150	hsa-miR-920	hsa-miR-920	MIMAT0004970	Target	0	2.404083395	0.0289
hsa-miR-921-002151	hsa-miR-921	hsa-miR-921	MIMAT0004971	Target	0	2.404083395	0.0289
hsa-miR-922-002152	hsa-miR-922	hsa-miR-922	MIMAT0004972	Target	0	2.404083395	0.0289
hsa-miR-924-002154	hsa-miR-924	hsa-miR-924	MIMAT0004974	Target	0	4.003515914	0.0896
hsa-miR-92a-000431	hsa-miR-92a	hsa-miR-92a-3p	MIMAT0000092	Target	0.958333333	0.96417926	0.8842
hsa-miR-92a-1#-002137	hsa-miR-92a-1*	hsa-miR-92a-1-5p	MIMAT0004507	Target	0	0.99926372	0.9989
hsa-miR-92a-2#-002138	hsa-miR-92a-2*	hsa-miR-92a-2-5p	MIMAT0004508	Target	0	2.404083395	0.0289
hsa-miR-92b#-002343	hsa-miR-92b*	hsa-miR-92b-5p	MIMAT0004792	Target	0.041666667	3.50869E-07	0.3434
hsa-miR-93#-002139	hsa-miR-93*	hsa-miR-93-3p	MIMAT0004509	Target	0.791666667	2.35203679	0.0958
hsa-miR-933-002176	hsa-miR-933	hsa-miR-933	MIMAT0004976	Target	0	2.404083395	0.0289
hsa-miR-934-002177	hsa-miR-934	hsa-miR-934	MIMAT0004977	Target	0	2.404083395	0.0289
hsa-miR-935-002178	hsa-miR-935	hsa-miR-935	MIMAT0004978	Target	0	2.404083395	0.0289
hsa-miR-936-002179	hsa-miR-936	hsa-miR-936	MIMAT0004979	Target	0	2.404083395	0.0289
hsa-miR-937-002180	hsa-miR-937	hsa-miR-937	MIMAT0004980	Target	0.041666667	0.23724305	0.4168
hsa-miR-938-002181	hsa-miR-938	hsa-miR-938	MIMAT0004981	Target	0	1.517127287	0.324
hsa-miR-939-002182	hsa-miR-939	hsa-miR-939	MIMAT0004982	Target	0	9.61109055	0.2594
hsa-miR-941-002183	hsa-miR-941	hsa-miR-941	MIMAT0004984	Target	0	2.404083395	0.0289
hsa-miR-942-002187	hsa-miR-942	hsa-miR-942	MIMAT0004985	Target	0.416666667	0.794226516	0.6233
hsa-miR-943-002188	hsa-miR-943	hsa-miR-943	MIMAT0004986	Target	0	2.404083395	0.0289
hsa-miR-944-002189	hsa-miR-944	hsa-miR-944	MIMAT0004987	Target	0	1.251485085	0.5505
hsa-miR-95-000433	hsa-miR-95	hsa-miR-95	MIMAT0000094	Target	0.708333333	2.355061013	0.0448
hsa-miR-96#-002140	hsa-miR-96*	hsa-miR-96-3p	MIMAT0004510	Target	0	2.404083395	0.0289
hsa-miR-98-000577	hsa-miR-98	hsa-miR-98	MIMAT0000096	Target	0.125	0.000180809	0.3411
hsa-miR-99a#-002141	hsa-miR-99a*	hsa-miR-99a-3p	MIMAT0004511	Target	0.041666667	4.834223409	0.0862
hsa-miR-99a-000435	hsa-miR-99a	hsa-miR-99a-5p	MIMAT0000097	Target	0.666666667	1.770044454	0.6088
hsa-miR-99b#-002196	hsa-miR-99b*	hsa-miR-99b-3p	MIMAT0004678	Target	0.083333333	0.167390391	0.4217
hsa-miR-99b-000436	hsa-miR-99b	hsa-miR-99b-5p	MIMAT0000689	Target	0.958333333	2.255351627	0.1112
mmu-let-7d#-001178	mmu-let-7d*	mmu-let-7d-3p	MIMAT0000384	Target	0	2.404083395	0.0289
mmu-miR-124a-001182	mmu-miR-124a	mmu-miR-124-3p	MIMAT0000134	Target	0.041666667	0.008488519	0.3427
mmu-miR-129-3p-001184	mmu-miR-129-3p	mmu-miR-129-2-3p	MIMAT0000544	Target	0.166666667	1.06326E-08	0.3427
mmu-miR-134-001186	mmu-miR-134	mmu-miR-134-5p	MIMAT0000146	Target	0.166666667	0.685503283	0.6734
mmu-miR-137-001129	mmu-miR-137	mmu-miR-137-3p	MIMAT0000149	Target	0.041666667	0.58912889	0.6025
mmu-miR-140-001187	mmu-miR-140	mmu-miR-140-5p	MIMAT0000151	Target	0.958333333	7.63875E-07	0.3434
mmu-miR-153-001191	mmu-miR-153	mmu-miR-153-3p	MIMAT0000163	Target	0.125	676053423.1	0.1716
mmu-miR-187-001193	mmu-miR-187	mmu-miR-187-3p	MIMAT0000216	Target	0.083333333	9.7799E-06	0.3434
mmu-miR-374-5p-001319	mmu-miR-374-5p	mmu-miR-374-5p	MIMAT0003727	Target	0.875	1.348987439	0.622
mmu-miR-379-001138	mmu-miR-379	mmu-miR-379-5p	MIMAT0000743	Target	0.041666667	17.15673575	0.2919
mmu-miR-451-001141	mmu-miR-451	mmu-miR-451	MIMAT0001632	Target	1	0.979293988	0.9412
mmu-miR-491-001630	mmu-miR-491	mmu-miR-491-5p	MIMAT0003486	Target	0.291666667	9.460683997	0.1013
mmu-miR-495-001663	mmu-miR-495	mmu-miR-495-3p	MIMAT0003456	Target	0.083333333	271.688564	0.2649
mmu-miR-496-001953	mmu-miR-496	mmu-miR-496-3p	MIMAT0003738	Target	0.041666667	143.4085862	0.3305
mmu-miR-499-001352	mmu-miR-499	mmu-miR-499-5p	MIMAT0003482	Target	0	2.404083395	0.0289

mmu-miR-615-001960	mmu-miR-615	mmu-miR-615-3p	MIMAT0003783	Target	0	2.404083395	0.0289
mmu-miR-93-001090	mmu-miR-93	mmu-miR-93-5p	MIMAT0000540	Target	1	0.98457004	0.9503
mmu-miR-96-000186	mmu-miR-96	mmu-miR-96-5p	MIMAT0000541	Target	0.083333333	0.346453683	0.4098
rno-miR-29c#-001818	rno-miR-29c*	rno-miR-29c*	MIMAT0003154	Target	0.041666667	0.925011806	0.9208
rno-miR-7#-001338	rno-miR-7*	rno-miR-7a-1*	MIMAT0000607	Target	0.458333333	0.753125178	0.7183
RNU44-001094	RNU44	NA	NA	Target	0.083333333	0.011698994	0.3265
RNU48-001006	RNU48	NA	NA	Target	0.333333333	2.491113155	0.1742
U6 snRNA-001973	U6 snRNA	NA	NA	Target	1	6.820337956	0.1295

Table S2. miRNA expression profile in validation stage.

Data	miRNA	Total			Control			Case			P-value
		Median	25% Percentile	75% Percentile	Median	25% Percentile	75% Percentile	Median	25% Percentile	75% Percentile	
$2^{-\Delta Ct}$	hsa-miR-26b-5p	0.0462	0.0310	0.0708	0.0425	0.0293	0.0567	0.0544	0.0320	0.0922	7.646E-05
	hsa-miR-146b-5p	0.0177	0.0109	0.0299	0.0167	0.0106	0.0228	0.0209	0.0114	0.0531	4.118E-04
	hsa-miR-191-5p	0.1237	0.0833	0.2073	0.1062	0.0797	0.1550	0.1421	0.0893	0.3523	1.944E-06
	hsa-miR-484	1.5052	0.9202	2.6947	1.0943	0.7658	1.5640	2.1435	1.3241	3.9313	0.000E+00
	hsa-miR-574-3p	0.0288	0.0166	0.0533	0.0243	0.0161	0.0349	0.0369	0.0186	0.0843	7.939E-07
	hsa-miR-625-3p	0.0103	0.0053	0.0232	0.0066	0.0032	0.0115	0.0169	0.0087	0.0414	0.000E+00
ΔCt	hsa-miR-26b-5p	4.4350	3.8200	5.0100	4.5550	4.1400	5.0950	4.2000	3.4400	4.9650	7.646E-05
	hsa-miR-146b-5p	5.8200	5.0650	6.5150	5.9050	5.4550	6.5650	5.5800	4.2350	6.4500	4.118E-04
	hsa-miR-191-5p	3.0150	2.2700	3.5850	3.2350	2.6900	3.6500	2.8150	1.5050	3.4850	1.944E-06
	hsa-miR-484	-0.5900	-1.4300	0.1200	-0.1300	-0.6450	0.3850	-1.1000	-1.9750	-0.4050	0.000E+00
	hsa-miR-574-3p	5.1200	4.2300	5.9100	5.3650	4.8400	5.9600	4.7600	3.5700	5.7500	7.939E-07
	hsa-miR-625-3p	6.5950	5.4300	7.5500	7.2400	6.4400	8.2900	5.8900	4.5950	6.8450	0.000E+00

Table S3. Efficacy of the classifier.

	Accuracy	Sensitivity	Specificity	AUC
**Validation***	0.7218（0.7117，0.7319）	0.7343（0.7172，0.7515）	0.7078（0.6862，0.7293）	0.7211（0.7108，0.7313）
**Independent Validation**	0.7021	0.8077	0.5714	0.6896

* Data are means and 95% confidence intervals.

Table S4. miRNA expression profile in independent validation stage.

Data	miRNA	Total			Control			Case			P-value
		Median	25% Percentile	75% Percentile	Median	25% Percentile	75% Percentile	Median	25% Percentile	75% Percentile	
$2^{-\Delta Ct}$	hsa-miR-26b-5p	0.0352	0.0266	0.0583	0.0274	0.0156	0.0337	0.0517	0.0352	0.0884	3.793E-05
	hsa-miR-146b-5p	0.0089	0.0061	0.0199	0.0066	0.0046	0.0083	0.0167	0.0092	0.0430	2.988E-04
	hsa-miR-191-5p	0.1340	0.0775	0.2365	0.0780	0.0684	0.1127	0.1933	0.1387	0.4175	3.008E-05
	hsa-miR-484	1.9185	1.0644	3.8637	1.1567	0.8293	1.5476	2.4797	1.8532	4.3772	3.040E-03
	hsa-miR-574-3p	0.0324	0.0215	0.0552	0.0294	0.0142	0.0415	0.0341	0.0276	0.0890	2.331E-02
	hsa-miR-625-3p	0.0215	0.0130	0.0328	0.0167	0.0083	0.0245	0.0270	0.0165	0.0490	4.893E-03
ΔCt	hsa-miR-26b-5p	4.8300	4.1000	5.2300	5.1900	4.8900	6.0000	4.2750	3.5000	4.8300	3.793E-05
	hsa-miR-146b-5p	6.8100	5.6500	7.3500	7.2500	6.9100	7.7700	5.9050	4.5400	6.7700	2.988E-04
	hsa-miR-191-5p	2.9000	2.0800	3.6900	3.6800	3.1500	3.8700	2.3750	1.2600	2.8500	3.008E-05
	hsa-miR-484	-0.9400	-1.9500	-0.0900	-0.2100	-0.6300	0.2700	-1.3100	-2.1300	-0.8900	3.040E-03
	hsa-miR-574-3p	4.9500	4.1800	5.5400	5.0900	4.5900	6.1400	4.8750	3.4900	5.1800	2.331E-02
	hsa-miR-625-3p	5.5400	4.9300	6.2700	5.9000	5.3500	6.9100	5.2100	4.3500	5.9200	4.893E-03

Table S5. Predicted target genes.

Total predicted target genes	hsa-miR-26b-5p predicted target genes	hsa-miR-146b-5p predicted target genes	hsa-miR-191-5p predicted target genes	hsa-miR-484 predicted target genes	hsa-miR-574-3p predicted target genes	hsa-miR-625-3p predicted target genes
HMGA1	*HMGA1*	*TRAF6*	*MAPRE2*	*PTGER4*	*BACE1*	*MAP2K6*
TET2	*TET2*	*IRAK1*	*PLCD1*	*CREB3L3*	*STRN3*	*EGFLAM*
FAM98A	*FAM98A*	*DCAF12*	*TMOD2*	*MYCBP2*	*SNCB*	*IRAK2*
ZDHHC6	*ZDHHC6*	*IGSF1*	*NRCAM*	*AVL9*		*ZFPM2*
TNRC6B	*TNRC6B*	*HIPK3*	*AMMECR1*	*SORBS2*		*CAMK4*
NABP1	*NABP1*	*LCOR*	*TJP1*	*SLC20A2*		*KIF13A*
KLHL42	*KLHL42*	*KLF7*	*ZCCHC14*	*PCDH19*		*ALAS1*
CASZ1	*CASZ1*	*ZBTB2*	*ZBTB34*	*HIVEP2*		*ZSCAN29*
STK39	*STK39*	*ZNF367*	*SATB1*	*HK2*		*EXOC8*
ZNF462	*ZNF462*	*MMP16*	*NEURL4*	*DACH1*		*DNAJA1*
SLC7A11	*SLC7A11*	*SIAH2*	*CDK6*	*LAT*		*PAWR*
KIAA2013	*KIAA2013*	*CD80*	*MAPRE3*	*MEN1*		*GALNT1*
MFHAS1	*MFHAS1*	*SLC10A3*	*WIZ*	*SNRNP200*		*PYGO1*
ATAD2B	*ATAD2B*	*HNRNPD*	*BDNF*	*DCBLD2*		*RBM12B*
CEP350	*CEP350*	*ZDHHC13*	*TAF5*	*C17orf112*		*USP9X*
SENP5	*SENP5*	*RFX7*		*CYB561D1*		*CPEB4*
E2F7	*E2F7*	*FBXW2*		*TRAT1*		*RBM27*
OTUD4	*OTUD4*	*ERBB4*		*MGAT5*		*GSE1*
PRKCD	*PRKCD*	*ABL2*		*SNN*		*ZC3H6*
FRMD4B	*FRMD4B*	*BCORL1*		*FGF1*		*PRPF4*
ACBD5	*ACBD5*	*MYBL1*		*TSGA10*		*HMGB3*
RBM24	*RBM24*	*EIF4G2*		*ANAPC7*		*ABHD17B*
STRADB	*STRADB*	*SEC23IP*		*KIAA1549*		*CYP3A7*
HGF	*HGF*	*WWC2*		*IL21R*		*THRAP3*
TET3	*TET3*	*RARB*		*RIMS2*		*ZBTB44*
LARP1	*LARP1*	*APPL1*		*KCNJ2*		*DNAJA2*
GSK3B	*GSK3B*	*NUMB*		*APLN*		*RUNDC3B*
EPC1	*EPC1*	*ZNRF3*		*XPO4*		*ZC3H12B*
CDK8	*CDK8*	*CDKN2AIP*		*ADAM33*		*WNK3*
TRIB2	*TRIB2*	*ZNF652*		*TOPBP1*		*PPP6C*
SMAD1	*SMAD1*	*LRP2*		*S100PBP*		*TGFBR1*
PALM3	*PALM3*	*ROBO1*		*ACVR1B*		*LHX8*
ST6GAL2	*ST6GAL2*	*DDHD1*		*IFNAR1*		*CNTN4*
THRAP3	*THRAP3*	*C16orf72*		*MTF2*		*LEPROTL1*
CHFR	*CHFR*	*SYT1*		*SERTAD1*		*FLG2*
STYX	*STYX*	*NOVA1*		*ZNF37A*		*OSBPL8*
SNN	*SNN*	*ZNF512B*		*FOSL2*		*TWISTNB*
SRP19	*SRP19*	*TMEM120B*		*KMT2A*		*RBFOX2*
FGD1	*FGD1*	*PIP5K1B*		*PLEKHG4*		*ATF7IP*
KCNJ2	*KCNJ2*	*LFNG*		*NOL9*		*PIP5K1B*
SULF1	*SULF1*	*PPP1R11*		*XKR9*		*FAM151B*
ULK2	*ULK2*	*ZNF532*		*LRPAP1*		*LGR5*
PTEN	*PTEN*	*STRBP*		*STRN*		*GABRA4*

EZH2	*EZH2*	*AAK1*		*HIPK1*		*NEUROD1*
NAP1L5	*NAP1L5*	*ACKR2*		*HOXA5*		*FAM19A1*
FBXO11	*FBXO11*	*RIMS2*		*NRG2*		*MAD2L1*
CHAC1	*CHAC1*	*LRRC15*		*LAMB3*		*STRN*
DCDC2	*DCDC2*	*XKR4*		*PTPRE*		*COL4A3BP*
SLC25A16	*SLC25A16*	*CDS1*		*CACTIN*		*ECT2L*
ATP11C	*ATP11C*	*GALNT10*		*GRAMD1C*		*TJP1*
MAB21L1	*MAB21L1*	*VASN*		*WFS1*		*SLC39A10*
NAB1	*NAB1*	*RABGAP1*		*ORAI2*		*NDUFAF5*
TOB1	*TOB1*	*TBC1D20*		*CSRNP2*		*ZBTB2*
ZIC5	*ZIC5*	*PTGFRN*		*KCNJ14*		*WDR76*
ZBTB18	*ZBTB18*	*SORT1*		*CRTC3*		*HOOK3*
RNF6	*RNF6*	*GDNF*		*IL20RB*		*ZSWIM6*
UBR3	*UBR3*	*SCN3B*		*EMX1*		*ASIC5*
MTDH	*MTDH*	*ARMC8*		*GRM1*		*GGPS1*
BAZ2B	*BAZ2B*	*ESYT2*		*RNF24*		*SMC6*
REEP3	*REEP3*	*SAMD8*		*PRTG*		*ATRNL1*
SRCAP	*SRCAP*	*CARD10*		*TPRX1*		*B3GNT5*
CLASP2	*CLASP2*	*CCDC6*		*MMP14*		*FOXN2*
THAP2	*THAP2*	*FBXO28*		*CRTC2*		*HOXA7*
UBE4B	*UBE4B*	*RPA3*		*RHOBTB1*		*HIF1A*
ADAM23	*ADAM23*	*ZFYVE1*		*APBA1*		*IAPP*
ACSL3	*ACSL3*	*BMPR1A*		*PIKFYVE*		*SRGAP1*
ADAM19	*ADAM19*	*PRKAA2*		*PCDH7*		*STARD4*
PLOD2	*PLOD2*	*SRSF12*		*CDK9*		*SOX11*
CCDC6	*CCDC6*	*MYT1*		*MBD1*		*TNFSF11*
FA2H	*FA2H*	*USP3*		*BSDC1*		*ANKRD20A3*
NHS	*NHS*	*PRX*		*ABR*		*ATG3*
PLCB1	*PLCB1*	*ZNRF2*		*TCHP*		*ESCO1*
MTM1	*MTM1*	*SEMA3G*		*DBNDD2*		*TMEFF2*
ATP1A2	*ATP1A2*	*PTPRA*		*POT1*		*DESI2*
CPSF2	*CPSF2*	*GRID1*		*PIK3CD*		*ATRN*
ACVR1C	*ACVR1C*			*GPR63*		*ELL2*
MRAS	*MRAS*			*CPLX3*		*DGKH*
CHORDC1	*CHORDC1*			*FBXO11*		*KLHL4*
RSPRY1	*RSPRY1*			*RBM24*		*FMNL2*
CILP	*CILP*			*SORBS1*		*ANKRD20A4*
ANKS1A	*ANKS1A*			*ATP6V0A2*		*ETV1*
POLR3G	*POLR3G*			*MED8*		*RBM17*
NUDT11	*NUDT11*			*PNRC1*		*DPY19L2*
EPHA2	*EPHA2*			*NCR3*		*FREM2*
COL19A1	*COL19A1*			*UAP1L1*		*SMIM15*
PITPNC1	*PITPNC1*			*AMPD2*		*MXI1*
USP15	*USP15*			*CYB5RL*		
ZSWIM6	*ZSWIM6*			*ZSWIM6*		
ITGA5	*ITGA5*			*RNF8*		
PDCD10	*PDCD10*			*DAG1*		

LOXL2	*LOXL2*			*LAIR1*		
ARPP19	*ARPP19*			*PRM1*		
RPGR	*RPGR*			*WWC1*		
SRGAP1	*SRGAP1*			*SPSB4*		
FRAT2	*FRAT2*			*TPT1*		
FLVCR1	*FLVCR1*			*DISC1*		
CCNJL	*CCNJL*			*PYGO2*		
FBXL19	*FBXL19*			*CTAGE1*		
ATF2	*ATF2*			*LPL*		
PTPRD	*PTPRD*			*ZNF667*		
UBN2	*UBN2*			*C22orf46*		
VANGL2	*VANGL2*			*THBD*		
ZNF410	*ZNF410*			*GCSAM*		
G3BP2	*G3BP2*			*EIF4G2*		
ZNF598	*ZNF598*			*FAM71E2*		
MSMO1	*MSMO1*			*SAMD4B*		
RHOQ	*RHOQ*			*FRMD5*		
OSBPL11	*OSBPL11*			*TNFSF9*		
ADAM17	*ADAM17*			*TSTD2*		
CCDC28A	*CCDC28A*			*FLOT1*		
LIN28B	*LIN28B*			*CRTAP*		
HOXA5	*HOXA5*			*DDX6*		
ADM	*ADM*			*STARD3NL*		
MIER3	*MIER3*			*UBE2R2*		
SSX2IP	*SSX2IP*			*CSF1*		
ERC2	*ERC2*			*CACHD1*		
LRRC2	*LRRC2*			*MBNL3*		
FAM136A	*FAM136A*			*ENY2*		
PRKCQ	*PRKCQ*			*IGBP1*		
CTTNBP2NL	*CTTNBP2NL*			*HLA-DOB*		
RPS6KA6	*RPS6KA6*			*PKNOX1*		
CD200	*CD200*			*KSR2*		
LSM12	*LSM12*			*RABGAP1L*		
CAMSAP1	*CAMSAP1*			*GJC1*		
TTC13	*TTC13*			*SMARCA2*		
GPR52	*GPR52*			*TK2*		
TNRC6C	*TNRC6C*			*PDGFA*		
RBM46	*RBM46*			*LMAN2L*		
ANKS1B	*ANKS1B*			*SOX5*		
KBTBD8	*KBTBD8*			*KCNC1*		
GPALPP1	*GPALPP1*			*ABLIM2*		
DNAJC21	*DNAJC21*			*ZMIZ1*		
SYT10	*SYT10*			*HHIPL2*		
TBC1D15	*TBC1D15*			*PITPNA*		
SAMD8	*SAMD8*			*SLC43A2*		
ART3	*ART3*			*TEX261*		
BOD1	*BOD1*			*THPO*		

POM121C	*POM121C*			*PRR14L*		
TNRC6A	*TNRC6A*			*SCP2*		
ALDH5A1	*ALDH5A1*			*CCNL1*		
BFAR	*BFAR*			*RFX3*		
CTH	*CTH*			*ST6GAL1*		
PWWP2A	*PWWP2A*			*HNF1A*		
HPGD	*HPGD*			*SLC17A9*		
RCBTB1	*RCBTB1*			*BCL7B*		
TTPAL	*TTPAL*			*CLOCK*		
PAN3	*PAN3*			*NCAN*		
DCUN1D3	*DCUN1D3*			*PPP6R3*		
ULK1	*ULK1*			*TSPAN17*		
NLK	*NLK*			*SLC6A1*		
MAPK6	*MAPK6*			*ALPK3*		
TMCC1	*TMCC1*			*CALM1*		
PTBP3	*PTBP3*			*FMNL3*		
NUS1	*NUS1*			*CCR9*		
PTPN13	*PTPN13*			*MPRIP*		
NAMPT	*NAMPT*			*LRTM2*		
MEX3B	*MEX3B*			*MCTP1*		
NAA15	*NAA15*			*ZFYVE1*		
DDX3X	*DDX3X*			*GLDN*		
UBE2H	*UBE2H*			*PAK3*		
CDK6	*CDK6*			*FNDC5*		
GMDS	*GMDS*			*MAP4K5*		
RCOR1	*RCOR1*			*ATP1B2*		
CDH20	*CDH20*			*TNRC6C*		
KPNA6	*KPNA6*			*ANO2*		
GMFB	*GMFB*			*WNT2B*		
MTX2	*MTX2*			*NFAM1*		
PHF6	*PHF6*			*MAPKAPK2*		
MAP7	*MAP7*			*DLG2*		
PAWR	*PAWR*			*SMIM7*		
SLC2A14	*SLC2A14*			*YTHDF3*		
TBC1D4	*TBC1D4*			*ADAM10*		
MARK1	*MARK1*			*SAMD8*		
HOXA9	*HOXA9*			*DCHS1*		
DOCK4	*DOCK4*			*TNR*		
ASPN	*ASPN*			*FAM178B*		
PTGS2	*PTGS2*			*PDE1C*		
ZNF148	*ZNF148*			*STC1*		
STAC2	*STAC2*			*TRPS1*		
DEPDC1	*DEPDC1*			*PEX16*		
UGT8	*UGT8*			*ZNF25*		
SAMD12	*SAMD12*			*PEA15*		
REST	*REST*			*TAF11*		
ABL2	*ABL2*			*CYB5B*		

GPSM1	*GPSM1*			*LTBP2*		
WNK3	*WNK3*			*LYSMD1*		
MAT2A	*MAT2A*			*VKORC1*		
MPP6	*MPP6*			*ZNF24*		
NTN4	*NTN4*			*ARF4*		
HAS3	*HAS3*			*ELMO2*		
ZFHX4	*ZFHX4*			*TBC1D24*		
MDN1	*MDN1*			*ZNF581*		
MXI1	*MXI1*			*MLXIP*		
UBE2G1	*UBE2G1*			*GPR155*		
EIF2S1	*EIF2S1*			*ABCB10*		
NUP50	*NUP50*			*PRRT2*		
MAP2	*MAP2*			*DPYSL2*		
PHF21A	*PHF21A*			*ABHD2*		
INHBB	*INHBB*			*TMEM184A*		
USP9X	*USP9X*			*LMX1B*		
AGPAT5	*AGPAT5*			*RAP2A*		
MAP3K9	*MAP3K9*			*TOX2*		
LNX2	*LNX2*			*TXNRD3*		
CREBZF	*CREBZF*			*PEX6*		
FAM8A1	*FAM8A1*			*UBR2*		
ETNK1	*ETNK1*			*WDR3*		
ZFC3H1	*ZFC3H1*			*GRPEL2*		
TNPO1	*TNPO1*			*MAP2*		
PHF20L1	*PHF20L1*			*DAGLA*		
RHOU	*RHOU*			*ALS2CL*		
COL10A1	*COL10A1*			*IPO11*		
RGS4	*RGS4*			*VIPR1*		
TFAP2A	*TFAP2A*			*KLHDC3*		
TBC1D30	*TBC1D30*			*RIMS3*		
BAK1	*BAK1*			*IL6R*		
TANC2	*TANC2*			*DNAJC5G*		
CXADR	*CXADR*			*EMC6*		
PEX13	*PEX13*			*B3GNT9*		
BID	*BID*			*EFR3B*		
BOD1L2	*BOD1L2*			*ETS1*		
PFKFB3	*PFKFB3*			*NUP153*		
RLF	*RLF*			*HOXA11*		
JAG1	*JAG1*			*SPOCK2*		
PATZ1	*PATZ1*			*KHK*		
PIM1	*PIM1*			*HYDIN*		
RBM20	*RBM20*			*SAMD5*		
TET1	*TET1*			*DENND5A*		
SH3D19	*SH3D19*			*STX5*		
RAP2C	*RAP2C*			*C9orf62*		
SERBP1	*SERBP1*			*PTPN14*		
TMEM106B	*TMEM106B*			*C1QTNF7*		

BCR	*BCR*			*NR6A1*		
RFX3	*RFX3*			*SNX17*		
PLEKHH1	*PLEKHH1*			*C3orf62*		
BBX	*BBX*			*OGDH*		
SLC38A2	*SLC38A2*			*EPHA5*		
DDX52	*DDX52*			*CNNM1*		
DMRT3	*DMRT3*			*CDYL2*		
FBXO28	*FBXO28*			*TGM2*		
G2E3	*G2E3*			*COLQ*		
HEPHL1	*HEPHL1*			*MINK1*		
CDK2AP1	*CDK2AP1*			*NFATC4*		
SFXN1	*SFXN1*			*SP6*		
DUSP5	*DUSP5*			*PROM2*		
GABRA4	*GABRA4*			*GPX1*		
DLG4	*DLG4*			*CEP170B*		
ANKRD52	*ANKRD52*			*LRRC32*		
BLOC1S2	*BLOC1S2*			*TRIM66*		
RNF141	*RNF141*			*TINF2*		
ITGB8	*ITGB8*			*NAT8L*		
PAK2	*PAK2*			*EIF4A2*		
SLC30A7	*SLC30A7*			*CARHSP1*		
HSPA8	*HSPA8*			*STEAP3*		
EP400	*EP400*			*NEO1*		
SLC9A2	*SLC9A2*			*TRIM74*		
ADAMTS19	*ADAMTS19*			*HS3ST3B1*		
SLC24A4	*SLC24A4*			*C7orf43*		
STRBP	*STRBP*			*TRIM67*		
RCN2	*RCN2*			*IRGQ*		
ZNF710	*ZNF710*			*SUSD2*		
DGKH	*DGKH*			*DCLK3*		
FAM98B	*FAM98B*			*CCNG1*		
RAB3IP	*RAB3IP*			*CLEC6A*		
GRAMD1C	*GRAMD1C*			*PPME1*		
EPS15	*EPS15*			*SH3PXD2A*		
NIPA1	*NIPA1*			*GATAD2B*		
ELAVL2	*ELAVL2*			*ATP7B*		
NIPAL2	*NIPAL2*			*EME1*		
AKAP7	*AKAP7*			*KIAA0513*		
HAO1	*HAO1*					
SLC33A1	*SLC33A1*					
BTBD7	*BTBD7*					
HELZ	*HELZ*					
HSPA4L	*HSPA4L*					
TP53INP1	*TP53INP1*					
PDE4B	*PDE4B*					
SACS	*SACS*					
PON1	*PON1*					

CPED1	*CPED1*					
PHF3	*PHF3*					
ADAM10	*ADAM10*					
TMEM68	*TMEM68*					
A1CF	*A1CF*					
INTS2	*INTS2*					
TMC7	*TMC7*					
PMAIP1	*PMAIP1*					
EIF4G2	*EIF4G2*					
PPP3CB	*PPP3CB*					
VGLL4	*VGLL4*					
RIPPLY3	*RIPPLY3*					
TMEM184B	*TMEM184B*					
KCNK1	*KCNK1*					
EYA3	*EYA3*					
MTPN	*MTPN*					
DCBLD2	*DCBLD2*					
TAF9B	*TAF9B*					
TMEM86A	*TMEM86A*					
PFKFB2	*PFKFB2*					
SKP2	*SKP2*					
PAX5	*PAX5*					
SLC2A3	*SLC2A3*					
PHTF2	*PHTF2*					
DCAF7	*DCAF7*					
MAP3K2	*MAP3K2*					
SLC12A2	*SLC12A2*					
GALNT10	*GALNT10*					
SLC2A13	*SLC2A13*					
CSNK1G1	*CSNK1G1*					
ATPAF1	*ATPAF1*					
MFAP3	*MFAP3*					
KCNE4	*KCNE4*					
SLC4A4	*SLC4A4*					
PSD3	*PSD3*					
RTF1	*RTF1*					
SLC19A2	*SLC19A2*					
PDHX	*PDHX*					
BRWD1	*BRWD1*					
CHD1	*CHD1*					
HOXD13	*HOXD13*					
CREB1	*CREB1*					
PYGO1	*PYGO1*					
HOXC9	*HOXC9*					
KLHL18	*KLHL18*					
ENPEP	*ENPEP*					
CREBBP	*CREBBP*					

CDH2	*CDH2*					
CDH4	*CDH4*					
NID1	*NID1*					
TSPYL4	*TSPYL4*					
ZNF275	*ZNF275*					
ARMCX2	*ARMCX2*					
CACNB4	*CACNB4*					
GTF3C2	*GTF3C2*					
FAM49B	*FAM49B*					
SALL1	*SALL1*					
WNK1	*WNK1*					
HECTD4	*HECTD4*					
SLC45A4	*SLC45A4*					
FAXC	*FAXC*					
PLEKHG1	*PLEKHG1*					
GRIN2A	*GRIN2A*					
BCL7B	*BCL7B*					
ARL5B	*ARL5B*					
CCNJ	*CCNJ*					
CREBRF	*CREBRF*					
PHAX	*PHAX*					
GSR	*GSR*					
COMMD8	*COMMD8*					
TNKS2	*TNKS2*					
RFK	*RFK*					
TAOK1	*TAOK1*					
ESR1	*ESR1*					
OAF	*OAF*					
GNPNAT1	*GNPNAT1*					
DFFB	*DFFB*					
EPHA5	*EPHA5*					
MAEA	*MAEA*					
MKNK2	*MKNK2*					
NCEH1	*NCEH1*					
C1GALT1	*C1GALT1*					
RB1	*RB1*					
ZDHHC18	*ZDHHC18*					
ARPC3	*ARPC3*					
LPP	*LPP*					
LEF1	*LEF1*					
MMP16	*MMP16*					
LARP4	*LARP4*					
NACC2	*NACC2*					
CDC6	*CDC6*					
CPM	*CPM*					
ANKIB1	*ANKIB1*					
GRB10	*GRB10*					

HOMER1	*HOMER1*					
HMGA2	*HMGA2*					
SH3PXD2A	*SH3PXD2A*					
YTHDF3	*YTHDF3*					
JARID2	*JARID2*					
ZNF469	*ZNF469*					
PELI2	*PELI2*					
RANBP9	*RANBP9*					
C2CD5	*C2CD5*					
UBR1	*UBR1*					
PGRMC2	*PGRMC2*					
ARHGAP21	*ARHGAP21*					
BHLHE40	*BHLHE40*					
IPPK	*IPPK*					
UBE3A	*UBE3A*					
SOCS6	*SOCS6*					
PAG1	*PAG1*					
ZNF106	*ZNF106*					
TCF12	*TCF12*					
USP37	*USP37*					
PCDH18	*PCDH18*					
EP300	*EP300*					
GOLGA3	*GOLGA3*					
DDX17	*DDX17*					
ZBTB10	*ZBTB10*					
MIB1	*MIB1*					
PRR5L	*PRR5L*					
CDK13	*CDK13*					
KCNQ4	*KCNQ4*					
ACAP2	*ACAP2*					
LSM11	*LSM11*					
GMNC	*GMNC*					
ARHGAP26	*ARHGAP26*					
FAM199X	*FAM199X*					
OTUD1	*OTUD1*					
UBE2E2	*UBE2E2*					
UBE2K	*UBE2K*					
DYRK1A	*DYRK1A*					
RTN1	*RTN1*					
MYCBP	*MYCBP*					
EPHA7	*EPHA7*					
SCML4	*SCML4*					
SLC1A1	*SLC1A1*					
SLC26A4	*SLC26A4*					
SESN3	*SESN3*					
EAF1	*EAF1*					
CSTF2	*CSTF2*					

SBNO1	*SBNO1*					
ZNF385B	*ZNF385B*					
ERLIN1	*ERLIN1*					
CELSR1	*CELSR1*					
CNR1	*CNR1*					
SLC25A20	*SLC25A20*					
SH3RF1	*SH3RF1*					
CELF2	*CELF2*					
USP27X	*USP27X*					
SLC16A6	*SLC16A6*					
KLF4	*KLF4*					
UBE2W	*UBE2W*					
OSBPL2	*OSBPL2*					
EDEM3	*EDEM3*					
SAR1B	*SAR1B*					
SEMA6D	*SEMA6D*					
RAB11A	*RAB11A*					
HIPK1	*HIPK1*					
CHSY1	*CHSY1*					
VDAC1	*VDAC1*					
TSPAN5	*TSPAN5*					
PLXNA2	*PLXNA2*					
TM4SF18	*TM4SF18*					
RDH14	*RDH14*					
KIAA1549L	*KIAA1549L*					
PPP2R5A	*PPP2R5A*					
PPP1R3D	*PPP1R3D*					
PAK1	*PAK1*					
DRAM1	*DRAM1*					
TWF1	*TWF1*					
MINPP1	*MINPP1*					
COL22A1	*COL22A1*					
RAB21	*RAB21*					
MED13L	*MED13L*					
C18orf25	*C18orf25*					
KMT2C	*KMT2C*					
MNX1	*MNX1*					
GTF2A1	*GTF2A1*					
USP25	*USP25*					
RCN1	*RCN1*					
IPMK	*IPMK*					
ZCCHC24	*ZCCHC24*					
RYK	*RYK*					
ACER3	*ACER3*					
EIF5	*EIF5*					
WNT5A	*WNT5A*					
NFE2L3	*NFE2L3*					

LARP4B	*LARP4B*					
COL1A2	*COL1A2*					
CEBPG	*CEBPG*					
CPEB2	*CPEB2*					
TMEM260	*TMEM260*					
CTDSP2	*CTDSP2*					
FAM160A1	*FAM160A1*					
SLC22A23	*SLC22A23*					
YPEL1	*YPEL1*					
KCNH7	*KCNH7*					
LMLN	*LMLN*					
MATR3	*MATR3*					
AKIRIN1	*AKIRIN1*					
WIPF2	*WIPF2*					
CDKN1C	*CDKN1C*					
MFSD6	*MFSD6*					
FUT9	*FUT9*					
TFAP2E	*TFAP2E*					
HSPA13	*HSPA13*					
ZNF516	*ZNF516*					
PPM1H	*PPM1H*					
PLCL1	*PLCL1*					
METAP2	*METAP2*					
ETF1	*ETF1*					
ZEB2	*ZEB2*					
DIDO1	*DIDO1*					
DNAJB5	*DNAJB5*					
GALNT7	*GALNT7*					
TMEM248	*TMEM248*					
TBL1XR1	*TBL1XR1*					
SLC35E2B	*SLC35E2B*					
USP3	*USP3*					
UBE2D1	*UBE2D1*					
SOSTDC1	*SOSTDC1*					
B4GALT1	*B4GALT1*					
MLLT3	*MLLT3*					
CAPZA1	*CAPZA1*					
IPO7	*IPO7*					
LINGO1	*LINGO1*					
REEP4	*REEP4*					
PDE7A	*PDE7A*					
ARL4C	*ARL4C*					
RAB31	*RAB31*					
PDE4D	*PDE4D*					
PURA	*PURA*					
CCDC50	*CCDC50*					
FAM172A	*FAM172A*					

FBXO42	*FBXO42*					
HECTD3	*HECTD3*					
NEK6	*NEK6*					
CCND2	*CCND2*					
ADAM12	*ADAM12*					
RAP1A	*RAP1A*					
ST8SIA4	*ST8SIA4*					
PPP1R15B	*PPP1R15B*					
ZNRF3	*ZNRF3*					
GPC4	*GPC4*					
RFX7	*RFX7*					
ROCK1	*ROCK1*					
TMEM135	*TMEM135*					
B4GALT4	*B4GALT4*					
NAGPA	*NAGPA*					
ATXN7	*ATXN7*					
PNRC1	*PNRC1*					
LPAR3	*LPAR3*					
CARM1	*CARM1*					
TMEM33	*TMEM33*					
GATA4	*GATA4*					
UBTD2	*UBTD2*					
PFDN4	*PFDN4*					
FAM118B	*FAM118B*					
TMEM64	*TMEM64*					
NCAM2	*NCAM2*					
RANBP10	*RANBP10*					
CTNNBIP1	*CTNNBIP1*					
MSTN	*MSTN*					
HAPLN1	*HAPLN1*					
COL11A1	*COL11A1*					
DAB2	*DAB2*					
KCTD18	*KCTD18*					
DERL2	*DERL2*					
GRSF1	*GRSF1*					
UBE2J1	*UBE2J1*					
MYPOP	*MYPOP*					
TMEM178B	*TMEM178B*					
RAP1B	*RAP1B*					
ARL6IP6	*ARL6IP6*					
RP2	*RP2*					
DMXL1	*DMXL1*					
LMAN1	*LMAN1*					
ZFHX3	*ZFHX3*					
LTBP1	*LTBP1*					
TRAF6						
IRAK1						

DCAF12						
IGSF1						
HIPK3						
LCOR						
KLF7						
ZBTB2						
ZNF367						
SIAH2						
CD80						
SLC10A3						
HNRNPD						
ZDHHC13						
FBXW2						
ERBB4						
BCORL1						
MYBL1						
SEC23IP						
WWC2						
RARB						
APPL1						
NUMB						
CDKN2AIP						
ZNF652						
LRP2						
ROBO1						
DDHD1						
C16orf72						
SYT1						
NOVA1						
ZNF512B						
TMEM120B						
PIP5K1B						
LFNG						
PPP1R11						
ZNF532						
AAK1						
ACKR2						
RIMS2						
LRRC15						
XKR4						
CDS1						
VASN						
RABGAP1						
TBC1D20						
PTGFRN						
SORT1						
GDNF						

SCN3B						
ARMC8						
ESYT2						
CARD10						
RPA3						
ZFYVE1						
BMPR1A						
PRKAA2						
SRSF12						
MYT1						
PRX						
ZNRF2						
SEMA3G						
PTPRA						
GRID1						
MAPRE2						
PLCD1						
TMOD2						
NRCAM						
AMMECR1						
TJP1						
ZCCHC14						
ZBTB34						
SATB1						
NEURL4						
MAPRE3						
WIZ						
BDNF						
TAF5						
PTGER4						
CREB3L3						
MYCBP2						
AVL9						
SORBS2						
SLC20A2						
PCDH19						
HIVEP2						
HK2						
DACH1						
LAT						
MEN1						
SNRNP200						
C17orf112						
CYB561D1						
TRAT1						
MGAT5						
FGF1						

TSGA10						
ANAPC7						
KIAA1549						
IL21R						
APLN						
XPO4						
ADAM33						
TOPBP1						
S100PBP						
ACVR1B						
IFNAR1						
MTF2						
SERTAD1						
ZNF37A						
FOSL2						
KMT2A						
PLEKHG4						
NOL9						
XKR9						
LRPAP1						
STRN						
NRG2						
LAMB3						
PTPRE						
CACTIN						
WFS1						
ORAI2						
CSRNP2						
KCNJ14						
CRTC3						
IL20RB						
EMX1						
GRM1						
RNF24						
PRTG						
TPRX1						
MMP14						
CRTC2						
RHOBTB1						
APBA1						
PIKFYVE						
PCDH7						
CDK9						
MBD1						
BSDC1						
ABR						
TCHP						

DBNDD2						
POT1						
PIK3CD						
GPR63						
CPLX3						
SORBS1						
ATP6V0A2						
MED8						
NCR3						
UAP1L1						
AMPD2						
CYB5RL						
RNF8						
DAG1						
LAIR1						
PRM1						
WWC1						
SPSB4						
TPT1						
DISC1						
PYGO2						
CTAGE1						
LPL						
ZNF667						
C22orf46						
THBD						
GCSAM						
FAM71E2						
SAMD4B						
FRMD5						
TNFSF9						
TSTD2						
FLOT1						
CRTAP						
DDX6						
STARD3NL						
UBE2R2						
CSF1						
CACHD1						
MBNL3						
ENY2						
IGBP1						
HLA-DOB						
PKNOX1						
KSR2						
RABGAP1L						
GJC1						

SMARCA2						
TK2						
PDGFA						
LMAN2L						
SOX5						
KCNC1						
ABLIM2						
ZMIZ1						
HHIPL2						
PITPNA						
SLC43A2						
TEX261						
THPO						
PRR14L						
SCP2						
CCNL1						
ST6GAL1						
HNF1A						
SLC17A9						
CLOCK						
NCAN						
PPP6R3						
TSPAN17						
SLC6A1						
ALPK3						
CALM1						
FMNL3						
CCR9						
MPRIP						
LRTM2						
MCTP1						
GLDN						
PAK3						
FNDC5						
MAP4K5						
ATP1B2						
ANO2						
WNT2B						
NFAM1						
MAPKAPK2						
DLG2						
SMIM7						
DCHS1						
TNR						
FAM178B						
PDE1C						
STC1						

TRPS1						
PEX16						
ZNF25						
PEA15						
TAF11						
CYB5B						
LTBP2						
LYSMD1						
VKORC1						
ZNF24						
ARF4						
ELMO2						
TBC1D24						
ZNF581						
MLXIP						
GPR155						
ABCB10						
PRRT2						
DPYSL2						
ABHD2						
TMEM184A						
LMX1B						
RAP2A						
TOX2						
TXNRD3						
PEX6						
UBR2						
WDR3						
GRPEL2						
DAGLA						
ALS2CL						
IPO11						
VIPR1						
KLHDC3						
RIMS3						
IL6R						
DNAJC5G						
EMC6						
B3GNT9						
EFR3B						
ETS1						
NUP153						
HOXA11						
SPOCK2						
KHK						
HYDIN						
SAMD5						

DENND5A						
STX5						
C9orf62						
PTPN14						
C1QTNF7						
NR6A1						
SNX17						
C3orf62						
OGDH						
CNNM1						
CDYL2						
TGM2						
COLQ						
MINK1						
NFATC4						
SP6						
PROM2						
GPX1						
CEP170B						
LRRC32						
TRIM66						
TINF2						
NAT8L						
EIF4A2						
CARHSP1						
STEAP3						
NEO1						
TRIM74						
HS3ST3B1						
C7orf43						
TRIM67						
IRGQ						
SUSD2						
DCLK3						
CCNG1						
CLEC6A						
PPME1						
GATAD2B						
ATP7B						
EME1						
KIAA0513						
BACE1						
STRN3						
SNCB						
MAP2K6						
EGFLAM						
IRAK2						

ZFPM2						
CAMK4						
KIF13A						
ALAS1						
ZSCAN29						
EXOC8						
DNAJA1						
GALNT1						
RBM12B						
CPEB4						
RBM27						
GSE1						
ZC3H6						
PRPF4						
HMGB3						
ABHD17B						
CYP3A7						
ZBTB44						
DNAJA2						
RUNDC3B						
ZC3H12B						
PPP6C						
TGFBR1						
LHX8						
CNTN4						
LEPROTL1						
FLG2						
OSBPL8						
TWISTNB						
RBFOX2						
ATF7IP						
FAM151B						
LGR5						
NEUROD1						
FAM19A1						
MAD2L1						
COL4A3BP						
ECT2L						
SLC39A10						
NDUFAF5						
WDR76						
HOOK3						
ASIC5						
GGPS1						
SMC6						
ATRNL1						
B3GNT5						

FOXN2						
HOXA7						
HIF1A						
IAPP						
STARD4						
SOX11						
TNFSF11						
ANKRD20A3						
ATG3						
ESCO1						
TMEFF2						
DESI2						
ATRN						
ELL2						
KLHL4						
FMNL2						
ANKRD20A4						
ETV1						
RBM17						
DPY19L2						
FREM2						
SMIM15						

Table S6a. KEGG enrichment analysis. KEGG: Kyoto Encyclopedia of Genes and Genomes.

ID	Description	GeneRatio	BgRatio	pvalue	p.adjust	qvalue	geneID	Count
hsa04360	Axon guidance	22/387	181/8031	5.40882E-05	0.015306972	0.012981177	*GSK3B/EPHA2/SRGAP1/NTN4/PAK2/PPP3CB/EPHA5/EPHA7/SEMA6D/PLXNA2/PAK1/RYK/WNT5A/ROCK1/ROBO1/SEMA3G/PIK3CD/ABLIM2/PAK3/DPYSL2/NFATC4/NEO1*	22
hsa04310	Wnt signaling pathway	19/387	160/8031	0.000236022	0.029177287	0.024743989	*GSK3B/PLCB1/FRAT2/VANGL2/NLK/PPP3CB/CREBBP/LEF1/EP300/RYK/WNT5A/TBL1XR1/CCND2/ZNRF3/GPC4/CTNNBIP1/WNT2B/NFATC4/LGR5*	19
hsa04720	Long-term potentiation	11/387	67/8031	0.000316838	0.029177287	0.024743989	*PLCB1/RPS6KA6/PPP3CB/CREBBP/GRIN2A/EP300/RAP1A/RAP1B/GRM1/CALM1/CAMK4*	11
hsa05211	Renal cell carcinoma	11/387	69/8031	0.0004124	0.029177287	0.024743989	*HGF/PAK2/CREBBP/EP300/PAK1/RAP1A/RAP1B/PIK3CD/PAK3/ETS1/HIF1A*	11
hsa04010	MAPK signaling pathway	27/387	294/8031	0.000912112	0.051625519	0.043781358	*HGF/MRAS/EPHA2/ATF2/RPS6KA6/NLK/DUSP5/PAK2/HSPA8/PPP3CB/MAP3K2/CACNB4/TAOK1/MKNK2/PAK1/RAP1A/RAP1B/TRAF6/IRAK1/ERBB4/BDNF/FGF1/CSF1/PDGFA/MAPKAPK2/MAP2K6/TGFBR1*	27
hsa04218	Cellular senescence	17/387	156/8031	0.001325222	0.054343312	0.046086201	*PTEN/MRAS/CDK6/PPP3CB/RB1/HIPK1/VDAC1/CCND2/GATA4/HIPK3/PIK3CD/CALM1/MAPKAPK2/ETS1/NFATC4/MAP2K6/TGFBR1*	17
hsa00514	Other types of O-glycan biosynthesis	8/387	47/8031	0.001626778	0.054343312	0.046086201	*ST6GAL2/GALNT10/C1GALT1/GALNT7/B4GALT1/LFNG/ST6GAL1/GALNT1*	8
hsa04722	Neurotrophin signaling pathway	14/387	119/8031	0.001676264	0.054343312	0.046086201	*PRKCD/GSK3B/RPS6KA6/RAP1A/RAP1B/TRAF6/IRAK1/SORT1/BDNF/PIK3CD/CALM1/MAPKAPK2/IRAK2/CAMK4*	14
hsa04919	Thyroid hormone signaling pathway	14/387	121/8031	0.001966654	0.054343312	0.046086201	*GSK3B/PLCB1/ATP1A2/TBC1D4/PFKFB2/CREBBP/ESR1/EP300/MED13L/GATA4/PLCD1/PIK3CD/ATP1B2/HIF1A*	14
hsa04931	Insulin resistance	13/387	108/8031	0.001973962	0.054343312	0.046086201	*PRKCD/GSK3B/PTEN/PRKCQ/RPS6KA6/TBC1D4/CREB1/PPP1R3D/PRKAA2/CREB3L3/CRTC2/PIK3CD/MLXIP*	13
hsa00562	Inositol phosphate metabolism	10/387	72/8031	0.002218017	0.054343312	0.046086201	*PTEN/PLCB1/MTM1/IPPK/MINPP1/IPMK/PIP5K1B/PLCD1/PIKFYVE/PIK3CD*	10

hsa04070	Phosphatidylinositol signaling system	12/387	97/8031	0.00230431	0.054343312	0.046086201	*PTEN/PLCB1/MTM1/DGKH/IPPK/IPMK/PIP5K1B/CDS1/PLCD1/PIKFYVE/PIK3CD/CALM1*	12
hsa04918	Thyroid hormone synthesis	10/387	75/8031	0.003016701	0.065671256	0.055692938	*PLCB1/ATP1A2/ATF2/CREB1/GSR/SLC26A4/LRP2/CREB3L3/ATP1B2/GPX1*	10
hsa05167	Kaposi sarcoma-associated herpesvirus infection	18/387	189/8031	0.004326545	0.087458025	0.07416935	*GSK3B/CDK6/PTGS2/BAK1/BID/PPP3CB/CREB1/CREBBP/RB1/EP300/IFNAR1/PIK3CD/CALM1/MAPKAPK2/NFATC4/MAP2K6/HIF1A/ATG3*	18
hsa05165	Human papillomavirus infection	27/387	330/8031	0.004762917	0.089860375	0.076206679	*GSK3B/PTEN/ITGA5/CDK6/PTGS2/BAK1/JAG1/ITGB8/CREB1/CREBBP/RB1/UBE3A/EP300/PPP2R5A/WNT5A/COL1A2/CCND2/LFNG/PTGER4/CREB3L3/IFNAR1/LAMB3/PIK3CD/ATP6V0A2/WNT2B/DLG2/TNR*	27
hsa04350	TGF-beta signaling pathway	11/387	94/8031	0.005323429	0.094158158	0.079851441	*SMAD1/ACVR1C/INHBB/CREBBP/EP300/ROCK1/LTBP1/BMPR1A/ACVR1B/NEO1/TGFBR1*	11
hsa04140	Autophagy - animal	14/387	137/8031	0.006130654	0.102057361	0.086550412	*PRKCD/ULK2/PTEN/MRAS/PRKCQ/ULK1/EIF2S1/TRAF6/ZFYVE1/PRKAA2/PIK3CD/IGBP1/HIF1A/ATG3*	14
hsa04141	Protein processing in endoplasmic reticulum	16/387	167/8031	0.006564517	0.103208793	0.087526891	*UBE4B/UBE2G1/EIF2S1/BAK1/HSPA8/HSPA4L/EDEM3/SAR1B/UBE2D1/DERL2/UBE2J1/LMAN1/WFS1/DNAJC5G/DNAJA1/DNAJA2*	16
hsa04120	Ubiquitin mediated proteolysis	14/387	140/8031	0.007401092	0.108073281	0.091652252	*UBE4B/UBE2H/UBE2G1/SKP2/UBE3A/UBE2E2/UBE2K/UBE2W/UBE2D1/UBE2J1/TRAF6/ANAPC7/RHOBTB1/UBE2R2*	14
hsa04934	Cushing syndrome	15/387	155/8031	0.007637688	0.108073281	0.091652252	*GSK3B/PLCB1/ATF2/CDK6/CREB1/NCEH1/RB1/LEF1/WNT5A/RAP1A/RAP1B/CREB3L3/MEN1/KMT2A/WNT2B*	15
hsa04510	Focal adhesion	18/387	201/8031	0.00814276	0.109649968	0.092989372	*HGF/GSK3B/PTEN/ITGA5/ITGB8/PAK2/PAK1/COL1A2/CCND2/RAP1A/ROCK1/RAP1B/PIP5K1B/LAMB3/PIK3CD/PDGFA/PAK3/TNR*	18
hsa05030	Cocaine addiction	7/387	49/8031	0.008524026	0.109649968	0.092989372	*ATF2/GPSM1/DLG4/CREB1/GRIN2A/BDNF/CREB3L3*	7
hsa05166	Human T-cell leukemia virus 1 infection	19/387	219/8031	0.009239073	0.113680768	0.096407719	*PTEN/ATF2/PPP3CB/CREB1/CREBBP/RB1/EP300/VDAC1/CCND2/CREB3L3/ANAPC7/CRTC3/CRTC2/PIK3CD/HLA-DOB/ETS1/NFATC4/*	19

							TGFBR1/MAD2L1	

Table S6b. GO_BP enrichment analysis. GO: Gene Ontology. BP: Biological Process.

ID	Description	GeneRatio	BgRatio	pvalue	p.adjust	qvalue	geneID	Count
GO:0007409	axonogenesis	51/880	468/18670	2.25841E-08	6.91416E-05	5.88337E-05	*GSK3B/ULK2/PTEN/RNF6/EPHA2/ZSWIM6/VANGL2/PRKCQ/ULK1/NTN4/MAP2/USP9X/PPP3CB/CREB1/CDH2/CDH4/EPHA5/GRB10/RANBP9/EPHA7/SEMA6D/RAB11A/PLXNA2/PAK1/RAB21/RYK/WNT5A/ZEB2/LINGO1/LPAR3/KLF7/NUMB/ROBO1/GDNF/SEMA3G/PTPRA/NRCAM/BDNF/MYCBP2/PIK3CD/DAG1/DISC1/FLOT1/PITPNA/LRTM2/PAK3/TNR/DPYSL2/NEO1/CNTN4/ETV1*	51
GO:0050770	regulation of axonogenesis	28/880	183/18670	3.61209E-08	6.91416E-05	5.88337E-05	*GSK3B/ULK2/PTEN/RNF6/ZSWIM6/MAP2/CDH2/CDH4/EPHA7/SEMA6D/RAB11A/PLXNA2/PAK1/RAB21/RYK/WNT5A/ZEB2/LINGO1/LPAR3/ROBO1/SEMA3G/NRCAM/BDNF/MYCBP2/DISC1/PAK3/TNR/DPYSL2*	28
GO:0048638	regulation of developmental growth	41/880	347/18670	6.06466E-08	6.91416E-05	5.88337E-05	*GSK3B/ULK2/PTEN/RNF6/PLCB1/MTM1/FLVCR1/MAP2/RGS4/PIM1/CREB1/CDH4/TNKS2/JARID2/EPHA7/SEMA6D/RAB11A/PAK1/RAB21/RYK/WNT5A/LPAR3/CARM1/MSTN/ERBB4/WWC2/SYT1/RIMS2/BMPR1A/SEMA3G/NRCAM/BDNF/WWC1/DISC1/CSF1/TNR/DPYSL2/COLQ/ZFPM2/TGFBR1/ATRN*	41
GO:0046777	protein autophosphorylation	32/880	235/18670	6.69847E-08	6.91416E-05	5.88337E-05	*STK39/GSK3B/ULK2/ULK1/NLK/MEX3B/ABL2/WNK3/EIF2S1/MAP3K9/PIM1/RAP2C/BCR/PAK2/WNK1/TAOK1/MKNK2/DYRK1A/EPHA7/PPP2R5A/PAK1/NEK6/IRAK1/ERBB4/AAK1/ACVR1B/PDGFA/CALM1/MAPKAPK2/RAP2A/MINK1/CAMK4*	32
GO:0060560	developmental growth involved in morphogenesis	32/880	235/18670	6.69847E-08	6.91416E-05	5.88337E-05	*GSK3B/ULK2/RNF6/VANGL2/ULK1/MAP2/USP9X/PPP3CB/HOXD13/CDH4/SALL1/ESR1/EPHA7/SEMA6D/RAB11A/PAK1/RAB21/RYK/WNT5A/ZEB2/LPAR3/SYT1/RIMS2/SEMA3G/NRCAM/BDNF/FGF1/EMX1/DISC1/CSF1/TNR/DPYSL2*	32
GO:0061138	morphogenesis of a branching	27/880	182/18670	1.21273E-07	0.000104315	8.87636E-05	*HGF/SULF1/EPHA2/VANGL2/HOXA5/ADM/NTN4/BTBD7/HOXD13/*	27

	epithelium						*SALL1/ESR1/LEF1/CELSR1/PAK1/WNT5A/CTNNBIP1/GDNF/FGF1/MMP14/DAG1/CSF1/PDGFA/WNT2B/DCHS1/HOXA11/TGM2/NFATC4*	
GO:0007178	transmembrane receptor protein serine/threonine kinase signaling pathway	39/880	349/18670	5.43739E-07	0.000311804	0.000265319	*SMAD1/SULF1/TOB1/ACVR1C/CILP/USP15/ADAM17/HPGD/NLK/ASPN/INHBB/USP9X/SLC33A1/CREB1/CREBBP/LEF1/EP300/WNT5A/COL1A2/CDKN1C/UBE2D1/SOSTDC1/GATA4/MSTN/DAB2/LTBP1/IRAK1/IGSF1/APPL1/LRP2/VASN/BMPR1A/MEN1/ACVR1B/LTBP2/LRRC32/NEO1/TGFBR1/SOX11*	39
GO:0061387	regulation of extent of cell growth	19/880	110/18670	8.53986E-07	0.000440742	0.000375035	*GSK3B/ULK2/RNF6/MAP2/CDH4/EPHA7/SEMA6D/RAB11A/PAK1/RAB21/RYK/WNT5A/LPAR3/SEMA3G/NRCAM/BDNF/DISC1/TNR/DPYSL2*	19
GO:0048588	developmental cell growth	29/880	234/18670	1.97392E-06	0.000926126	0.000788056	*GSK3B/ULK2/RNF6/ULK1/MAP2/USP9X/RGS4/PPP3CB/CDH4/EPHA7/SEMA6D/RAB11A/PAK1/RAB21/RYK/WNT5A/ZEB2/LPAR3/GATA4/SYT1/RIMS2/SEMA3G/NRCAM/BDNF/SORBS2/EMX1/DISC1/TNR/DPYSL2*	29
GO:0016049	cell growth	47/880	484/18670	2.26271E-06	0.000973153	0.000828072	*GSK3B/ULK2/RNF6/ADAM17/PRKCQ/DCUN1D3/ULK1/DDX3X/MAP2/USP9X/RGS4/ADAM10/EIF4G2/PPP3CB/MTPN/DCBLD2/TAF9B/CDH4/RB1/LEF1/EPHA7/SEMA6D/RAB11A/PAK1/RAB21/RYK/WNT5A/ZEB2/LPAR3/GATA4/DERL2/CDKN2AIP/SYT1/RIMS2/SEMA3G/NRCAM/BDNF/SORBS2/ACVR1B/EMX1/MMP14/TCHP/DISC1/SMARCA2/TNR/DPYSL2/TGFBR1*	47
GO:0071559	response to transforming growth factor beta	30/880	255/18670	3.88228E-06	0.001178613	0.001002901	*SMAD1/ACVR1C/CILP/USP15/ADAM17/HPGD/NLK/ASPN/USP9X/CREB1/CREBBP/EP300/WNT5A/COL1A2/CDKN1C/ROCK1/MSTN/DAB2/ZFHX3/LTBP1/APPL1/VASN/BMPR1A/MEN1/ACVR1B/SOX5/LTBP2/LRRC32/TGFBR1/SOX11*	30
GO:0022604	regulation of cell morphogenesis	46/880	484/18670	5.14452E-06	0.001475047	0.001255142	*GSK3B/FGD1/ULK2/PTEN/RNF6/ZSWIM6/PTPRD/RHOQ/CAMSAP1/MAP2/RHOU/SH3D19/DLG4/ADAM10/BRWD1/CDH2/CDH4/LARP4/UBE3A/EPHA7/SEMA6D/RAB11A/*	46

							PLXNA2/PAK1/RAB21/RYK/WNT5A/ZEB2/LINGO1/LPAR3/ROBO1/SYT1/RIMS2/SEMA3G/NRCAM/BDNF/MYCBP2/DAG1/DISC1/FMNL3/PAK3/TNR/DPYSL2/RAP2A/NFATC4/FMNL2	
GO:0007179	transforming growth factor beta receptor signaling pathway	25/880	199/18670	7.77411E-06	0.00200248	0.001703942	*SMAD1/ACVR1C/USP15/ADAM17/HPGD/NLK/ASPN/USP9X/CREB1/CREBBP/EP300/COL1A2/CDKN1C/MSTN/DAB2/LTBP1/APPL1/VASN/BMPR1A/MEN1/ACVR1B/LTBP2/LRRC32/TGFBR1/SOX11*	25
GO:0071902	positive regulation of protein serine/threonine kinase activity	35/880	334/18670	8.70329E-06	0.002041713	0.001737327	*STK39/HGF/EZH2/PDCD10/VANGL2/ADAM17/DDX3X/MAP3K9/DUSP5/MAP3K2/TAOK1/CDC6/HMGA2/PAK1/WNT5A/ZEB2/CCND2/LPAR3/TRAF6/IRAK1/ROBO1/MAPRE3/FGF1/GRM1/PDGFA/CALM1/PAK3/MAP4K5/MAPKAPK2/PEA15/SAMD5/MAP2K6/IRAK2/TGFBR1/TNFSF11*	35
GO:0008361	regulation of cell size	23/880	179/18670	1.22526E-05	0.002749379	0.002339492	*GSK3B/ULK2/PTEN/RNF6/MAP2/MTPN/SLC12A2/CREB1/CDH4/EPHA7/SEMA6D/RAB11A/PAK1/RAB21/RYK/WNT5A/LPAR3/SEMA3G/NRCAM/BDNF/DISC1/TNR/DPYSL2*	23
GO:0016569	covalent chromatin modification	44/880	474/18670	1.48376E-05	0.002961012	0.002519573	*TET2/PRKCD/TET3/EPC1/EZH2/SRCAP/USP15/ATF2/RCOR1/REST/RLF/TET1/EP400/EYA3/TAF9B/PAX5/RTF1/CHD1/CREBBP/LEF1/NACC2/HMGA2/JARID2/EP300/KMT2C/TBL1XR1/USP3/ATXN7/CARM1/PRKAA2/SATB1/TAF5/MEN1/MTF2/KMT2A/CRTC2/CDK9/MBD1/RNF8/PYGO2/ENY2/CLOCK/UBR2/ATF7IP*	44
GO:0048872	homeostasis of number of cells	28/880	246/18670	1.50769E-05	0.002961012	0.002519573	*SLC7A11/MFHAS1/EZH2/FLVCR1/ADAM17/HOXA5/PTBP3/CDK6/BAK1/BCR/SFXN1/PDE4B/PMAIP1/PPP3CB/CDH2/MAEA/RB1/CEBPG/LAT/ACVR1B/IL20RB/EMX1/PIK3CD/CSF1/PKNOX1/ETS1/RBFOX2/HIF1A*	28
GO:0030099	myeloid cell differentiation	40/880	416/18670	1.60644E-05	0.002961012	0.002519573	*TET2/TNRC6B/MFHAS1/EPHA2/FLVCR1/HOXA5/PRKCQ/TNRC6C/TNRC6A/PTBP3/CDK6/HOXA9/JAG1/SFXN1/CREB1/CREBBP/MAEA/RB1/LEF1/SH3PXD2A/EP300/KM*	40

							T2C/CEBPG/CDKN1C/TMEM64/CTNNBIP1/TRAF6/ACVR1B/KMT2A/PIK3CD/CSF1/PKNOX1/THPO/ETS1/CAMK4/HMGB3/RBFOX2/HOXA7/HIF1A/TNFSF11	
GO:0002065	columnar/cuboidal epithelial cell differentiation	17/880	112/18670	1.90275E-05	0.003125499	0.002659538	GSK3B/HOXA5/CDK6/JAG1/RFX3/CDH2/C1GALT1/LEF1/WNT5A/GATA4/RARB/MEN1/EMX1/CLOCK/NEUROD1/HIF1A/SOX11	17
GO:0014706	striated muscle tissue development	38/880	390/18670	1.93792E-05	0.003125499	0.002659538	RBM24/SMAD1/PTEN/ZBTB18/UBE4B/MTM1/COL19A1/RGS4/CXADR/PIM1/MTPN/PAX5/CREB1/RB1/LEF1/HOMER1/JARID2/EP300/DDX17/PAK1/WNT5A/AKIRIN1/GATA4/MSTN/COL11A1/ERBB4/RARB/LRP2/BMPR1A/SORBS2/FLOT1/GJC1/ALPK3/GPX1/ZFPM2/TGFBR1/FOXN2/SOX11	38
GO:0045637	regulation of myeloid cell differentiation	28/880	251/18670	2.18781E-05	0.003320971	0.002825868	TNRC6B/HOXA5/PRKCQ/TNRC6C/TNRC6A/CDK6/HOXA9/JAG1/CREB1/CREBBP/RB1/LEF1/EP300/KMT2C/TMEM64/CTNNBIP1/TRAF6/ACVR1B/KMT2A/CSF1/THPO/ETS1/CAMK4/HMGB3/RBFOX2/HOXA7/HIF1A/TNFSF11	28
GO:1905475	regulation of protein localization to membrane	23/880	187/18670	2.5039E-05	0.003428353	0.002917242	SLC7A11/ACSL3/EPHA2/RHOQ/STAC2/WNK3/BID/ADAM10/PMAIP1/CDH2/C2CD5/RAB11A/PPP2R5A/PAK1/RAP1A/GPC4/DAB2/APPL1/NUMB/LRRC15/MMP14/SORBS1/DAG1	23
GO:1904375	regulation of protein localization to cell periphery	17/880	115/18670	2.70591E-05	0.003580815	0.003046973	ACSL3/EPHA2/RHOQ/STAC2/WNK3/ADAM10/RAB11A/PPP2R5A/RAP1A/GPC4/DAB2/APPL1/NUMB/LRRC15/MMP14/SORBS1/DAG1	17
GO:1990778	protein localization to cell periphery	32/880	311/18670	3.00601E-05	0.00387851	0.003300287	CLASP2/ACSL3/EPHA2/RHOQ/MAP7/STAC2/WNK3/ADAM10/CDH2/GRIN2A/C2CD5/RAB11A/TSPAN5/PPP2R5A/RAB31/RAP1A/GPC4/ROCK1/DAB2/APPL1/NUMB/LRRC15/SCN3B/MMP14/SORBS1/DAG1/FLOT1/DLG2/DCHS1/RAP2A/EFR3B/KIF13A	32
GO:0043393	regulation of protein binding	25/880	217/18670	3.4697E-05	0.004239199	0.003607204	PRKCD/GSK3B/STYX/GPSM1/EIF2S1/MAP2/BAK1/LEF1/EP300/WNT5A/PLCL1/ROCK1/CARM1/CTNNBIP1/DAB2/LFNG/MAPRE3/BDNF/MEN1/LRPAP1/DISC1/FLOT1/NFATC4/TGFBR1/TNFSF11	25

GO:0051098	regulation of binding	36/880	373/18670	3.89304E-05	0.004239199	0.003607204	*ZNF462/PRKCD/GSK3B/STYX/GPSM1/EIF2S1/MAP2/BAK1/PON1/RB1/LEF1/HMGA2/EP300/KLF4/WNT5A/CEBPG/PLCL1/ROCK1/CARM1/CTNNBIP1/DAB2/TRAF6/LFNG/MAPRE3/BDNF/MEN1/LRPAP1/CDK9/DISC1/PYGO2/FLOT1/NFATC4/TGFBR1/NEUROD1/SOX11/TNFSF11*	36
GO:1990823	response to leukemia inhibitory factor	15/880	96/18670	4.10696E-05	0.004239199	0.003607204	*ADAM23/MRAS/PDCD10/CTH/MAT2A/CREB1/GNPNAT1/JARID2/KLF4/HK2/MTF2/SPOCK2/EIF4A2/TWISTNB/TNFSF11*	15
GO:1990830	cellular response to leukemia inhibitory factor	15/880	96/18670	4.10696E-05	0.004239199	0.003607204	*ADAM23/MRAS/PDCD10/CTH/MAT2A/CREB1/GNPNAT1/JARID2/KLF4/HK2/MTF2/SPOCK2/EIF4A2/TWISTNB/TNFSF11*	15
GO:0050804	modulation of chemical synaptic transmission	40/880	436/18670	4.67593E-05	0.004553299	0.003874476	*SLC7A11/GSK3B/PTEN/PLCB1/ATP1A2/PTGS2/RGS4/BCR/DLG4/AKAP7/PPP3CB/CREB1/CDH2/GRIN2A/HOMER1/CNR1/RAB11A/PLCL1/RAP1A/RAP1B/SYT1/RIMS2/GDNF/PTPRA/GRID1/BDNF/GRM1/APBA1/ABR/CPLX3/DISC1/FLOT1/SLC6A1/MCTP1/TNR/PRRT2/RIMS3/NFATC4/BACE1/CNTN4*	40
GO:0099177	regulation of trans-synaptic signaling	40/880	437/18670	4.92025E-05	0.004702481	0.004001418	*SLC7A11/GSK3B/PTEN/PLCB1/ATP1A2/PTGS2/RGS4/BCR/DLG4/AKAP7/PPP3CB/CREB1/CDH2/GRIN2A/HOMER1/CNR1/RAB11A/PLCL1/RAP1A/RAP1B/SYT1/RIMS2/GDNF/PTPRA/GRID1/BDNF/GRM1/APBA1/ABR/CPLX3/DISC1/FLOT1/SLC6A1/MCTP1/TNR/PRRT2/RIMS3/NFATC4/BACE1/CNTN4*	40
GO:0070936	protein K48-linked ubiquitination	11/880	56/18670	5.08834E-05	0.004730486	0.004025248	*RNF6/BFAR/UBE2H/UBE2G1/SKP2/UBE3A/UBE2E2/UBE2K/UBE2D1/RNF8/UBE2R2*	11
GO:0016570	histone modification	41/880	454/18670	5.38974E-05	0.004795938	0.004080942	*TET2/PRKCD/TET3/EPC1/EZH2/SRCAP/USP15/ATF2/RCOR1/REST/RLF/TET1/EP400/EYA3/TAF9B/PAX5/RTF1/CREBBP/LEF1/NACC2/HMGA2/JARID2/EP300/KMT2C/TBL1XR1/USP3/ATXN7/CARM1/PRKAA2/SATB1/TAF5/MEN1/MTF2/KMT2A/CRTC2/CDK9/RNF8/PYGO2/ENY2/CLOCK/UBR2*	41
GO:0030198	extracellular	35/880	368/18670	6.68778E-05	0.005567041	0.004737086	*SULF1/CLASP2/ADAM19/PLOD2/*	35

	matrix organization						*COL19A1/ITGA5/LOXL2/NTN4/HAS3/COL10A1/ITGB8/ADAM10/NID1/RB1/MMP16/SH3PXD2A/COL22A1/COL1A2/B4GALT1/ADAM12/HAPLN1/COL11A1/LAMB3/MMP14/DAG1/FLOT1/PDGFA/NCAN/TNR/ETS1/SPOCK2/COLQ/EGFLAM/EXOC8/TGFBR1*	
GO:0046323	glucose import	12/880	68/18670	7.09716E-05	0.005814038	0.00494726	*ARPP19/RHOQ/GRB10/C2CD5/RAP1A/APPL1/SORT1/HK2/SORBS1/HNF1A/PEA15/OSBPL8*	12
GO:0045927	positive regulation of growth	28/880	270/18670	8.07771E-05	0.006413704	0.005457525	*PLCB1/MTM1/ADAM17/DDX3X/PIM1/ADAM10/EIF4G2/MTPN/TAF9B/CREB1/CDH4/LEF1/RAB11A/PAK1/LPAR3/DERL2/ERBB4/CDKN2AIP/SYT1/RIMS2/BMPR1A/BDNF/WFS1/MMP14/DISC1/CSF1/ZFPM2/TGFBR1*	28
GO:0051588	regulation of neurotransmitter transport	18/880	139/18670	9.51239E-05	0.006818533	0.005802001	*GSK3B/ATP1A2/RGS4/CNR1/RAP1A/RAP1B/SYT1/RIMS2/GDNF/APLN/APBA1/CPLX3/FLOT1/SLC6A1/MCTP1/RIMS3/BACE1/TNFSF11*	18
GO:0072659	protein localization to plasma membrane	27/880	260/18670	0.000105097	0.007165905	0.006097586	*CLASP2/ACSL3/EPHA2/RHOQ/MAP7/STAC2/WNK3/CDH2/C2CD5/RAB11A/TSPAN5/PPP2R5A/RAB31/RAP1A/ROCK1/DAB2/APPL1/NUMB/LRRC15/SCN3B/MMP14/SORBS1/FLOT1/DCHS1/RAP2A/EFR3B/KIF13A*	27
GO:0010498	proteasomal protein catabolic process	41/880	477/18670	0.000159075	0.010135601	0.008624548	*KLHL42/GSK3B/TRIB2/CHFR/STYX/UBR3/UBE4B/MTM1/FBXL19/BFAR/UBE2H/UBE2G1/PMAIP1/SKP2/MAEA/ANKIB1/UBR1/UBE3A/SOCS6/UBE2K/ERLIN1/UBE2W/EDEM3/USP25/HSPA13/TBL1XR1/UBE2D1/HECTD3/DAB2/DERL2/UBE2J1/SIAH2/ARMC8/ANAPC7/WFS1/SPSB4/UBE2R2/CLOCK/UBR2/GPX1/MAD2L1*	41
GO:0016311	dephosphorylation	41/880	478/18670	0.000166336	0.010469	0.008908243	*MFHAS1/PRKCD/GSK3B/STYX/PTEN/MTM1/CILP/ARPP19/PTPRD/CTTNBP2NL/BOD1/PTPN13/BOD1L2/PFKFB3/DUSP5/PON1/PPP3CB/EYA3/PFKFB2/WNK1/PPP2R5A/PPP1R3D/MINPP1/CTDSP2/PPM1H/PPP1R15B/ROCK1/PPP1R11/ZFYVE1/PTPRA/MGAT5/PTPRE/CSRNP2/IGBP1/PPP6R3/CALM1/DLG2/PTPN14/PPME1/PPP6C/SL*	41

							C39A10	
GO:0000381	regulation of alternative mRNA splicing, via spliceosome	11/880	65/18670	0.000207814	0.012617981	0.010736845	*RBM24/THRAP3/REST/DDX17/DYRK1A/CELF2/FAM172A/SRSF12/MBNL3/RBFOX2/RBM17*	11
GO:0000380	alternative mRNA splicing, via spliceosome	12/880	76/18670	0.000214043	0.012645043	0.010759873	*RBM24/THRAP3/REST/DDX17/CDK13/DYRK1A/CELF2/FAM172A/SRSF12/MBNL3/RBFOX2/RBM17*	12
GO:0099633	protein localization to postsynaptic specialization membrane	5/880	13/18670	0.00021561	0.012645043	0.010759873	*ADAM10/RAP1A/GPC4/DAG1/DLG2*	5
GO:0099645	neurotransmitter receptor localization to postsynaptic specialization membrane	5/880	13/18670	0.00021561	0.012645043	0.010759873	*ADAM10/RAP1A/GPC4/DAG1/DLG2*	5
GO:0071634	regulation of transforming growth factor beta production	8/880	36/18670	0.000219928	0.012727975	0.010830441	*ATF2/CD200/PTGS2/ITGB8/CREB1/LTBP1/LRRC32/HIF1A*	8
GO:0006986	response to unfolded protein	20/880	176/18670	0.000245687	0.013489253	0.011478225	*CHAC1/BFAR/CTH/EIF2S1/BAK1/HSPA8/HSPA4L/CREBRF/EP300/EDEM3/CTDSP2/HSPA13/DNAJB5/PPP1R15B/TMEM33/DERL2/CREB3L3/WFS1/KLHDC3/DNAJA1*	20
GO:0010818	T cell chemotaxis	7/880	28/18670	0.000250192	0.013535143	0.011517274	*STK39/ADAM17/ADAM10/SLC12A2/WNK1/WNT5A/PIK3CD*	7
GO:0050821	protein stabilization	20/880	178/18670	0.000285641	0.01431255	0.012178782	*PRKCD/PTEN/PDCD10/NLK/NAA15/USP9X/PIM1/A1CF/TAF9B/CREB1/EP300/USP27X/AAK1/WIZ/WFS1/FLOT1/CRTAP/ATP1B2/PEX6/ATF7IP*	20
GO:0001503	ossification	35/880	398/18670	0.000307197	0.014875352	0.01265768	*HGF/SMAD1/NAB1/TOB1/EPHA2/CDK6/ASPN/PTGS2/REST/TFAP2A/JAG1/LEF1/MMP16/CHSY1/RDH14/MINPP1/WNT5A/COL1A2/TMEM64/CTNNBIP1/COL11A1/TRAF6/SORT1/BMPR1A/PTGER4/MEN1/SNRNP200/MMP14/CSF1/DCHS1/STC1/IL6R/HIF1A/SOX11/TNFSF11*	35
GO:1902473	regulation of	6/880	21/18670	0.000317858	0.015094724	0.012844347	*ADAM10/WNT5A/RAP1A/GPC4/D*	6

	protein localization to synapse						*AG1/ABHD17B*	
GO:0071604	transforming growth factor beta production	8/880	38/18670	0.000326956	0.015124322	0.012869531	*ATF2/CD200/PTGS2/ITGB8/CREB1/LTBP1/LRRC32/HIF1A*	8
GO:0051271	negative regulation of cellular component movement	34/880	384/18670	0.000328634	0.015124322	0.012869531	*SULF1/PTEN/CLASP2/PLCB1/ACVR1C/PDCD10/SRGAP1/CD200/JAG1/RAP2C/BCR/TP53INP1/KLF4/SEMA6D/RYK/WNT5A/ERBB4/ROBO1/CARD10/BMPR1A/SEMA3G/PTGER4/DACH1/ABR/DAG1/GCSAM/FRMD5/MCTP1/STC1/ABHD2/RAP2A/OSBPL8/HOXA7/TMEFF2*	34
GO:1905330	regulation of morphogenesis of an epithelium	20/880	180/18670	0.000331164	0.015124322	0.012869531	*HGF/SULF1/PTEN/CLASP2/VANGL2/NTN4/BTBD7/HOXD13/ESR1/CELSR1/RYK/WNT5A/MLLT3/ZNRF3/GPC4/DAB2/GDNF/FGF1/PDGFA/WNT2B*	20
GO:1901861	regulation of muscle tissue development	18/880	155/18670	0.000378067	0.016260044	0.013835937	*RBM24/PTEN/MTM1/RGS4/PIM1/MTPN/CREB1/LEF1/JARID2/DDX17/PAK1/AKIRIN1/MSTN/ERBB4/BMPR1A/FLOT1/ZFPM2/TGFBR1*	18
GO:0043401	steroid hormone mediated signaling pathway	20/880	184/18670	0.000441528	0.018229791	0.015512026	*RNF6/SKP2/CREBRF/ESR1/RB1/LEF1/PGRMC2/UBE3A/EP300/DDX17/PAK1/CARM1/DAB2/RARB/CLOCK/ABHD2/NR6A1/STRN3/DNAJA1/RBFOX2*	20
GO:0032868	response to insulin	26/880	272/18670	0.000514203	0.020258042	0.017237898	*PRKCD/GSK3B/PTEN/ACVR1C/RHOQ/ADM/PRKCQ/TBC1D4/INHBB/GRB10/C2CD5/ZNF106/SESN3/PAK1/CPEB2/RAB31/MSTN/APPL1/SORT1/PTPRA/PTPRE/SORBS1/ATP6V0A2/LPL/KHK/OSBPL8*	26
GO:1904659	glucose transmembrane transport	14/880	108/18670	0.000546013	0.021029648	0.01789447	*ARPP19/RHOQ/SLC2A14/SLC2A3/GRB10/C2CD5/RAP1A/APPL1/SORT1/HK2/SORBS1/HNF1A/PEA15/OSBPL8*	14
GO:0040013	negative regulation of locomotion	34/880	396/18670	0.00057247	0.021565816	0.018350704	*SULF1/PTEN/CLASP2/PLCB1/ACVR1C/PDCD10/SRGAP1/CD200/JAG1/RAP2C/BCR/TP53INP1/KLF4/SEMA6D/RYK/WNT5A/ERBB4/ROBO1/CARD10/BMPR1A/SEMA3G/PTGER4/DACH1/ABR/DAG1/GCSAM/FRMD5/MCTP1/STC1/ABHD2/RAP2A/OSBPL8/HOXA7/TMEFF2*	34
GO:0032869	cellular response to	22/880	216/18670	0.000578179	0.021574005	0.018357672	*PRKCD/GSK3B/PTEN/RHOQ/PRKCQ/TBC1D4/INHBB/GRB10/C2CD*	22

	insulin stimulus						*5/ZNF106/SESN3/PAK1/CPEB2/RAB31/MSTN/APPL1/PTPRA/PTPRE/SORBS1/ATP6V0A2/LPL/OSBPL8*	
GO:1902806	regulation of cell cycle G1/S phase transition	21/880	202/18670	0.000583614	0.021574005	0.018357672	*E2F7/PTEN/EZH2/PLCB1/ADAM17/DCUN1D3/DDX3X/CDK6/BID/RB1/NACC2/CDC6/EP300/LSM11/UBE2E2/KLF4/CTDSP2/CCND2/CARM1/MEN1/SUSD2*	21
GO:0010820	positive regulation of T cell chemotaxis	5/880	16/18670	0.000650137	0.023353374	0.019871766	*STK39/ADAM17/ADAM10/WNK1/WNT5A*	5
GO:0009408	response to heat	19/880	176/18670	0.000660646	0.023353374	0.019871766	*GSK3B/CHORDC1/POM121C/PTGS2/EIF2S1/NUP50/HSPA8/TP53INP1/CREBBP/EP300/HSPA13/MSTN/IRAK1/RPA3/FGF1/MAPKAPK2/NUP153/DNAJA1/DNAJA2*	19
GO:0006509	membrane protein ectodomain proteolysis	8/880	42/18670	0.000667991	0.023452383	0.019956015	*ADAM19/ADAM17/PRKCQ/SH3D19/ADAM10/ROCK1/DAG1/BACE1*	8
GO:0035418	protein localization to synapse	10/880	63/18670	0.000678327	0.023634766	0.020111207	*DLG4/ADAM10/GRIN2A/RAB11A/WNT5A/RAP1A/GPC4/DAG1/DLG2/ABHD17B*	10
GO:0099175	regulation of postsynapse organization	13/880	98/18670	0.000682345	0.023634766	0.020111207	*SLC7A11/PTEN/PTPRD/ADAM10/CDH2/HOMER1/UBE3A/EPHA7/WNT5A/NRCAM/PAK3/ARF4/ABHD17B*	13
GO:0045926	negative regulation of growth	24/880	249/18670	0.000737888	0.025173714	0.021420723	*ULK2/PTEN/RNF6/DCUN1D3/DDX3X/MAP2/RGS4/DCBLD2/JARID2/EPHA7/SEMA6D/PAK1/RYK/WNT5A/MSTN/WWC2/CDKN2AIP/SEMA3G/ACVR1B/TCHP/WWC1/SMARCA2/TNR/HIF1A*	24
GO:0009100	glycoprotein metabolic process	35/880	419/18670	0.000783959	0.025857502	0.02200257	*TET2/TET3/ST6GAL2/SULF1/PLCB1/VANGL2/NUS1/PAWR/TET1/GALNT10/C1GALT1/EDEM3/CHSY1/FUT9/GALNT7/B4GALT1/ST8SIA4/B4GALT4/NAGPA/COL11A1/DERL2/UBE2J1/LMAN1/LFNG/MGAT5/ST6GAL1/NCAN/B3GNT9/SPOCK2/HS3ST3B1/EGFLAM/GALNT1/B3GNT5/HIF1A/DPY19L2*	35
GO:0098693	regulation of synaptic vesicle cycle	14/880	112/18670	0.000789285	0.025857502	0.02200257	*GSK3B/PTEN/PPP3CB/CDH2/CNR1/RAP1A/ROCK1/RAP1B/SYT1/RIMS2/APBA1/CPLX3/RIMS3/BACE1*	14
GO:0048021	regulation of melanin	4/880	10/18670	0.000819292	0.025940881	0.022073518	*SLC7A11/WNT5A/ZEB2/APPL1*	4

	biosynthetic process							
GO:0060068	vagina development	4/880	10/18670	0.000819292	0.025940881	0.022073518	*BAK1/ESR1/WNT5A/LRP2*	4
GO:0098917	retrograde trans-synaptic signaling	4/880	10/18670	0.000819292	0.025940881	0.022073518	*PLCB1/CNR1/DAG1/DAGLA*	4
GO:0035304	regulation of protein dephosphorylation	16/880	139/18670	0.000861213	0.026348126	0.02242005	*MFHAS1/PRKCD/GSK3B/ARPP19/BOD1/BOD1L2/PPP2R5A/PPP1R15B/ROCK1/PPP1R11/MGAT5/IGBP1/PPP6R3/CALM1/PPME1/SLC39A10*	16
GO:0048706	embryonic skeletal system development	15/880	126/18670	0.000871768	0.026348126	0.02242005	*SULF1/FLVCR1/HOXA5/HOXA9/TFAP2A/PAX5/HOXC9/MMP16/WNT5A/COL11A1/MMP14/HOXA11/TGFBR1/HOXA7/SOX11*	15
GO:2000679	positive regulation of transcription regulatory region DNA binding	6/880	25/18670	0.000882732	0.026348126	0.02242005	*RB1/HMGA2/EP300/KLF4/TRAF6/NEUROD1*	6
GO:0031128	developmental induction	7/880	34/18670	0.000888311	0.026348126	0.02242005	*SALL1/HIPK1/ROBO1/GDNF/FGF1/WNT2B/HOXA11*	7
GO:0044843	cell cycle G1/S phase transition	27/880	298/18670	0.000921849	0.027186653	0.023133566	*E2F7/PTEN/EZH2/PLCB1/ADAM17/DCUN1D3/DDX3X/CDK6/RHOU/BID/SKP2/RB1/NACC2/CDC6/USP37/EP300/LSM11/UBE2E2/KLF4/CTDSP2/CCND2/CARM1/RPA3/MEN1/ACVR1B/SUSD2/PPP6C*	27
GO:0031331	positive regulation of cellular catabolic process	31/880	361/18670	0.000979483	0.028085902	0.023898751	*TNRC6B/PRKCD/RBM24/LARP1/GSK3B/TRIB2/CHFR/PTEN/TOB1/MTDH/TNRC6C/TNRC6A/ULK1/GPSM1/PFKFB3/SH3D19/TP53INP1/PFKFB2/ANKIB1/YTHDF3/PRR5L/SESN3/ROCK1/DAB2/HNRNPD/LRP2/PRKAA2/HK2/DISC1/TRIM67/HIF1A*	31
GO:0031652	positive regulation of heat generation	4/880	11/18670	0.00123959	0.0315149	0.026816541	*PTGS2/CNR1/APLN/TNFSF11*	4
GO:0050919	negative chemotaxis	8/880	46/18670	0.00125062	0.03163945	0.026922523	*EPHA7/SEMA6D/RYK/WNT5A/ROBO1/SEMA3G/PDGFA/LRTM2*	8
GO:0031346	positive regulation of cell projection organization	32/880	383/18670	0.001285607	0.032365946	0.02754071	*FAM98A/HGF/EZH2/PTPRD/RHOQ/MAPK6/ABL2/TMEM106B/DLG4/CDH4/EP300/CNR1/RAB11A/PLXNA2/PAK1/TWF1/RAB21/WNT5A/*	32

							AKIRIN1/ZEB2/RAP1A/LPAR3/MSTN/ROBO1/SYT1/RIMS2/BDNF/DISC1/PAK3/PROM2/TRIM67/TGFBR1	
GO:0051090	regulation of DNA-binding transcription factor activity	35/880	432/18670	0.001334547	0.033113444	0.028176769	*PTEN/EZH2/MTDH/ATF2/G3BP2/PRKCQ/CD200/CTH/MXI1/CREBZF/PIM1/MTPN/ESR1/EPHA5/RB1/BHLHE40/EP300/KLF4/WNT5A/CEBPG/CARM1/CTNNBIP1/TRAF6/IRAK1/MEN1/CACTIN/WFS1/CRTC3/CRTC2/FLOT1/CLOCK/NFAM1/IRAK2/NEUROD1/TNFSF11*	35
GO:0051648	vesicle localization	27/880	306/18670	0.001364158	0.033366912	0.028392449	*GSK3B/PTEN/CLASP2/ERC2/MAP2/BLOC1S2/CDH2/CNR1/SAR1B/RAB11A/RAP1A/RAP1B/LMAN1/SEC23IP/SYT1/RIMS2/TBC1D20/APBA1/CPLX3/STARD3NL/PPP6R3/PRRT2/RIMS3/STX5/BACE1/KIF13A/PPP6C*	27
GO:0031345	negative regulation of cell projection organization	19/880	188/18670	0.001458848	0.034696391	0.029523724	*PRKCD/GSK3B/ULK2/PTEN/RNF6/MAP2/TBC1D30/UBE3A/EPHA7/SEMA6D/RYK/WNT5A/LINGO1/CARM1/SEMA3G/TCHP/TNR/DENND5A/NFATC4*	19
GO:0034063	stress granule assembly	5/880	19/18670	0.001538337	0.036419059	0.030989571	*G3BP2/DDX3X/EIF2S1/PRKAA2/DDX6*	5
GO:0030968	endoplasmic reticulum unfolded protein response	14/880	121/18670	0.001684543	0.038639679	0.032879132	*BFAR/CTH/EIF2S1/BAK1/CREBRF/EP300/CTDSP2/HSPA13/PPP1R15B/TMEM33/DERL2/CREB3L3/WFS1/KLHDC3*	14
GO:0048145	regulation of fibroblast proliferation	11/880	83/18670	0.001737408	0.039675946	0.033760909	*CDK6/PAWR/TP53INP1/CREB1/ESR1/CDC6/WNT5A/DACH1/FOSL2/PDGFA/ZMIZ1*	11
GO:1900376	regulation of secondary metabolite biosynthetic process	4/880	12/18670	0.001790369	0.04000041	0.034037	*SLC7A11/WNT5A/ZEB2/APPL1*	4
GO:0099173	postsynapse organization	17/880	163/18670	0.001809324	0.040249652	0.034249084	*SLC7A11/PTEN/PTPRD/DLG4/ADAM10/CDH2/HOMER1/UBE3A/EPHA7/WNT5A/GDNF/NRCAM/PAK3/DLG2/ARF4/COLQ/ABHD17B*	17
GO:0099003	vesicle-mediated transport in synapse	20/880	207/18670	0.001911119	0.041547405	0.035353363	*GSK3B/ERC2/MAP2/BLOC1S2/EPS15/PPP3CB/CNR1/RAB11A/RAP1A/ROCK1/RAP1B/NUMB/SYT1/RIMS2/APBA1/CPLX3/PRRT2/RIMS3/BACE1/SNCB*	20

GO:0048144	fibroblast proliferation	11/880	84/18670	0.001915962	0.041547405	0.035353363	*CDK6/PAWR/TP53INP1/CREB1/ESR1/CDC6/WNT5A/DACH1/FOSL2/PDGFA/ZMIZ1*	11
GO:1902803	regulation of synaptic vesicle transport	11/880	84/18670	0.001915962	0.041547405	0.035353363	*GSK3B/MAP2/CNR1/RAP1A/RAP1B/SYT1/RIMS2/APBA1/CPLX3/RIMS3/BACE1*	11
GO:1903846	positive regulation of cellular response to transforming growth factor beta stimulus	6/880	29/18670	0.002015994	0.042641585	0.036284419	*ADAM17/CREBBP/EP300/CDKN1C/DAB2/MEN1*	6
GO:0035303	regulation of dephosphorylation	20/880	209/18670	0.002141388	0.044296334	0.037692472	*MFHAS1/PRKCD/GSK3B/ARPP19/BOD1/BOD1L2/WNK1/PPP2R5A/PPP1R15B/ROCK1/PPP1R11/ZFYVE1/MGAT5/CSRNP2/IGBP1/PPP6R3/CALM1/DLG2/PPME1/SLC39A10*	20
GO:0033002	muscle cell proliferation	22/880	239/18670	0.00216289	0.044296334	0.037692472	*SMAD1/PTEN/HPGD/NAMPT/PTGS2/PIM1/SKP2/JARID2/KLF4/PAK1/AKIRIN1/CTNNBIP1/MSTN/TRAF6/IRAK1/ERBB4/BMPR1A/APLN/IL6R/TGM2/ZFPM2/TGFBR1*	22
GO:0031333	negative regulation of protein complex assembly	15/880	139/18670	0.002352507	0.047242373	0.040199305	*PRKCD/GSK3B/ULK1/DDX3X/MAP2/MTPN/EP300/DYRK1A/TWF1/CAPZA1/CTNNBIP1/TMOD2/PTGER4/PRRT2/IAPP*	15
GO:0007611	learning or memory	23/880	256/18670	0.002374723	0.047503669	0.040421646	*SLC7A11/PTEN/PLCB1/ATP1A2/ITGA5/GMFB/PTGS2/DLG4/PPP3CB/CREB1/GRIN2A/EP300/CNR1/VDAC1/TMOD2/BDNF/SLC6A1/TNR/ARF4/NFATC4/CAMK4/LHX8/HIF1A*	23
GO:1904837	beta-catenin-TCF complex assembly	6/880	30/18670	0.002420949	0.047871714	0.040734821	*PYGO1/CREBBP/LEF1/EP300/MEN1/PYGO2*	6
GO:0140131	positive regulation of lymphocyte chemotaxis	5/880	21/18670	0.002489327	0.048316698	0.041113465	*STK39/ADAM17/ADAM10/WNK1/WNT5A*	5
GO:0001967	suckling behavior	4/880	13/18670	0.002490262	0.048316698	0.041113465	*UBR3/PEX13/DERL2/DACH1*	4
GO:0034620	cellular response to unfolded	15/880	140/18670	0.002523109	0.048588681	0.0413449	*BFAR/CTH/EIF2S1/BAK1/HSPA8/CREBRF/EP300/CTDSP2/HSPA13/PPP1R15B/TMEM33/DERL2/CRE*	15

	protein						*B3L3/WFS1/KLHDC3*	
GO:2001023	regulation of response to drug	12/880	100/18670	0.002587084	0.049451639	0.042079205	*STK39/HGF/ATP1A2/SYT10/PAWR/RGS4/GABRA4/HMGA2/CNR1/SYT1/GDNF/KMT2A*	12
GO:0006486	protein glycosylation	23/880	258/18670	0.002621291	0.049737076	0.042322088	*TET2/TET3/ST6GAL2/NUS1/TET1/GALNT10/C1GALT1/EDEM3/FUT9/GALNT7/B4GALT1/ST8SIA4/B4GALT4/NAGPA/UBE2J1/LMAN1/LFNG/MGAT5/ST6GAL1/B3GNT9/GALNT1/B3GNT5/DPY19L2*	23
GO:0043413	macromolecule glycosylation	23/880	258/18670	0.002621291	0.049737076	0.042322088	*TET2/TET3/ST6GAL2/NUS1/TET1/GALNT10/C1GALT1/EDEM3/FUT9/GALNT7/B4GALT1/ST8SIA4/B4GALT4/NAGPA/UBE2J1/LMAN1/LFNG/MGAT5/ST6GAL1/B3GNT9/GALNT1/B3GNT5/DPY19L2*	23
GO:0051650	establishment of vesicle localization	25/880	290/18670	0.002753637	0.05148009	0.043805247	*GSK3B/CLASP2/ERC2/MAP2/BLOC1S2/CNR1/SAR1B/RAB11A/RAP1A/RAP1B/LMAN1/SEC23IP/SYT1/RIMS2/TBC1D20/APBA1/CPLX3/STARD3NL/PPP6R3/PRRT2/RIMS3/STX5/BACE1/KIF13A/PPP6C*	25
GO:0042752	regulation of circadian rhythm	13/880	114/18670	0.002763027	0.05148009	0.043805247	*GSK3B/THRAP3/EZH2/USP9X/CREB1/BHLHE40/UBE3A/ZFHX3/SIAH2/HNRNPD/PRKAA2/SPSB4/CLOCK*	13
GO:0043403	skeletal muscle tissue regeneration	7/880	41/18670	0.002788442	0.051766722	0.044049147	*EZH2/MTPN/AKIRIN1/MSTN/PTGFRN/DAG1/GPX1*	7
GO:2001234	negative regulation of apoptotic signaling pathway	21/880	230/18670	0.002968153	0.054129458	0.046059638	*STRADB/HGF/CTH/DDX3X/PTGS2/BID/EYA3/TAF9B/RB1/SH3RF1/GATA4/SIAH2/GDNF/BDNF/WFS1/TPT1/PEA15/GPX1/DNAJA1/TGFBR1/HIF1A*	21
GO:0060390	regulation of SMAD protein signal transduction	5/880	22/18670	0.003098029	0.055134227	0.046914612	*TOB1/CILP/DAB2/BMPR1A/TGFBR1*	5
GO:0009896	positive regulation of catabolic process	33/880	423/18670	0.003281218	0.057773645	0.049160536	*TNRC6B/PRKCD/RBM24/LARP1/GSK3B/TRIB2/CHFR/PTEN/TOB1/MTDH/TNRC6C/TNRC6A/ULK1/GPSM1/PFKFB3/SH3D19/TP53INP1/PFKFB2/ANKIB1/YTHDF3/PRR5L/SESN3/WNT5A/ROCK1/DAB2/HNRNPD/LRP2/PRKAA2/HK2/DISC1/STX5/TRIM67/HIF1A*	33
GO:0050807	regulation of synapse	20/880	218/18670	0.003488496	0.059814377	0.050897028	*SLC7A11/PTEN/PTPRD/ADAM10/CDH2/HOMER1/UBE3A/EPHA7/W*	20

	organization						*NT5A/GPC4/NRCAM/BDNF/DAG1/DISC1/LRTM2/PAK3/ARF4/COLQ/NFATC4/ABHD17B*	
GO:2000300	regulation of synaptic vesicle exocytosis	10/880	78/18670	0.003543817	0.060163283	0.051193918	*GSK3B/CNR1/RAP1A/RAP1B/SYT1/RIMS2/APBA1/CPLX3/RIMS3/BACE1*	10
GO:0099072	regulation of postsynaptic membrane neurotransmitter receptor levels	9/880	66/18670	0.003655066	0.061047886	0.05194664	*DLG4/EPS15/ADAM10/RAB11A/RAP1A/GPC4/NUMB/DAG1/DLG2*	9
GO:0010639	negative regulation of organelle organization	31/880	395/18670	0.003934021	0.064141597	0.054579129	*HMGA1/ATAD2B/PRKCD/HGF/CLASP2/MTM1/CAMSAP1/GMFB/TBC1D4/MAP2/TBC1D30/TET1/MTPN/PAX5/TNKS2/TAOK1/JARID2/DYRK1A/TWF1/CAPZA1/LMAN1/TMOD2/MTF2/TCHP/POT1/PRRT2/GPX1/TINF2/MAD2L1/IAPP/TMEFF2*	31
GO:0072089	stem cell proliferation	13/880	120/18670	0.004317722	0.068146068	0.057986599	*PIM1/LEF1/HMGA2/WNT5A/ZNRF3/NUMB/DISC1/SOX5/THPO/WNT2B/DAGLA/HIF1A/SOX11*	13
GO:0034976	response to endoplasmic reticulum stress	24/880	285/18670	0.004445449	0.069105304	0.058802828	*GSK3B/CHAC1/UBE4B/BFAR/CTH/EIF2S1/BAK1/PMAIP1/CREBRF/EP300/UBE2K/ERLIN1/EDEM3/USP25/CTDSP2/HSPA13/PPP1R15B/TMEM33/DERL2/UBE2J1/CREB3L3/WFS1/FLOT1/KLHDC3*	24
GO:0007623	circadian rhythm	19/880	208/18670	0.004551603	0.070106445	0.059654716	*GSK3B/THRAP3/PTEN/EZH2/NAMPT/USP9X/CREB1/BHLHE40/UBE3A/EP300/DYRK1A/ZFHX3/SIAH2/HNRNPD/PRKAA2/MYCBP2/KMT2A/SPSB4/CLOCK*	19
GO:0007548	sex differentiation	23/880	270/18670	0.004600258	0.070106445	0.059654716	*HOXA9/INHBB/BAK1/PATZ1/DMRT3/HOXD13/SALL1/ESR1/UBE3A/WNT5A/GATA4/LRP2/LFNG/TBC1D20/BMPR1A/DACH1/ACVR1B/MMP14/WNT2B/HOXA11/ZFPM2/TGFBR1/LHX8*	23
GO:0046835	carbohydrate phosphorylation	5/880	24/18670	0.004625148	0.070106445	0.059654716	*PFKFB3/PFKFB2/IPPK/HK2/KHK*	5
GO:0086064	cell communication by electrical coupling involved in	5/880	24/18670	0.004625148	0.070106445	0.059654716	*ATP1A2/PDE4D/GJC1/CALM1/ATP1B2*	5

	cardiac conduction							
GO:0110110	positive regulation of animal organ morphogenesis	10/880	81/18670	0.004663256	0.070106445	0.059654716	*TFAP2A/JAG1/PIM1/ROBO1/GDNF/FGF1/CSF1/WNT2B/HOXA11/TGFBR1*	10
GO:0048255	mRNA stabilization	7/880	45/18670	0.00478299	0.070903557	0.060332992	*RBM24/LARP1/THRAP3/RBM46/HNRNPD/GDNF/MAPKAPK2*	7
GO:0086003	cardiac muscle cell contraction	9/880	69/18670	0.00493872	0.072617481	0.061791398	*KCNJ2/ATP1A2/PDE4B/KCNE4/PDE4D/GATA4/SCN3B/GJC1/STC1*	9
GO:0007568	aging	26/880	321/18670	0.005256783	0.076423262	0.065029798	*HMGA1/PRKCD/PTEN/LOXL2/ADM/NAMPT/CDK6/PAWR/PTGS2/EIF2S1/BAK1/PAX5/SLC12A2/CREB1/HMGA2/CNR1/CDKN1C/PDE4D/NEK6/CARM1/IRAK1/BMPR1A/MBD1/DAG1/ZMIZ1/SMC6*	26
GO:0071108	protein K48-linked deubiquitination	6/880	35/18670	0.005419145	0.078153994	0.066502506	*OTUD4/USP9X/USP37/USP27X/USP25/DESI2*	6
GO:0050803	regulation of synapse structure or activity	20/880	227/18670	0.00547867	0.078542817	0.066833362	*SLC7A11/PTEN/PTPRD/ADAM10/CDH2/HOMER1/UBE3A/EPHA7/WNT5A/GPC4/NRCAM/BDNF/DAG1/DISC1/LRTM2/PAK3/ARF4/COLQ/NFATC4/ABHD17B*	20
GO:1903306	negative regulation of regulated secretory pathway	5/880	25/18670	0.005560752	0.07869553	0.066963308	*REST/BCR/RAP1A/RAP1B/ABR*	5
GO:1905809	negative regulation of synapse organization	5/880	25/18670	0.005560752	0.07869553	0.066963308	*PTEN/UBE3A/EPHA7/WNT5A/NFATC4*	5
GO:0032970	regulation of actin filament-based process	30/880	388/18670	0.005565563	0.07869553	0.066963308	*PRKCD/KCNJ2/CLASP2/ATP1A2/VANGL2/RHOQ/GMFB/ABL2/RHOU/RGS4/PDE4B/MTPN/TAOK1/EPHA5/ARPC3/CELSR1/PAK1/TWF1/CAPZA1/PDE4D/ROCK1/GATA4/TMOD2/PTGER4/RHOBTB1/PDGFA/PAK3/STC1/TGFBR1/TMEFF2*	30
GO:0019372	lipoxygenase pathway	4/880	16/18670	0.005662239	0.078980583	0.067205864	*HPGD/PTGS2/PON1/GPX1*	4
GO:0055119	relaxation of cardiac muscle	4/880	16/18670	0.005662239	0.078980583	0.067205864	*KCNJ2/ATP1A2/PDE4B/PDE4D*	4
GO:0032386	regulation of intracellular	32/880	423/18670	0.005897622	0.082039358	0.069808626	*PRKCD/GSK3B/PDCD10/NUS1/PTGS2/MAP2/RHOU/C2CD5/PRR5L/*	32

	transport						*CNR1/SAR1B/PAK1/RAB21/RAP1A/DAB2/DERL2/UBE2J1/RAP1B/LMAN1/SYT1/RIMS2/TBC1D20/XPO4/APBA1/CPLX3/WWC1/SCP2/RIMS3/NUP153/PTPN14/BACE1/RBM27*	
GO:0090630	activation of GTPase activity	11/880	97/18670	0.005913319	0.082039358	0.069808626	*EPHA2/TBC1D15/TBC1D4/TBC1D30/BCR/EIF5/WNT5A/RABGAP1/PLEKHG4/ABR/RABGAP1L*	11
GO:0043647	inositol phosphate metabolic process	9/880	71/18670	0.005971993	0.082631253	0.070312279	*PTEN/PLCB1/NUDT11/IPPK/MINPP1/IPMK/PLCL1/PLCD1/CALM1*	9
GO:0043154	negative regulation of cysteine-type endopeptidase activity involved in apoptotic process	10/880	84/18670	0.006043478	0.082953158	0.070586193	*HGF/DDX3X/PTGS2/PAK2/LEF1/SH3RF1/KLF4/SIAH2/IGBP1/GPX1*	10
GO:0045445	myoblast differentiation	10/880	84/18670	0.006043478	0.082953158	0.070586193	*RBM24/PLCB1/REST/JAG1/RB1/DDX17/AKIRIN1/SOSTDC1/MSTN/MBNL3*	10
GO:0061614	pri-miRNA transcription by RNA polymerase II	7/880	47/18670	0.006111945	0.083670426	0.071196528	*SMAD1/KLF4/BMPR1A/APLN/ETS1/NFATC4/HIF1A*	7
GO:0017156	calcium ion regulated exocytosis	15/880	154/18670	0.006191459	0.0843753	0.071796317	*GSK3B/ERC2/SYT10/REST/PPP3CB/CNR1/RAP1A/RAP1B/SYT1/RIMS2/APBA1/CPLX3/PRRT2/RIMS3/BACE1*	15
GO:0009743	response to carbohydrate	20/880	230/18670	0.006320074	0.085164239	0.072467638	*PTEN/ACVR1C/NAMPT/PTGS2/PPP3CB/PFKFB2/EPHA5/RAP1A/GATA4/RAP1B/KLF7/PRKAA2/MEN1/LPL/ENY2/SLC6A1/KHK/MAP2K6/NEUROD1/HIF1A*	20
GO:1904018	positive regulation of vasculature development	20/880	230/18670	0.006320074	0.085164239	0.072467638	*HGF/SMAD1/MTDH/ITGA5/ADM/CTH/PTGS2/ITGB8/HMGA2/KLF4/HIPK1/WNT5A/ADAM12/RAP1A/GATA4/HK2/FGF1/IL6R/ETS1/HIF1A*	20
GO:0010460	positive regulation of heart rate	5/880	26/18670	0.006622296	0.087187928	0.074189627	*ADM/RGS4/SLC1A1/PDE4D/SCN3B*	5
GO:0043090	amino acid import	5/880	26/18670	0.006622296	0.087187928	0.074189627	*SLC7A11/ATP1A2/RGS4/SLC1A1/SLC6A1*	5
GO:0080111	DNA demethylation	5/880	26/18670	0.006622296	0.087187928	0.074189627	*TET2/OTUD4/TET3/USP9X/TET1*	5

GO:0010107	potassium ion import	7/880	48/18670	0.00687124	0.089211084	0.075911164	*KCNJ2/ATP1A2/WNK3/SLC12A2/WNK1/KCNJ14/ATP1B2*	7
GO:0090342	regulation of cell aging	8/880	60/18670	0.006879677	0.089211084	0.075911164	*HMGA1/PTEN/NAMPT/CDK6/PAWR/HMGA2/NEK6/BMPR1A*	8
GO:0043087	regulation of GTPase activity	35/880	479/18670	0.006989054	0.089776259	0.076392081	*GSK3B/FGD1/EZH2/PLCB1/EPHA2/SRGAP1/TBC1D15/TBC1D4/DOCK4/DEPDC1/GPSM1/NUP50/RGS4/TBC1D30/BCR/WNK1/EPHA5/ARHGAP21/ACAP2/ARHGAP26/PLXNA2/EIF5/WNT5A/CPEB2/RAP1A/RP2/RABGAP1/TBC1D20/MAPRE2/PLEKHG4/ABR/RABGAP1L/TBC1D24/ALS2CL/PROM2*	35
GO:0050890	cognition	24/880	296/18670	0.007054366	0.089776259	0.076392081	*SLC7A11/PTEN/PLCB1/ATP1A2/ITGA5/GMFB/PTGS2/DLG4/PPP3CB/CREB1/GRIN2A/EP300/CNR1/VDAC1/TMOD2/BDNF/SLC6A1/TNR/ARF4/GPR155/NFATC4/CAMK4/LHX8/HIF1A*	24
GO:0015693	magnesium ion transport	4/880	17/18670	0.007132003	0.089776259	0.076392081	*KCNJ2/NIPA1/NIPAL2/ZDHHC13*	4
GO:0033599	regulation of mammary gland epithelial cell proliferation	4/880	17/18670	0.007132003	0.089776259	0.076392081	*HOXA5/ROBO1/PYGO2/GPX1*	4
GO:0035988	chondrocyte proliferation	4/880	17/18670	0.007132003	0.089776259	0.076392081	*MMP16/HMGA2/MMP14/STC1*	4
GO:1905331	negative regulation of morphogenesis of an epithelium	4/880	17/18670	0.007132003	0.089776259	0.076392081	*SULF1/PTEN/CLASP2/WNT5A*	4
GO:0000289	nuclear-transcribed mRNA poly(A) tail shortening	6/880	37/18670	0.007167457	0.089784572	0.076399154	*TNRC6B/TOB1/TNRC6C/TNRC6A/PAN3/SAMD4B*	6
GO:1902904	negative regulation of supramolecular fiber organization	14/880	142/18670	0.007222781	0.09025853	0.076802453	*PRKCD/CLASP2/CAMSAP1/GMFB/MAP2/HSPA8/MTPN/TAOK1/DYRK1A/TWF1/CAPZA1/TMOD2/IAPP/TMEFF2*	14
GO:0030100	regulation of endocytosis	23/880	281/18670	0.007382287	0.091807189	0.078120232	*PTEN/ABL2/BCR/DLG4/PPP3CB/MIB1/RAB21/WNT5A/RAB31/RAP1A/ROCK1/DAB2/APPL1/NUMB/AAK1/APLN/LRPAP1/ABR/FLOT1/MCTP1/SNX17/PROM2/ATG3*	23
GO:0001819	positive	34/880	464/18670	0.007456985	0.092291362	0.078532223	*HGF/SULF1/PLCB1/POLR3G/ATF*	34

	regulation of cytokine production						2/ADAM17/PRKCQ/CD200/DDX3X/KPNA6/PTGS2/PDE4B/CREB1/CREBBP/FAM49B/EP300/WNT5A/CEBPG/PDE4D/GATA4/TRAF6/IRAK1/CD80/APPL1/PTGER4/IL20RB/LPL/FLOT1/NFAM1/MAPKAPK2/IL6R/NFATC4/CLEC6A/HIF1A	
GO:0034113	heterotypic cell-cell adhesion	8/880	61/18670	0.007599601	0.093162807	0.07927375	*ITGA5/CD200/CXADR/WNK1/KLF4/NRCAM/FLOT1/GLDN*	8
GO:0030278	regulation of ossification	18/880	203/18670	0.007641827	0.093458458	0.079525324	*HGF/SMAD1/TOB1/CDK6/REST/TFAP2A/JAG1/CHSY1/WNT5A/TMEM64/CTNNBIP1/BMPR1A/PTGER4/MEN1/CSF1/IL6R/HIF1A/SOX11*	18
GO:0032409	regulation of transporter activity	23/880	283/18670	0.008012349	0.095648045	0.081388479	*STK39/PRKCD/PTEN/ATP1A2/CTNBP2NL/STAC2/WNK3/DLG4/PDE4B/PON1/KCNE4/CACNB4/WNK1/GRIN2A/HOMER1/PDE4D/SCN3B/KMT2A/KCNC1/CALM1/ATP1B2/DLG2/MINK1*	23
GO:0046660	female sex differentiation	12/880	115/18670	0.008018746	0.095648045	0.081388479	*INHBB/BAK1/ESR1/UBE3A/WNT5A/LRP2/LFNG/DACH1/ACVR1B/MMP14/ZFPM2/LHX8*	12
GO:0098727	maintenance of cell number	15/880	159/18670	0.008247566	0.097852155	0.083263992	*EZH2/LOXL2/VANGL2/REST/JAG1/TET1/RTF1/CDH2/SALL1/HMGA2/KLF4/BMPR1A/MTF2/DDX6/HOOK3*	15
GO:0010766	negative regulation of sodium ion transport	4/880	18/18670	0.0088329	0.102904286	0.087562933	*STK39/ATP1A2/WNK3/WNK1*	4
GO:1905276	regulation of epithelial tube formation	4/880	18/18670	0.0088329	0.102904286	0.087562933	*VANGL2/CELSR1/WNT5A/GDNF*	4
GO:0048340	paraxial mesoderm morphogenesis	3/880	10/18670	0.009762019	0.109983015	0.093586338	*LEF1/WNT5A/BMPR1A*	3
GO:0097050	type B pancreatic cell apoptotic process	3/880	10/18670	0.009762019	0.109983015	0.093586338	*EIF2S1/WFS1/NEUROD1*	3
GO:1903789	regulation of amino acid transmembrane transport	3/880	10/18670	0.009762019	0.109983015	0.093586338	*STK39/ATP1A2/RGS4*	3
GO:2000049	positive regulation of cell-cell	3/880	10/18670	0.009762019	0.109983015	0.093586338	*ADAM19/WNT5A/FLOT1*	3

	adhesion mediated by cadherin							
GO:0001505	regulation of neurotransmitter levels	27/880	354/18670	0.009781477	0.109983015	0.093586338	*SLC7A11/GSK3B/ATP1A2/ERC2/ALDH5A1/PTGS2/RGS4/HSPA8/GRIN2A/SLC1A1/CNR1/KLF4/RAP1A/RAP1B/SYT1/RIMS2/GDNF/APBA1/CPLX3/FLOT1/SLC6A1/MCTP1/PRRT2/DAGLA/RIMS3/COLQ/BACE1*	27
GO:0051668	localization within membrane	14/880	148/18670	0.01024976	0.114748398	0.09764128	*SLC7A11/ANKS1B/DLG4/EPS15/ADAM10/CDH2/RAB11A/RAP1A/GPC4/NUMB/TBC1D20/DAG1/DLG2/COLQ*	14
GO:0001952	regulation of cell-matrix adhesion	12/880	119/18670	0.010414027	0.115397647	0.098193738	*GSK3B/PTEN/CLASP2/CDK6/JAG1/ROCK1/MMP14/DAG1/DISC1/CSF1/MINK1/HOXA7*	12
GO:0099504	synaptic vesicle cycle	17/880	194/18670	0.010457988	0.115397647	0.098193738	*GSK3B/PTEN/ERC2/PPP3CB/CDH2/CNR1/RAP1A/ROCK1/RAP1B/SYT1/RIMS2/APBA1/CPLX3/PRRT2/RIMS3/BACE1/SNCB*	17
GO:0010644	cell communication by electrical coupling	5/880	29/18670	0.010643147	0.115397647	0.098193738	*ATP1A2/PDE4D/GJC1/CALM1/ATP1B2*	5
GO:0033032	regulation of myeloid cell apoptotic process	5/880	29/18670	0.010643147	0.115397647	0.098193738	*SLC7A11/PTEN/ADAM17/MAEA/PIK3CD*	5
GO:0044550	secondary metabolite biosynthetic process	5/880	29/18670	0.010643147	0.115397647	0.098193738	*SLC7A11/CTH/WNT5A/ZEB2/APPL1*	5
GO:0001710	mesodermal cell fate commitment	4/880	19/18670	0.010778117	0.116129152	0.098816187	*SMAD1/KLF4/BMPR1A/HOXA11*	4
GO:0036342	post-anal tail morphogenesis	4/880	19/18670	0.010778117	0.116129152	0.098816187	*EPHA2/VANGL2/WNT5A/DCHS1*	4
GO:0070989	oxidative demethylation	4/880	19/18670	0.010778117	0.116129152	0.098816187	*TET2/TET3/TET1/CYP3A7*	4

Table S6c. GO_CC enrichment analysis. CC: Cellular Component.

ID	Description	GeneRatio	BgRatio	pvalue	p.adjust	qvalue	geneID	Count
GO:0098978	glutamatergic synapse	38/917	349/19717	1.06018E-06	0.000637169	0.000571382	*GSK3B/ADAM23/PLCB1/PTPRD/ERC2/TANC2/BCR/DLG4/PAK2/EPS15/ADAM10/PPP3CB/GRIN2A/HOMER1/EPHA7/CNR1/RAB11A/WNT5A/PPM1H/PURA/RAP1A/GPC4/E*	38

							RBB4/NUMB/SYT1/GRID1/NRCAM/GRM1/APBA1/ABR/DAG1/FLOT1/PAK3/DLG2/TNR/ARF4/CAMK4/ABHD17B	
GO:0005667	transcription factor complex	37/917	365/19717	7.78398E-06	0.002339087	0.002097579	*HMGA1/E2F7/SMAD1/NAA15/RCOR1/HOXA9/MXI1/RFX3/EYA3/TAF9B/CREB1/GTF3C2/RB1/LEF1/TCF12/EP300/KLF4/GTF2A1/CARM1/GATA4/ZFHX3/RARB/TAF5/DACH1/MED8/PYGO2/PKNOX1/CLOCK/TAF11/ETS1/HOXA11/NR6A1/NFATC4/ATF7IP/NEUROD1/HIF1A/SOX11*	37
GO:0044798	nuclear transcription factor complex	22/917	201/19717	0.000176458	0.035350447	0.03170055	*HMGA1/E2F7/MXI1/TAF9B/CREB1/GTF3C2/RB1/LEF1/TCF12/KLF4/GTF2A1/CARM1/GATA4/RARB/TAF5/MED8/PYGO2/TAF11/NFATC4/NEUROD1/HIF1A/SOX11*	22
GO:0097060	synaptic membrane	37/917	432/19717	0.000273602	0.041108628	0.036864204	*PTEN/ADAM23/ERC2/ANKS1B/BCR/GABRA4/DLG4/ADAM10/PSD3/CDH2/GRIN2A/HOMER1/EPHA7/CNR1/GPC4/ERBB4/SYT1/RIMS2/ZNRF2/PTPRA/GRID1/NRCAM/STRN/GRM1/APBA1/CPLX3/DAG1/DISC1/KCNC1/SLC6A1/DLG2/PRRT2/DAGLA/RIMS3/MINK1/CPEB4/ABHD17B*	37
GO:1902911	protein kinase complex	14/917	109/19717	0.000527002	0.04938297	0.04428423	*ACVR1C/CCNJL/ULK1/CDK6/CCNJ/RB1/CDK13/CCND2/PRKAA2/ACVR1B/CDK9/SORBS1/CCNG1/TGFBR1*	14
GO:0000307	cyclin-dependent protein kinase holoenzyme complex	8/917	42/19717	0.000612294	0.04938297	0.04428423	*CCNJL/CDK6/CCNJ/RB1/CDK13/CCND2/CDK9/CCNG1*	8
GO:0035770	ribonucleoprotein granule	22/917	223/19717	0.000747962	0.04938297	0.04428423	*TNRC6B/LARP1/G3BP2/TNRC6C/TNRC6A/PAN3/MEX3B/DDX3X/EIF2S1/HELZ/LARP4/LARP4B/USP3/ROCK1/GRSF1/PRKAA2/CDK9/SAMD4B/DDX6/CLOCK/EIF4A2/CARHSP1*	22
GO:1902554	serine/threonine protein kinase complex	12/917	88/19717	0.000753848	0.04938297	0.04428423	*ACVR1C/CCNJL/ULK1/CDK6/CCNJ/RB1/CDK13/CCND2/ACVR1B/CDK9/CCNG1/TGFBR1*	12
GO:0010494	cytoplasmic stress granule	10/917	66/19717	0.000891771	0.04938297	0.04428423	*LARP1/G3BP2/DDX3X/EIF2S1/LARP4/LARP4B/ROCK1/PRKAA2/DDX6/EIF4A2*	10

GO:0036464	cytoplasmic ribonucleoprotein granule	21/917	212/19717	0.000926378	0.04938297	0.04428423	*TNRC6B/LARP1/G3BP2/TNRC6C/TNRC6A/PAN3/MEX3B/DDX3X/EIF2S1/HELZ/LARP4/LARP4B/USP3/ROCK1/PRKAA2/CDK9/SAMD4B/DDX6/CLOCK/EIF4A2/CARHSP1*	21
GO:0098984	neuron to neuron synapse	30/917	350/19717	0.000986016	0.04938297	0.04428423	*ANKS1B/MAP2/TANC2/BCR/DLG4/PAK2/AKAP7/PDE4B/ADAM10/PSD3/CDH2/GRIN2A/HOMER1/MIB1/EPHA7/ERBB4/SYT1/GRID1/NRCAM/STRN/GRM1/ABR/DISC1/PAK3/DLG2/PRRT2/MINK1/BACE1/CPEB4/ABHD17B*	30
GO:0032580	Golgi cisterna membrane	12/917	92/19717	0.00112431	0.051977733	0.046611085	*ST6GAL2/GOLGA3/SAR1B/CHSY1/RAB21/FUT9/B4GALT1/B4GALT4/NAGPA/SORT1/ST6GAL1/GALNT1*	12
GO:0000151	ubiquitin ligase complex	25/917	282/19717	0.001585297	0.068054535	0.061027974	*KLHL42/FBXO11/UBR3/UBE4B/ZSWIM6/FBXL19/KBTBD8/DCUN1D3/SKP2/DCAF7/MAEA/ANKIB1/RANBP9/UBR1/UBE2D1/RANBP10/DERL2/DCAF12/ARMC8/ANAPC7/RNF8/SPSB4/TSPAN17/UBR2/ATG3*	25
GO:0016605	PML body	12/917	99/19717	0.002131004	0.085382213	0.076566587	*CHFR/PTEN/RNF6/TP53INP1/MKNK2/RB1/HIPK1/HIPK3/SATB1/TOPBP1/CDK9/SMC6*	12
GO:0014069	postsynaptic density	27/917	324/19717	0.002567328	0.090762607	0.081391461	*ANKS1B/MAP2/TANC2/BCR/DLG4/PAK2/PDE4B/ADAM10/PSD3/CDH2/GRIN2A/HOMER1/MIB1/EPHA7/ERBB4/GRID1/NRCAM/STRN/GRM1/ABR/DISC1/PAK3/DLG2/PRRT2/MINK1/CPEB4/ABHD17B*	27
GO:0032279	asymmetric synapse	27/917	328/19717	0.003042988	0.097038153	0.087019063	*ANKS1B/MAP2/TANC2/BCR/DLG4/PAK2/PDE4B/ADAM10/PSD3/CDH2/GRIN2A/HOMER1/MIB1/EPHA7/ERBB4/GRID1/NRCAM/STRN/GRM1/ABR/DISC1/PAK3/DLG2/PRRT2/MINK1/CPEB4/ABHD17B*	27
GO:0000790	nuclear chromatin	30/917	377/19717	0.003067762	0.097038153	0.087019063	*EZH2/SRCAP/PLCB1/POLR3G/PAWR/RFX3/EP400/CREB1/CREBBP/ESR1/DFFB/RB1/NACC2/TCF12/CDK13/KLF4/ZEB2/USP3/SATB1/TAF5/MEN1/CDK9/MBD1/SMARCA2/TRPS1/KLHDC3/GATAD2B/EME1/HMGB3/HIF1A*	30

Table S6d. GO_MF enrichment analysis. MF: Molecular Function.

ID	Description	GeneRatio	BgRatio	pvalue	p.adjust	qvalue	geneID	Count
GO:0004674	protein	50/894	439/17697	6.51197E-08	5.73053E-05	5.29183E-05	*STK39/PRKCD/STRADB/GSK3B/C*	50

	serine/threonine kinase activity						*DK8/ULK2/ACVR1C/PRKCQ/RPS6KA6/ULK1/NLK/MAPK6/CDK6/MARK1/WNK3/MAP3K9/PIM1/BCR/PAK2/MAP3K2/CSNK1G1/WNK1/TAOK1/MKNK2/CDK13/DYRK1A/HIPK1/PAK1/SOSTDC1/NEK6/ROCK1/LTBP1/IRAK1/HIPK3/AAK1/BMPR1A/PRKAA2/ACVR1B/CDK9/KSR2/ALPK3/PAK3/MAP4K5/MAPKAPK2/MINK1/DCLK3/MAP2K6/IRAK2/CAMK4/TGFBR1*	
GO:0046332	SMAD binding	17/894	80/17697	3.92416E-07	0.000172663	0.000159445	*SMAD1/TOB1/ACVR1C/USP15/USP9X/HMGA2/TCF12/COL1A2/ZEB2/IPO7/PURA/GATA4/DAB2/BMPR1A/MEN1/ACVR1B/TGFBR1*	17
GO:0019787	ubiquitin-like protein transferase activity	42/894	407/17697	9.22622E-06	0.002338987	0.002159926	*KLHL42/CHFR/FBXO11/RNF6/UBR3/UBE4B/ZNF598/BFAR/UBE2H/UBE2G1/G2E3/RNF141/SKP2/HECTD4/MAEA/ANKIB1/PELI2/UBR1/UBE3A/MIB1/UBE2E2/UBE2K/SH3RF1/UBE2W/UBE2D1/HECTD3/ZNRF3/UBE2J1/TRAF6/SIAH2/FBXW2/PPP1R11/ZNRF2/NEURL4/MYCBP2/RNF24/RNF8/UBE2R2/ZMIZ1/TSPAN17/UBR2/ATG3*	42
GO:0004842	ubiquitin-protein transferase activity	40/894	382/17697	1.06318E-05	0.002338987	0.002159926	*KLHL42/CHFR/FBXO11/RNF6/UBR3/UBE4B/ZNF598/BFAR/UBE2H/UBE2G1/G2E3/RNF141/SKP2/HECTD4/MAEA/ANKIB1/PELI2/UBR1/UBE3A/MIB1/UBE2E2/UBE2K/SH3RF1/UBE2W/UBE2D1/HECTD3/ZNRF3/UBE2J1/TRAF6/SIAH2/FBXW2/PPP1R11/ZNRF2/NEURL4/MYCBP2/RNF24/RNF8/UBE2R2/TSPAN17/UBR2*	40
GO:0051721	protein phosphatase 2A binding	9/894	32/17697	2.01505E-05	0.003546484	0.003274983	*MFHAS1/ARPP19/CTTNBP2NL/BOD1/BOD1L2/STRN/IGBP1/PPME1/STRN3*	9
GO:0004675	transmembrane receptor protein serine/threonine kinase activity	6/894	17/17697	0.000125073	0.018344013	0.016939687	*ACVR1C/SOSTDC1/LTBP1/BMPR1A/ACVR1B/TGFBR1*	6
GO:0035497	cAMP response element binding	5/894	14/17697	0.000444299	0.050548978	0.046679199	*ATF2/CREB1/HMGA2/TCF12/CREB3L3*	5
GO:0001228	DNA-binding	39/894	439/17697	0.000459536	0.050548978	0.046679199	*SMAD1/ATF2/HOXA5/REST/TFAP*	39

	transcription activator activity, RNA polymerase II-specific						*2A/PATZ1/PAX5/HOXD13/CREB1/CREBRF/ESR1/LEF1/HMGA2/TCF12/EP300/KLF4/CEBPG/TFAP2E/ZEB2/GATA4/KLF7/MYBL1/MYT1/CREB3L3/FOSL2/CSRNP2/PKNOX1/HNF1A/CLOCK/ZNF24/MLXIP/TOX2/ETS1/NR6A1/NEUROD1/HOXA7/HIF1A/SOX11/ETV1*	
GO:0008013	beta-catenin binding	12/894	82/17697	0.00081355	0.052334779	0.048328289	*GSK3B/CXADR/CDH2/SALL1/ESR1/LEF1/EP300/KLF4/TBL1XR1/CARM1/CTNNBIP1/NUMB*	12
GO:0070016	armadillo repeat domain binding	4/894	10/17697	0.001063262	0.062378052	0.057602698	*LEF1/CTNNBIP1/STRN/STRN3*	4
GO:0003713	transcription coactivator activity	29/894	319/17697	0.001661682	0.090991209	0.084025375	*HMGA1/THRAP3/ZBTB18/MTDH/SRCAP/TFAP2A/RAP2C/CREBBP/RB1/UBE3A/EP300/DDX17/MYCBP/KMT2C/GTF2A1/NFE2L3/CARM1/GATA4/KLF7/RARB/WWC1/ENY2/SMARCA2/ZMIZ1/TAF11/NFATC4/ZFPM2/NEUROD1/SOX11*	29
GO:0032934	sterol binding	9/894	56/17697	0.001836051	0.090991209	0.084025375	*OSBPL11/GRAMD1C/ERLIN1/OSBPL2/STARD3NL/SCP2/PROM2/OSBPL8/STARD4*	9
GO:0008080	N-acetyltransferase activity	12/894	90/17697	0.001861184	0.090991209	0.084025375	*EPC1/SRCAP/ATF2/NAA15/TAF9B/CREBBP/GNPNAT1/EP300/TAF5/CLOCK/NAT8L/ESCO1*	12
GO:0060090	molecular adaptor activity	23/894	237/17697	0.002110199	0.097735533	0.090253387	*OTUD4/TOB1/COL19A1/BFAR/HSPA8/GRB10/HOMER1/IPPK/PAG1/SH3RF1/COL1A2/COL11A1/DAB2/WWC2/SORBS2/LAT/MEN1/TRAT1/SORBS1/WWC1/SPSB4/DISC1/TEX261*	23
GO:0042393	histone binding	20/894	197/17697	0.002369396	0.104253429	0.096272305	*ATAD2B/SRCAP/USP15/PWWP2A/PHF6/CHD1/PYGO1/LEF1/KMT2C/TBL1XR1/USP3/MLLT3/IPO7/CARM1/MTF2/KMT2A/RNF8/PYGO2/SMARCA2/CDYL2*	20

Table S7. GEO expression array datasets involved in this study. GEO: Gene Expression Omnibus.

Data set	Year	Platform	Participants		
			Control	HSIL	Cancer
GSE63514*	2015	GPL570 [HG-U133_Plus_2]	24	62	28
GSE9750	2008	GPL96 [HG-U133A]	24	-	33
GSE7803	2007	GPL96 [HG-U133A]	10	7	21
GSE52903	2015	GPL6244 [HuGene-1_0-st]	17	-	51
GSE27678	2013	GPL96 [HG-U133A_2]	12	21	-

HSIL, high grade squamous intraepithelial lesions. *, In dataset GSE63514, CINII and CINIII were put under HSIL

Table S8a. Predicted target genes covered by the selected GEO datasets. GEO: Gene Expression Omnibus.

Genes covered by GPL6244	Genes covered by GPL570	Genes covered by GPL96			Genes not covered by any platform
GSE52903	GSE63514	GSE9750	GSE7803	GSE27678	
SRCAP	*MAB21L1*	*TET2*	*TET2*	*TET2*	*DISC1*
POM121C	*USP15*	*KLHL42*	*KLHL42*	*KLHL42*	*UBE2G1*
HOXA9	*ZNF410*	*ZNF462*	*ZNF462*	*ZNF462*	*SRCAP*
UBE2G1	*FAM136A*	*KIAA2013*	*KIAA2013*	*KIAA2013*	*ZNF512B*
RBM20	*SYT10*	*E2F7*	*E2F7*	*E2F7*	*BCR*
BCR	*POM121C*	*ACBD5*	*ACBD5*	*ACBD5*	*IGSF1*
MTPN	*SLC2A14*	*RBM24*	*RBM24*	*RBM24*	*PTPRA*
GMNC	*HOXA9*	*STRADB*	*STRADB*	*STRADB*	*APBA1*
MYCBP	*HEPHL1*	*EPC1*	*EPC1*	*EPC1*	
RDH14	*HSPA8*	*PALM3*	*PALM3*	*PALM3*	
MATR3	*MYCBP*	*ST6GAL2*	*ST6GAL2*	*ST6GAL2*	
SLC35E2B	*RDH14*	*STYX*	*STYX*	*STYX*	
TMEM64	*CARM1*	*NAP1L5*	*NAP1L5*	*NAP1L5*	
TMEM178B	*C17orf112*	*DCDC2*	*DCDC2*	*DCDC2*	
IGSF1	*XKR9*	*ATP11C*	*ATP11C*	*ATP11C*	
ZNF512B	*TPRX1*	*MAB21L1*	*MAB21L1*	*MAB21L1*	
PTPRA	*DBNDD2*	*ZIC5*	*ZIC5*	*ZIC5*	
C17orf112	*TXNRD3*	*UBR3*	*UBR3*	*UBR3*	
APBA1	*TRIM74*	*REEP3*	*REEP3*	*REEP3*	
DBNDD2	*C7orf43*	*THAP2*	*THAP2*	*THAP2*	
DISC1	*TRIM67*	*NHS*	*NHS*	*NHS*	
TSTD2	*CLEC6A*	*CPSF2*	*CPSF2*	*CPSF2*	
ZNF581	*CYP3A7*	*ACVR1C*	*ACVR1C*	*ACVR1C*	
TOX2	*FAM151B*	*RSPRY1*	*RSPRY1*	*RSPRY1*	
TXNRD3	*FAM19A1*	*USP15*	*USP15*	*USP15*	
IPO11	*COL4A3BP*	*ZSWIM6*	*ZSWIM6*	*ZSWIM6*	
C3orf62	*ECT2L*	*SRGAP1*	*SRGAP1*	*SRGAP1*	
TRIM74	*ANKRD20A3*	*FLVCR1*	*FLVCR1*	*FLVCR1*	
C7orf43	*ANKRD20A4*	*FBXL19*	*FBXL19*	*FBXL19*	
CYP3A7		*UBN2*	*UBN2*	*UBN2*	
FAM19A1		*VANGL2*	*VANGL2*	*VANGL2*	
COL4A3BP		*ZNF410*	*ZNF410*	*ZNF410*	
ECT2L		*ZNF598*	*ZNF598*	*ZNF598*	
ANKRD20A3		*LIN28B*	*LIN28B*	*LIN28B*	
ANKRD20A4		*MIER3*	*MIER3*	*MIER3*	
		FAM136A	*FAM136A*	*FAM136A*	
		CTTNBP2NL	*CTTNBP2NL*	*CTTNBP2NL*	
		TNRC6C	*TNRC6C*	*TNRC6C*	
		RBM46	*RBM46*	*RBM46*	
		KBTBD8	*KBTBD8*	*KBTBD8*	
		DNAJC21	*DNAJC21*	*DNAJC21*	
		SYT10	*SYT10*	*SYT10*	
		SAMD8	*SAMD8*	*SAMD8*	
		BOD1	*BOD1*	*BOD1*	

		POM121C	*POM121C*	*POM121C*	
		TNRC6A	*TNRC6A*	*TNRC6A*	
		PAN3	*PAN3*	*PAN3*	
		DCUN1D3	*DCUN1D3*	*DCUN1D3*	
		NUS1	*NUS1*	*NUS1*	
		MEX3B	*MEX3B*	*MEX3B*	
		PHF6	*PHF6*	*PHF6*	
		SLC2A14	*SLC2A14*	*SLC2A14*	
		HOXA9	*HOXA9*	*HOXA9*	
		STAC2	*STAC2*	*STAC2*	
		SAMD12	*SAMD12*	*SAMD12*	
		GPSM1	*GPSM1*	*GPSM1*	
		WNK3	*WNK3*	*WNK3*	
		NTN4	*NTN4*	*NTN4*	
		HAS3	*HAS3*	*HAS3*	
		LNX2	*LNX2*	*LNX2*	
		RHOU	*RHOU*	*RHOU*	
		BOD1L2	*BOD1L2*	*BOD1L2*	
		RBM20	*RBM20*	*RBM20*	
		TET1	*TET1*	*TET1*	
		SH3D19	*SH3D19*	*SH3D19*	
		PLEKHH1	*PLEKHH1*	*PLEKHH1*	
		DMRT3	*DMRT3*	*DMRT3*	
		G2E3	*G2E3*	*G2E3*	
		HEPHL1	*HEPHL1*	*HEPHL1*	
		ANKRD52	*ANKRD52*	*ANKRD52*	
		BLOC1S2	*BLOC1S2*	*BLOC1S2*	
		SLC30A7	*SLC30A7*	*SLC30A7*	
		HSPA8	*HSPA8*	*HSPA8*	
		ADAMTS19	*ADAMTS19*	*ADAMTS19*	
		SLC24A4	*SLC24A4*	*SLC24A4*	
		STRBP	*STRBP*	*STRBP*	
		FAM98B	*FAM98B*	*FAM98B*	
		RAB3IP	*RAB3IP*	*RAB3IP*	
		NIPA1	*NIPA1*	*NIPA1*	
		TP53INP1	*TP53INP1*	*TP53INP1*	
		TMEM68	*TMEM68*	*TMEM68*	
		INTS2	*INTS2*	*INTS2*	
		MTPN	*MTPN*	*MTPN*	
		TMEM86A	*TMEM86A*	*TMEM86A*	
		SLC2A13	*SLC2A13*	*SLC2A13*	
		ATPAF1	*ATPAF1*	*ATPAF1*	
		HOXC9	*HOXC9*	*HOXC9*	
		ZNF275	*ZNF275*	*ZNF275*	
		SLC45A4	*SLC45A4*	*SLC45A4*	
		FAXC	*FAXC*	*FAXC*	
		PLEKHG1	*PLEKHG1*	*PLEKHG1*	

		ARL5B	*ARL5B*	*ARL5B*	
		CREBRF	*CREBRF*	*CREBRF*	
		PHAX	*PHAX*	*PHAX*	
		OAF	*OAF*	*OAF*	
		GNPNAT1	*GNPNAT1*	*GNPNAT1*	
		NCEH1	*NCEH1*	*NCEH1*	
		ANKIB1	*ANKIB1*	*ANKIB1*	
		ZNF469	*ZNF469*	*ZNF469*	
		UBR1	*UBR1*	*UBR1*	
		ARHGAP21	*ARHGAP21*	*ARHGAP21*	
		PAG1	*PAG1*	*PAG1*	
		USP37	*USP37*	*USP37*	
		PCDH18	*PCDH18*	*PCDH18*	
		MIB1	*MIB1*	*MIB1*	
		LSM11	*LSM11*	*LSM11*	
		GMNC	*GMNC*	*GMNC*	
		FAM199X	*FAM199X*	*FAM199X*	
		OTUD1	*OTUD1*	*OTUD1*	
		UBE2E2	*UBE2E2*	*UBE2E2*	
		MYCBP	*MYCBP*	*MYCBP*	
		SCML4	*SCML4*	*SCML4*	
		SESN3	*SESN3*	*SESN3*	
		EAF1	*EAF1*	*EAF1*	
		ZNF385B	*ZNF385B*	*ZNF385B*	
		SH3RF1	*SH3RF1*	*SH3RF1*	
		TM4SF18	*TM4SF18*	*TM4SF18*	
		RDH14	*RDH14*	*RDH14*	
		COL22A1	*COL22A1*	*COL22A1*	
		KMT2C	*KMT2C*	*KMT2C*	
		IPMK	*IPMK*	*IPMK*	
		ACER3	*ACER3*	*ACER3*	
		CPEB2	*CPEB2*	*CPEB2*	
		FAM160A1	*FAM160A1*	*FAM160A1*	
		SLC22A23	*SLC22A23*	*SLC22A23*	
		KCNH7	*KCNH7*	*KCNH7*	
		LMLN	*LMLN*	*LMLN*	
		MATR3	*MATR3*	*MATR3*	
		TFAP2E	*TFAP2E*	*TFAP2E*	
		SLC35E2B	*SLC35E2B*	*SLC35E2B*	
		LINGO1	*LINGO1*	*LINGO1*	
		PDE7A	*PDE7A*	*PDE7A*	
		CCDC50	*CCDC50*	*CCDC50*	
		NEK6	*NEK6*	*NEK6*	
		PPP1R15B	*PPP1R15B*	*PPP1R15B*	
		ZNRF3	*ZNRF3*	*ZNRF3*	
		CARM1	*CARM1*	*CARM1*	
		UBTD2	*UBTD2*	*UBTD2*	

		FAM118B	*FAM118B*	*FAM118B*	
		TMEM64	*TMEM64*	*TMEM64*	
		KCTD18	*KCTD18*	*KCTD18*	
		MYPOP	*MYPOP*	*MYPOP*	
		TMEM178B	*TMEM178B*	*TMEM178B*	
		ARL6IP6	*ARL6IP6*	*ARL6IP6*	
		DCAF12	*DCAF12*	*DCAF12*	
		LCOR	*LCOR*	*LCOR*	
		ZBTB2	*ZBTB2*	*ZBTB2*	
		ZNF367	*ZNF367*	*ZNF367*	
		WWC2	*WWC2*	*WWC2*	
		NUMB	*NUMB*	*NUMB*	
		DDHD1	*DDHD1*	*DDHD1*	
		XKR4	*XKR4*	*XKR4*	
		VASN	*VASN*	*VASN*	
		TBC1D20	*TBC1D20*	*TBC1D20*	
		PTGFRN	*PTGFRN*	*PTGFRN*	
		ESYT2	*ESYT2*	*ESYT2*	
		ZFYVE1	*ZFYVE1*	*ZFYVE1*	
		SRSF12	*SRSF12*	*SRSF12*	
		ZNRF2	*ZNRF2*	*ZNRF2*	
		GRID1	*GRID1*	*GRID1*	
		ZBTB34	*ZBTB34*	*ZBTB34*	
		NEURL4	*NEURL4*	*NEURL4*	
		CREB3L3	*CREB3L3*	*CREB3L3*	
		PCDH19	*PCDH19*	*PCDH19*	
		SNRNP200	*SNRNP200*	*SNRNP200*	
		C17orf112	*C17orf112*	*C17orf112*	
		CYB561D1	*CYB561D1*	*CYB561D1*	
		ANAPC7	*ANAPC7*	*ANAPC7*	
		KIAA1549	*KIAA1549*	*KIAA1549*	
		APLN	*APLN*	*APLN*	
		ADAM33	*ADAM33*	*ADAM33*	
		SERTAD1	*SERTAD1*	*SERTAD1*	
		PLEKHG4	*PLEKHG4*	*PLEKHG4*	
		XKR9	*XKR9*	*XKR9*	
		IL20RB	*IL20RB*	*IL20RB*	
		PRTG	*PRTG*	*PRTG*	
		TPRX1	*TPRX1*	*TPRX1*	
		CRTC2	*CRTC2*	*CRTC2*	
		TCHP	*TCHP*	*TCHP*	
		DBNDD2	*DBNDD2*	*DBNDD2*	
		CYB5RL	*CYB5RL*	*CYB5RL*	
		SPSB4	*SPSB4*	*SPSB4*	
		PYGO2	*PYGO2*	*PYGO2*	
		GCSAM	*GCSAM*	*GCSAM*	
		FAM71E2	*FAM71E2*	*FAM71E2*	

		FRMD5	*FRMD5*	*FRMD5*	
		TSTD2	*TSTD2*	*TSTD2*	
		STARD3NL	*STARD3NL*	*STARD3NL*	
		UBE2R2	*UBE2R2*	*UBE2R2*	
		CACHD1	*CACHD1*	*CACHD1*	
		KSR2	*KSR2*	*KSR2*	
		ABLIM2	*ABLIM2*	*ABLIM2*	
		SLC43A2	*SLC43A2*	*SLC43A2*	
		TSPAN17	*TSPAN17*	*TSPAN17*	
		FMNL3	*FMNL3*	*FMNL3*	
		LRTM2	*LRTM2*	*LRTM2*	
		GLDN	*GLDN*	*GLDN*	
		FNDC5	*FNDC5*	*FNDC5*	
		NFAM1	*NFAM1*	*NFAM1*	
		FAM178B	*FAM178B*	*FAM178B*	
		ZNF25	*ZNF25*	*ZNF25*	
		LYSMD1	*LYSMD1*	*LYSMD1*	
		TBC1D24	*TBC1D24*	*TBC1D24*	
		ZNF581	*ZNF581*	*ZNF581*	
		GPR155	*GPR155*	*GPR155*	
		ABCB10	*ABCB10*	*ABCB10*	
		PRRT2	*PRRT2*	*PRRT2*	
		TMEM184A	*TMEM184A*	*TMEM184A*	
		TOX2	*TOX2*	*TOX2*	
		TXNRD3	*TXNRD3*	*TXNRD3*	
		GRPEL2	*GRPEL2*	*GRPEL2*	
		IPO11	*IPO11*	*IPO11*	
		DNAJC5G	*DNAJC5G*	*DNAJC5G*	
		B3GNT9	*B3GNT9*	*B3GNT9*	
		HYDIN	*HYDIN*	*HYDIN*	
		SAMD5	*SAMD5*	*SAMD5*	
		C9orf62	*C9orf62*	*C9orf62*	
		C1QTNF7	*C1QTNF7*	*C1QTNF7*	
		C3orf62	*C3orf62*	*C3orf62*	
		CDYL2	*CDYL2*	*CDYL2*	
		SP6	*SP6*	*SP6*	
		PROM2	*PROM2*	*PROM2*	
		NAT8L	*NAT8L*	*NAT8L*	
		EIF4A2	*EIF4A2*	*EIF4A2*	
		TRIM74	*TRIM74*	*TRIM74*	
		C7orf43	*C7orf43*	*C7orf43*	
		TRIM67	*TRIM67*	*TRIM67*	
		SUSD2	*SUSD2*	*SUSD2*	
		DCLK3	*DCLK3*	*DCLK3*	
		CLEC6A	*CLEC6A*	*CLEC6A*	
		GATAD2B	*GATAD2B*	*GATAD2B*	
		EME1	*EME1*	*EME1*	

		EGFLAM	*EGFLAM*	*EGFLAM*	
		IRAK2	*IRAK2*	*IRAK2*	
		ZSCAN29	*ZSCAN29*	*ZSCAN29*	
		EXOC8	*EXOC8*	*EXOC8*	
		CPEB4	*CPEB4*	*CPEB4*	
		RBM27	*RBM27*	*RBM27*	
		ZC3H6	*ZC3H6*	*ZC3H6*	
		CYP3A7	*CYP3A7*	*CYP3A7*	
		ZC3H12B	*ZC3H12B*	*ZC3H12B*	
		LHX8	*LHX8*	*LHX8*	
		CNTN4	*CNTN4*	*CNTN4*	
		FLG2	*FLG2*	*FLG2*	
		FAM151B	*FAM151B*	*FAM151B*	
		FAM19A1	*FAM19A1*	*FAM19A1*	
		COL4A3BP	*COL4A3BP*	*COL4A3BP*	
		ECT2L	*ECT2L*	*ECT2L*	
		SLC39A10	*SLC39A10*	*SLC39A10*	
		HOOK3	*HOOK3*	*HOOK3*	
		ASIC5	*ASIC5*	*ASIC5*	
		B3GNT5	*B3GNT5*	*B3GNT5*	
		STARD4	*STARD4*	*STARD4*	
		ANKRD20A3	*ANKRD20A3*	*ANKRD20A3*	
		ESCO1	*ESCO1*	*ESCO1*	
		TMEFF2	*TMEFF2*	*TMEFF2*	
		FMNL2	*FMNL2*	*FMNL2*	
		ANKRD20A4	*ANKRD20A4*	*ANKRD20A4*	
		DPY19L2	*DPY19L2*	*DPY19L2*	
		FREM2	*FREM2*	*FREM2*	
		SMIM15	*SMIM15*	*SMIM15*	

Explaination for compasion groups: CvsN: Cancer vs Normal; HvsN: HSIL vs Normal; HCvsN: HSIL and Cancer vs Normal (HSIL and cancer were grouped together). We extracted the union of down-regulated genes from five datasets as the most likely target genes for the six microRNAs (If one down-regulated gene changed inversely in different dataset, for example, it was up-regulated in one dataset, then the gene was excluded.

Table S8b. Expression profiles of predicted target genes of hsa-miR-26b-5p in GEO datasets.

Target genes	GSE63514(CvsN)	GSE63514(HvsN)	GSE63514(HCvsN)	GSE9750(CvsN)	GSE7803(CvsN)	GSE7803(HvsN)	GSE7803(HCvsN)	GSE52903(CvsN)	GSE27678(CvsN)
HMGA1	NOT	NOT	NOT	NOT	NOT	NOT	NOT	NOT	NOT
TET2	NOT	NOT	NOT	NA	NA	NA	NA	NOT	NA
FAM98A	NOT	NOT	NOT	NOT	NOT	NOT	NOT	NOT	NOT
ZDHHC6	NOT	NOT	NOT	NOT	NOT	NOT	NOT	NOT	NOT
TNRC6B	NOT	NOT	NOT	NOT	NOT	NOT	NOT	NOT	NOT
NABP1	NOT	NOT	NOT	NOT	NOT	NOT	NOT	NOT	NOT
KLHL42	NOT	NOT	NOT	NA	NA	NA	NA	NOT	NA
CASZ1	NOT	NOT	NOT	NOT	NOT	NOT	NOT	NOT	NOT
STK39	NOT	NOT	NOT	NOT	NOT	NOT	NOT	NOT	NOT
ZNF462	DOWN	NOT	NOT	NA	NA	NA	NA	NOT	NA
SLC7A11	NOT	NOT	NOT	NOT	NOT	NOT	NOT	NOT	NOT

KIAA2013	NOT	NOT	NOT	NA	NA	NA	NA	NOT	NA
MFHAS1	NOT	NOT	NOT	UP	UP	NOT	UP	NOT	UP
ATAD2B	NOT	NOT	NOT	NOT	NOT	NOT	NOT	NOT	NOT
CEP350	UP	NOT	NOT	NOT	NOT	NOT	NOT	NOT	NOT
SENP5	UP	NOT	UP	NOT	NOT	NOT	NOT	NOT	NOT
E2F7	UP	UP	UP	NA	NA	NA	NA	UP	NA
OTUD4	NOT	NOT	NOT	NOT	NOT	NOT	NOT	NOT	NOT
PRKCD	NOT	NOT	NOT	NOT	NOT	NOT	NOT	NOT	NOT
FRMD4B	DOWN	NOT	NOT	NOT	DOWN	NOT	DOWN	NOT	NOT
ACBD5	NOT	NOT	NOT	NA	NA	NA	NA	NOT	NA
RBM24	NOT	NOT	NOT	NA	NA	NA	NA	DOWN	NA
STRADB	NOT	NOT	NOT	NA	NA	NA	NA	NOT	NA
HGF	NOT	NOT	NOT	NOT	NOT	NOT	NOT	NOT	NOT
TET3	NOT	NOT	NOT	NOT	NOT	NOT	NOT	NOT	NOT
LARP1	NOT	NOT	NOT	NOT	NOT	NOT	NOT	NOT	NOT
GSK3B	NOT	NOT	NOT	NOT	NOT	NOT	NOT	NOT	NOT
EPC1	UP	NOT	NOT	NA	NA	NA	NA	NOT	NA
CDK8	NOT	NOT	NOT	NOT	NOT	NOT	NOT	NOT	NOT
TRIB2	NOT	NOT	NOT	NOT	NOT	NOT	NOT	NOT	NOT
SMAD1	NOT	NOT	NOT	NOT	NOT	NOT	NOT	NOT	NOT
PALM3	NOT	NOT	NOT	NA	NA	NA	NA	NOT	NA
ST6GAL2	DOWN	DOWN	DOWN	NA	NA	NA	NA	NOT	NA
THRAP3	NOT	NOT	NOT	NOT	NOT	NOT	NOT	NOT	NOT
CHFR	NOT	NOT	NOT	NOT	NOT	NOT	NOT	NOT	NOT
STYX	NOT	NOT	NOT	NA	NA	NA	NA	NOT	NA
SNN	NOT	NOT	NOT	NOT	NOT	NOT	NOT	NOT	NOT
SRP19	NOT	NOT	NOT	NOT	NOT	NOT	NOT	NOT	NOT
FGD1	NOT	NOT	NOT	NOT	NOT	NOT	NOT	NOT	NOT
KCNJ2	NOT	NOT	NOT	NOT	NOT	NOT	NOT	NOT	NOT
SULF1	UP	NOT	NOT	UP	NOT	NOT	NOT	NOT	NOT
ULK2	NOT	NOT	NOT	NOT	NOT	NOT	NOT	NOT	NOT
PTEN	NOT	NOT	NOT	NOT	NOT	NOT	NOT	NOT	NOT
EZH2	UP	UP	UP	UP	UP	NOT	UP	UP	UP
NAP1L5	NOT	NOT	NOT	NA	NA	NA	NA	NOT	NA
FBXO11	NOT	NOT	NOT	NOT	NOT	NOT	NOT	NOT	NOT
CHAC1	NOT	NOT	NOT	NOT	NOT	NOT	NOT	NOT	NOT
DCDC2	NOT	NOT	NOT	NA	NA	NA	NA	NOT	NA
SLC25A16	NOT	NOT	NOT	NOT	NOT	NOT	NOT	NOT	NOT
ATP11C	UP	NOT	UP	NA	NA	NA	NA	NOT	NA
MAB21L1	NA	NA	NA	NA	NA	NA	NA	NOT	NA
NAB1	NOT	NOT	NOT	NOT	NOT	NOT	NOT	NOT	NOT
TOB1	NOT	NOT	NOT	DOWN	DOWN	DOWN	DOWN	NOT	DOWN
ZIC5	NOT	NOT	NOT	NA	NA	NA	NA	NOT	NA
ZBTB18	NOT	NOT	NOT	NOT	NOT	NOT	NOT	NOT	NOT
RNF6	NOT	NOT	NOT	NOT	NOT	NOT	NOT	NOT	NOT
UBR3	NOT	NOT	NOT	NA	NA	NA	NA	NOT	NA
MTDH	NOT	NOT	NOT	NOT	NOT	NOT	NOT	NOT	NOT

BAZ2B	NOT	NOT	NOT	NOT	NOT	NOT	NOT	NOT	NOT
REEP3	NOT	NOT	NOT	NA	NA	NA	NA	NOT	NA
SRCAP	NOT	NOT	NOT	NOT	NOT	NOT	NOT	NA	NOT
CLASP2	NOT	NOT	NOT	NOT	NOT	NOT	NOT	NOT	NOT
THAP2	NOT	NOT	NOT	NA	NA	NA	NA	NOT	NA
UBE4B	NOT	NOT	NOT	NOT	NOT	NOT	NOT	NOT	NOT
ADAM23	NOT	NOT	NOT	NOT	NOT	NOT	NOT	NOT	NOT
ACSL3	NOT	NOT	NOT	NOT	NOT	NOT	NOT	NOT	NOT
ADAM19	UP	NOT	NOT	NOT	NOT	NOT	NOT	NOT	NOT
PLOD2	UP	UP	UP	UP	UP	NOT	UP	UP	NOT
CCDC6	NOT	NOT	NOT	NOT	NOT	NOT	NOT	NOT	NOT
FA2H	NOT	UP	UP	NOT	NOT	NOT	NOT	NOT	UP
NHS	NOT	NOT	NOT	NA	NA	NA	NA	NOT	NA
PLCB1	UP	NOT	UP	NOT	NOT	NOT	NOT	NOT	NOT
MTM1	NOT	NOT	NOT	NOT	NOT	NOT	NOT	NOT	NOT
ATP1A2	NOT	NOT	NOT	NOT	NOT	NOT	NOT	NOT	NOT
CPSF2	NOT	NOT	NOT	NA	NA	NA	NA	NOT	NA
ACVR1C	UP	NOT	NOT	NA	NA	NA	NA	NOT	NA
MRAS	UP	UP	UP	NOT	NOT	UP	NOT	NOT	NOT
CHORDC1	NOT	NOT	NOT	NOT	NOT	NOT	NOT	NOT	NOT
RSPRY1	NOT	NOT	NOT	NA	NA	NA	NA	NOT	NA
CILP	NOT	NOT	NOT	NOT	NOT	NOT	NOT	DOWN	UP
ANKS1A	NOT	NOT	NOT	NOT	NOT	NOT	NOT	NOT	NOT
POLR3G	NOT	NOT	NOT	NOT	NOT	NOT	NOT	NOT	DOWN
NUDT11	NOT	NOT	NOT	NOT	NOT	NOT	NOT	NOT	NOT
EPHA2	DOWN	NOT	NOT	NOT	NOT	DOWN	NOT	NOT	DOWN
COL19A1	NOT	NOT	NOT	NOT	NOT	NOT	NOT	NOT	NOT
PITPNC1	NOT	NOT	NOT	NOT	NOT	NOT	NOT	NOT	NOT
USP15	NA	NA	NA	NA	NA	NA	NA	NOT	NA
ZSWIM6	NOT	NOT	NOT	NA	NA	NA	NA	NOT	NA
ITGA5	NOT	NOT	NOT	NOT	NOT	NOT	NOT	NOT	NOT
PDCD10	UP	NOT	NOT	NOT	NOT	NOT	NOT	NOT	NOT
LOXL2	UP	NOT	NOT	UP	NOT	NOT	NOT	NOT	NOT
ARPP19	NOT	NOT	NOT	NOT	NOT	NOT	NOT	NOT	NOT
RPGR	NOT	NOT	NOT	NOT	NOT	NOT	NOT	NOT	NOT
SRGAP1	UP	NOT	NOT	NA	NA	NA	NA	NOT	NA
FRAT2	NOT	NOT	NOT	NOT	NOT	NOT	NOT	NOT	UP
FLVCR1	UP	UP	UP	NA	NA	NA	NA	NOT	NA
CCNJL	NOT	NOT	NOT	NOT	NOT	NOT	NOT	NOT	NOT
FBXL19	NOT	NOT	NOT	NA	NA	NA	NA	NOT	NA
ATF2	NOT	NOT	NOT	NOT	NOT	NOT	NOT	NOT	NOT
PTPRD	UP	NOT	NOT	NOT	NOT	NOT	NOT	DOWN	NOT
UBN2	NOT	NOT	NOT	NA	NA	NA	NA	NOT	NA
VANGL2	NOT	NOT	NOT	NA	NA	NA	NA	NOT	NA
ZNF410	NA	NA	NA	NA	NA	NA	NA	NOT	NA
G3BP2	NOT	NOT	NOT	NOT	NOT	NOT	NOT	NOT	NOT

ZNF598	NOT	NOT	NOT	NA	NA	NA	NA	NOT	NA
MSMO1	NOT	NOT	NOT	DOWN	DOWN	NOT	DOWN	NOT	DOWN
RHOQ	NOT	NOT	NOT	NOT	NOT	NOT	NOT	NOT	NOT
OSBPL11	UP	UP	UP	NOT	NOT	NOT	NOT	NOT	NOT
ADAM17	NOT	NOT	NOT	NOT	NOT	NOT	NOT	NOT	NOT
CCDC28A	NOT	NOT	NOT	NOT	NOT	NOT	NOT	NOT	NOT
LIN28B	NOT	NOT	NOT	NA	NA	NA	NA	NOT	NA
HOXA5	NOT	NOT	NOT	NOT	NOT	NOT	NOT	NOT	NOT
ADM	UP	NOT	NOT	NOT	NOT	DOWN	NOT	NOT	DOWN
MIER3	NOT	NOT	NOT	NA	NA	NA	NA	NOT	NA
SSX2IP	NOT	UP	UP	NOT	NOT	NOT	NOT	NOT	NOT
ERC2	NOT	NOT	NOT	NOT	NOT	NOT	NOT	NOT	NOT
LRRC2	NOT	NOT	NOT	NOT	NOT	NOT	NOT	NOT	NOT
FAM136A	NA	NA	NA	NA	NA	NA	NA	NOT	NA
PRKCQ	NOT	NOT	NOT	NOT	NOT	NOT	NOT	NOT	NOT
CTTNBP2NL	NOT	NOT	NOT	NA	NA	NA	NA	NOT	NA
RPS6KA6	DOWN	NOT	NOT	NOT	NOT	NOT	NOT	DOWN	NOT
CD200	NOT	NOT	NOT	NOT	NOT	NOT	NOT	NOT	NOT
LSM12	NOT	NOT	NOT	NOT	NOT	NOT	NOT	NOT	NOT
CAMSAP1	NOT	NOT	NOT	NOT	NOT	NOT	NOT	NOT	NOT
TTC13	UP	NOT	NOT	NOT	NOT	NOT	NOT	NOT	NOT
GPR52	NOT	NOT	NOT	NOT	NOT	NOT	NOT	NOT	NOT
TNRC6C	NOT	NOT	NOT	NA	NA	NA	NA	NOT	NA
RBM46	NOT	NOT	NOT	NA	NA	NA	NA	NOT	NA
ANKS1B	NOT	NOT	NOT	NOT	NOT	NOT	NOT	NOT	NOT
KBTBD8	NOT	NOT	NOT	NA	NA	NA	NA	NOT	NA
GPALPP1	NOT	NOT	NOT	NOT	NOT	NOT	NOT	NOT	NOT
DNAJC21	NOT	NOT	NOT	NA	NA	NA	NA	NOT	NA
SYT10	NA	NA	NA	NA	NA	NA	NA	NOT	NA
TBC1D15	UP	UP	UP	NOT	NOT	NOT	NOT	NOT	NOT
SAMD8	NOT	NOT	NOT	NA	NA	NA	NA	NOT	NA
ART3	NOT	NOT	NOT	NOT	NOT	NOT	NOT	NOT	NOT
BOD1	NOT	NOT	NOT	NA	NA	NA	NA	NOT	NA
POM121C	NA	NA	NA	NA	NA	NA	NA	NA	NA
TNRC6A	NOT	NOT	NOT	NA	NA	NA	NA	NOT	NA
ALDH5A1	NOT	NOT	NOT	NOT	DOWN	NOT	DOWN	NOT	NOT
BFAR	NOT	NOT	NOT	NOT	NOT	NOT	NOT	NOT	NOT
CTH	UP	NOT	NOT	NOT	NOT	NOT	NOT	NOT	NOT
PWWP2A	NOT	NOT	NOT	NOT	NOT	NOT	NOT	NOT	NOT
HPGD	DOWN	NOT	DOWN	DOWN	DOWN	NOT	DOWN	DOWN	NOT
RCBTB1	NOT	NOT	NOT	NOT	NOT	NOT	NOT	NOT	NOT
TTPAL	NOT	NOT	NOT	NOT	NOT	NOT	NOT	NOT	NOT
PAN3	NOT	NOT	NOT	NA	NA	NA	NA	NOT	NA
DCUN1D3	NOT	NOT	NOT	NA	NA	NA	NA	NOT	NA
ULK1	NOT	NOT	NOT	NOT	NOT	NOT	NOT	NOT	NOT
NLK	NOT	NOT	NOT	NOT	NOT	NOT	NOT	NOT	NOT

MAPK6	NOT	NOT	NOT	NOT	NOT	NOT	NOT	NOT	NOT
TMCC1	NOT	NOT	NOT	NOT	NOT	NOT	NOT	NOT	NOT
PTBP3	NOT	NOT	NOT	NOT	NOT	NOT	NOT	NOT	NOT
NUS1	NOT	NOT	NOT	NA	NA	NA	NA	NOT	NA
PTPN13	NOT	NOT	NOT	NOT	NOT	NOT	NOT	NOT	NOT
NAMPT	NOT	NOT	NOT	NOT	NOT	NOT	NOT	NOT	NOT
MEX3B	NOT	NOT	NOT	NA	NA	NA	NA	NOT	NA
NAA15	NOT	NOT	NOT	NOT	NOT	NOT	NOT	NOT	NOT
DDX3X	NOT	NOT	NOT	NOT	NOT	NOT	NOT	NOT	NOT
UBE2H	NOT	NOT	NOT	NOT	NOT	NOT	NOT	NOT	NOT
CDK6	NOT	NOT	NOT	NOT	NOT	NOT	NOT	NOT	NOT
GMDS	NOT	NOT	NOT	NOT	NOT	NOT	NOT	NOT	UP
RCOR1	NOT	NOT	NOT	NOT	NOT	NOT	NOT	NOT	NOT
CDH20	NOT	NOT	NOT	NOT	NOT	NOT	NOT	NOT	NOT
KPNA6	NOT	NOT	NOT	NOT	NOT	NOT	NOT	NOT	NOT
GMFB	NOT	NOT	NOT	NOT	NOT	NOT	NOT	NOT	NOT
MTX2	NOT	NOT	NOT	NOT	NOT	NOT	NOT	NOT	NOT
PHF6	NOT	NOT	NOT	NA	NA	NA	NA	NOT	NA
MAP7	NOT	NOT	NOT	NOT	NOT	NOT	NOT	NOT	NOT
PAWR	NOT	NOT	NOT	NOT	NOT	NOT	NOT	NOT	NOT
SLC2A14	NA	NA	NA	NA	NA	NA	NA	NOT	NA
TBC1D4	NOT	NOT	NOT	NOT	NOT	NOT	NOT	NOT	NOT
MARK1	NOT	NOT	NOT	NOT	NOT	NOT	NOT	NOT	NOT
HOXA9	NA	NA	NA	NA	NA	NA	NA	NA	NA
DOCK4	NOT	NOT	NOT	NOT	NOT	NOT	NOT	NOT	NOT
ASPN	UP	NOT	NOT	NOT	NOT	UP	NOT	NOT	NOT
PTGS2	NOT	NOT	NOT	NOT	NOT	NOT	NOT	NOT	NOT
ZNF148	UP	UP	UP	NOT	NOT	NOT	NOT	NOT	NOT
STAC2	NOT	NOT	NOT	NA	NA	NA	NA	NOT	NA
DEPDC1	UP	UP	UP	NOT	NOT	NOT	NOT	UP	NOT
UGT8	UP	UP	UP	NOT	NOT	NOT	NOT	NOT	NOT
SAMD12	NOT	NOT	NOT	NA	NA	NA	NA	NOT	NA
REST	NOT	NOT	NOT	NOT	NOT	NOT	NOT	NOT	NOT
ABL2	UP	NOT	UP	NOT	NOT	NOT	NOT	NOT	NOT
GPSM1	NOT	NOT	NOT	NA	NA	NA	NA	NOT	NA
WNK3	UP	UP	UP	NA	NA	NA	NA	NOT	NA
MAT2A	NOT	NOT	NOT	NOT	NOT	NOT	NOT	NOT	NOT
MPP6	UP	NOT	NOT	NOT	NOT	NOT	NOT	NOT	NOT
NTN4	NOT	NOT	NOT	NA	NA	NA	NA	NOT	NA
HAS3	NOT	NOT	NOT	NA	NA	NA	NA	NOT	NA
ZFHX4	NOT	NOT	NOT	NOT	NOT	NOT	NOT	DOWN	NOT
MDN1	NOT	NOT	NOT	NOT	NOT	NOT	NOT	NOT	NOT
MXI1	NOT	NOT	NOT	NOT	NOT	NOT	NOT	NOT	NOT
UBE2G1	NOT	NOT	NOT	NOT	NOT	NOT	NOT	NA	NOT
EIF2S1	NOT	NOT	NOT	NOT	NOT	NOT	NOT	NOT	NOT
NUP50	NOT	NOT	NOT	NOT	NOT	NOT	NOT	NOT	NOT
MAP2	NOT	NOT	DOWN	NOT	NOT	NOT	NOT	NOT	NOT

PHF21A	NOT	NOT	NOT	NOT	NOT	NOT	NOT	NOT	NOT
INHBB	NOT	NOT	NOT	NOT	NOT	NOT	NOT	NOT	NOT
USP9X	NOT	NOT	NOT	NOT	NOT	NOT	NOT	NOT	NOT
AGPAT5	UP	NOT	NOT	NOT	NOT	NOT	NOT	NOT	NOT
MAP3K9	NOT	NOT	NOT	NOT	NOT	NOT	NOT	NOT	NOT
LNX2	NOT	NOT	NOT	NA	NA	NA	NA	NOT	NA
CREBZF	UP	NOT	NOT	NOT	NOT	NOT	NOT	NOT	NOT
FAM8A1	NOT	NOT	NOT	NOT	NOT	NOT	NOT	NOT	NOT
ETNK1	NOT	NOT	NOT	NOT	NOT	NOT	NOT	NOT	NOT
ZFC3H1	UP	NOT	NOT	NOT	NOT	NOT	NOT	NOT	NOT
TNPO1	NOT	NOT	NOT	NOT	NOT	NOT	NOT	NOT	NOT
PHF20L1	NOT	NOT	NOT	NOT	NOT	NOT	NOT	NOT	NOT
RHOU	NOT	NOT	NOT	NA	NA	NA	NA	NOT	NA
COL10A1	UP	NOT	NOT	NOT	NOT	NOT	NOT	NOT	NOT
RGS4	UP	NOT	NOT	NOT	NOT	NOT	NOT	NOT	UP
TFAP2A	NOT	NOT	NOT	NOT	NOT	NOT	NOT	NOT	NOT
TBC1D30	UP	UP	UP	NOT	NOT	NOT	NOT	NOT	NOT
BAK1	NOT	NOT	NOT	NOT	NOT	NOT	NOT	UP	NOT
TANC2	NOT	NOT	NOT	NOT	NOT	NOT	NOT	NOT	NOT
CXADR	NOT	NOT	NOT	NOT	NOT	NOT	NOT	NOT	NOT
PEX13	NOT	NOT	NOT	NOT	NOT	NOT	NOT	NOT	NOT
BID	NOT	UP	UP	NOT	NOT	NOT	NOT	NOT	NOT
BOD1L2	NOT	NOT	NOT	NA	NA	NA	NA	NOT	NA
PFKFB3	NOT	NOT	NOT	NOT	NOT	NOT	NOT	NOT	NOT
RLF	NOT	NOT	NOT	NOT	NOT	NOT	NOT	NOT	NOT
JAG1	NOT	NOT	NOT	NOT	NOT	NOT	NOT	NOT	NOT
PATZ1	NOT	NOT	NOT	NOT	NOT	NOT	NOT	NOT	NOT
PIM1	NOT	NOT	NOT	DOWN	NOT	DOWN	NOT	NOT	DOWN
RBM20	DOWN	DOWN	DOWN	NA	NA	NA	NA	NA	NA
TET1	NOT	NOT	NOT	NA	NA	NA	NA	NOT	NA
SH3D19	NOT	NOT	NOT	NA	NA	NA	NA	NOT	NA
RAP2C	NOT	NOT	NOT	NOT	NOT	NOT	NOT	NOT	NOT
SERBP1	NOT	NOT	NOT	NOT	NOT	NOT	NOT	NOT	NOT
TMEM106B	NOT	NOT	NOT	NOT	NOT	NOT	NOT	NOT	NOT
BCR	NOT	NOT	NOT	NOT	NOT	NOT	NOT	NA	NOT
RFX3	NOT	NOT	NOT	NOT	NOT	NOT	NOT	NOT	NOT
PLEKHH1	NOT	NOT	NOT	NA	NA	NA	NA	NOT	NA
BBX	NOT	NOT	NOT	NOT	NOT	NOT	NOT	NOT	NOT
SLC38A2	NOT	NOT	NOT	NOT	NOT	NOT	NOT	NOT	NOT
DDX52	NOT	NOT	NOT	NOT	NOT	NOT	NOT	NOT	NOT
DMRT3	NOT	NOT	NOT	NA	NA	NA	NA	NOT	NA
FBXO28	NOT	NOT	NOT	NOT	NOT	NOT	NOT	NOT	NOT
G2E3	UP	NOT	NOT	NA	NA	NA	NA	NOT	NA
HEPHL1	NA	NA	NA	NA	NA	NA	NA	NOT	NA
CDK2AP1	NOT	NOT	NOT	NOT	NOT	NOT	NOT	NOT	NOT
SFXN1	NOT	NOT	NOT	NOT	NOT	NOT	NOT	NOT	NOT

DUSP5	NOT	NOT	NOT	NOT	NOT	NOT	NOT	NOT	DOWN
GABRA4	NOT	NOT	NOT	NOT	NOT	NOT	NOT	NOT	NOT
DLG4	NOT	NOT	NOT	NOT	NOT	NOT	NOT	NOT	NOT
ANKRD52	NOT	NOT	NOT	NA	NA	NA	NA	NOT	NA
BLOC1S2	NOT	NOT	NOT	NA	NA	NA	NA	NOT	NA
RNF141	NOT	NOT	NOT	NOT	NOT	NOT	NOT	NOT	NOT
ITGB8	NOT	NOT	NOT	NOT	NOT	NOT	NOT	NOT	NOT
PAK2	UP	NOT	NOT	NOT	NOT	NOT	NOT	NOT	NOT
SLC30A7	UP	UP	UP	NA	NA	NA	NA	NOT	NA
HSPA8	NA	NA	NA	NA	NA	NA	NA	NOT	NA
EP400	NOT	NOT	NOT	NOT	NOT	NOT	NOT	NOT	NOT
SLC9A2	NOT	NOT	NOT	NOT	NOT	NOT	NOT	NOT	NOT
ADAMTS19	NOT	NOT	NOT	NA	NA	NA	NA	DOWN	NA
SLC24A4	NOT	NOT	NOT	NA	NA	NA	NA	NOT	NA
STRBP	NOT	NOT	NOT	NA	NA	NA	NA	NOT	NA
RCN2	NOT	NOT	NOT	NOT	NOT	NOT	NOT	NOT	NOT
ZNF710	NOT	NOT	NOT	NOT	DOWN	NOT	NOT	NOT	NOT
DGKH	NOT	NOT	NOT	NOT	NOT	NOT	NOT	NOT	NOT
FAM98B	NOT	NOT	NOT	NA	NA	NA	NA	NOT	NA
RAB3IP	UP	NOT	UP	NA	NA	NA	NA	NOT	NA
GRAMD1C	NOT	NOT	NOT	NOT	NOT	NOT	NOT	NOT	NOT
EPS15	NOT	NOT	NOT	NOT	NOT	NOT	NOT	NOT	NOT
NIPA1	UP	NOT	NOT	NA	NA	NA	NA	NOT	NA
ELAVL2	UP	UP	UP	NOT	NOT	NOT	NOT	NOT	NOT
NIPAL2	DOWN	NOT	NOT	NOT	NOT	NOT	NOT	NOT	NOT
AKAP7	NOT	NOT	NOT	NOT	NOT	NOT	NOT	NOT	NOT
HAO1	NOT	NOT	NOT	NOT	NOT	NOT	NOT	NOT	NOT
SLC33A1	UP	NOT	UP	NOT	NOT	NOT	NOT	NOT	NOT
BTBD7	NOT	NOT	NOT	NOT	NOT	NOT	NOT	NOT	NOT
HELZ	UP	NOT	NOT	NOT	NOT	NOT	NOT	NOT	NOT
HSPA4L	NOT	NOT	NOT	NOT	NOT	NOT	NOT	NOT	NOT
TP53INP1	NOT	NOT	NOT	NA	NA	NA	NA	NOT	NA
PDE4B	UP	NOT	NOT	NOT	NOT	NOT	NOT	NOT	NOT
SACS	UP	NOT	NOT	NOT	NOT	NOT	NOT	NOT	NOT
PON1	NOT	NOT	NOT	NOT	NOT	NOT	NOT	NOT	NOT
CPED1	NOT	NOT	NOT	NOT	NOT	NOT	NOT	DOWN	NOT
PHF3	NOT	NOT	NOT	NOT	NOT	NOT	NOT	NOT	NOT
ADAM10	NOT	NOT	NOT	NOT	NOT	NOT	NOT	NOT	NOT
TMEM68	NOT	NOT	NOT	NA	NA	NA	NA	NOT	NA
A1CF	NOT	NOT	NOT	NOT	NOT	NOT	NOT	NOT	NOT
INTS2	NOT	NOT	NOT	NA	NA	NA	NA	NOT	NA
TMC7	NOT	NOT	NOT	NOT	NOT	NOT	NOT	NOT	NOT
PMAIP1	NOT	NOT	NOT	NOT	NOT	NOT	NOT	NOT	NOT
EIF4G2	NOT	NOT	NOT	NOT	NOT	NOT	NOT	NOT	NOT
PPP3CB	NOT	NOT	NOT	NOT	NOT	NOT	NOT	NOT	NOT

VGLL4	NOT	NOT	NOT	NOT	NOT	NOT	NOT	NOT	NOT
RIPPLY3	UP	UP	UP	NOT	NOT	NOT	NOT	NOT	NOT
TMEM184B	NOT	NOT	NOT	NOT	NOT	NOT	NOT	NOT	NOT
KCNK1	NOT	NOT	NOT	NOT	NOT	NOT	NOT	NOT	NOT
EYA3	NOT	NOT	NOT	NOT	NOT	NOT	NOT	NOT	NOT
MTPN	NOT	NOT	NOT	NA	NA	NA	NA	NA	NA
DCBLD2	UP	UP	UP	NOT	NOT	NOT	NOT	NOT	NOT
TAF9B	NOT	NOT	NOT	NOT	NOT	NOT	NOT	NOT	UP
TMEM86A	NOT	NOT	NOT	NA	NA	NA	NA	NOT	NA
PFKFB2	NOT	NOT	NOT	NOT	NOT	NOT	NOT	NOT	NOT
SKP2	UP	NOT	NOT	UP	NOT	NOT	NOT	NOT	NOT
PAX5	NOT	NOT	NOT	NOT	UP	NOT	UP	NOT	NOT
SLC2A3	UP	NOT	NOT	NOT	NOT	NOT	NOT	NOT	NOT
PHTF2	UP	NOT	NOT	NOT	NOT	NOT	NOT	NOT	NOT
DCAF7	NOT	NOT	NOT	NOT	NOT	NOT	NOT	NOT	NOT
MAP3K2	NOT	NOT	NOT	NOT	NOT	NOT	NOT	NOT	NOT
SLC12A2	NOT	NOT	NOT	NOT	NOT	NOT	NOT	NOT	NOT
GALNT10	NOT	NOT	NOT	NOT	NOT	UP	NOT	NOT	UP
SLC2A13	UP	NOT	NOT	NA	NA	NA	NA	DOWN	NA
CSNK1G1	NOT	NOT	NOT	NOT	NOT	NOT	NOT	NOT	NOT
ATPAF1	NOT	NOT	NOT	NA	NA	NA	NA	NOT	NA
MFAP3	NOT	NOT	NOT	NOT	NOT	NOT	NOT	NOT	NOT
KCNE4	NOT	NOT	NOT	NOT	NOT	NOT	NOT	NOT	NOT
SLC4A4	NOT	NOT	NOT	NOT	NOT	NOT	NOT	NOT	UP
PSD3	NOT	NOT	NOT	NOT	NOT	NOT	NOT	NOT	NOT
RTF1	NOT	NOT	NOT	NOT	NOT	NOT	NOT	NOT	NOT
SLC19A2	NOT	NOT	NOT	DOWN	NOT	DOWN	DOWN	NOT	NOT
PDHX	NOT	NOT	NOT	NOT	NOT	NOT	NOT	NOT	NOT
BRWD1	NOT	NOT	NOT	NOT	NOT	NOT	NOT	NOT	NOT
CHD1	UP	NOT	NOT	NOT	NOT	NOT	NOT	NOT	NOT
HOXD13	UP	NOT	UP	NOT	NOT	NOT	NOT	NOT	NOT
CREB1	NOT	NOT	NOT	NOT	NOT	NOT	NOT	NOT	NOT
PYGO1	NOT	NOT	NOT	NOT	NOT	NOT	NOT	NOT	NOT
HOXC9	NOT	NOT	NOT	NA	NA	NA	NA	NOT	NA
KLHL18	NOT	NOT	NOT	NOT	NOT	NOT	NOT	NOT	NOT
ENPEP	UP	NOT	NOT	NOT	NOT	NOT	NOT	NOT	NOT
CREBBP	NOT	NOT	NOT	NOT	NOT	NOT	NOT	NOT	NOT
CDH2	NOT	NOT	NOT	NOT	NOT	NOT	NOT	NOT	NOT
CDH4	NOT	NOT	NOT	NOT	NOT	NOT	NOT	NOT	NOT
NID1	UP	NOT	NOT	NOT	NOT	NOT	NOT	NOT	UP
TSPYL4	NOT	NOT	NOT	NOT	NOT	NOT	NOT	NOT	NOT
ZNF275	UP	NOT	NOT	NA	NA	NA	NA	NOT	NA
ARMCX2	NOT	NOT	NOT	NOT	NOT	NOT	NOT	NOT	NOT
CACNB4	NOT	NOT	NOT	NOT	NOT	NOT	NOT	NOT	NOT
GTF3C2	NOT	NOT	NOT	NOT	NOT	NOT	NOT	NOT	NOT
FAM49B	UP	NOT	NOT	NOT	NOT	NOT	NOT	NOT	NOT

SALL1	NOT	NOT	NOT	NOT	NOT	NOT	NOT	NOT	NOT
WNK1	NOT	NOT	NOT	NOT	NOT	NOT	NOT	NOT	DOWN
HECTD4	NOT	NOT	NOT	NOT	NOT	NOT	NOT	NOT	NOT
SLC45A4	UP	NOT	UP	NA	NA	NA	NA	NOT	NA
FAXC	NOT	NOT	NOT	NA	NA	NA	NA	NOT	NA
PLEKHG1	NOT	NOT	NOT	NA	NA	NA	NA	NOT	NA
GRIN2A	NOT	NOT	NOT	NOT	NOT	NOT	NOT	NOT	NOT
BCL7B	NOT	NOT	NOT	NOT	NOT	NOT	NOT	NOT	NOT
ARL5B	NOT	NOT	NOT	NA	NA	NA	NA	NOT	NA
CCNJ	UP	NOT	NOT	NOT	NOT	NOT	NOT	NOT	NOT
CREBRF	NOT	NOT	NOT	NA	NA	NA	NA	NOT	NA
PHAX	NOT	NOT	NOT	NA	NA	NA	NA	NOT	NA
GSR	NOT	NOT	NOT	NOT	NOT	NOT	NOT	NOT	NOT
COMMD8	NOT	NOT	NOT	NOT	NOT	NOT	NOT	NOT	NOT
TNKS2	NOT	NOT	NOT	NOT	NOT	NOT	NOT	NOT	NOT
RFK	NOT	NOT	NOT	NOT	NOT	NOT	NOT	NOT	NOT
TAOK1	NOT	NOT	NOT	NOT	NOT	NOT	NOT	NOT	NOT
ESR1	DOWN	DOWN	DOWN	DOWN	DOWN	NOT	DOWN	DOWN	NOT
OAF	NOT	NOT	NOT	NA	NA	NA	NA	NOT	NA
GNPNAT1	NOT	NOT	NOT	NA	NA	NA	NA	NOT	NA
DFFB	NOT	NOT	NOT	NOT	NOT	NOT	NOT	NOT	NOT
EPHA5	NOT	NOT	NOT	NOT	NOT	NOT	NOT	NOT	NOT
MAEA	NOT	NOT	NOT	NOT	NOT	NOT	NOT	NOT	NOT
MKNK2	NOT	NOT	NOT	NOT	NOT	NOT	NOT	NOT	NOT
NCEH1	UP	NOT	UP	NA	NA	NA	NA	NOT	NA
C1GALT1	NOT	NOT	NOT	NOT	NOT	NOT	NOT	NOT	NOT
RB1	NOT	NOT	NOT	NOT	NOT	NOT	NOT	NOT	NOT
ZDHHC18	NOT	NOT	NOT	NOT	NOT	NOT	NOT	NOT	NOT
ARPC3	NOT	NOT	NOT	NOT	NOT	NOT	NOT	NOT	NOT
LPP	NOT	NOT	NOT	NOT	NOT	NOT	NOT	NOT	NOT
LEF1	UP	NOT	UP	NOT	NOT	NOT	NOT	NOT	NOT
MMP16	NOT	NOT	NOT	NOT	NOT	NOT	NOT	NOT	NOT
LARP4	NOT	NOT	NOT	NOT	NOT	NOT	NOT	NOT	NOT
NACC2	NOT	NOT	NOT	NOT	NOT	NOT	NOT	NOT	NOT
CDC6	UP	UP	UP	UP	NOT	NOT	NOT	UP	UP
CPM	DOWN	DOWN	DOWN	NOT	NOT	NOT	NOT	NOT	NOT
ANKIB1	UP	NOT	NOT	NA	NA	NA	NA	NOT	NA
GRB10	NOT	NOT	NOT	NOT	NOT	NOT	NOT	NOT	NOT
HOMER1	NOT	NOT	NOT	NOT	NOT	NOT	NOT	NOT	NOT
HMGA2	NOT	NOT	NOT	NOT	NOT	NOT	NOT	NOT	UP
SH3PXD2A	NOT	NOT	NOT	NOT	NOT	NOT	NOT	NOT	NOT
YTHDF3	NOT	NOT	NOT	NOT	NOT	NOT	NOT	NOT	NOT
JARID2	NOT	NOT	NOT	NOT	NOT	NOT	NOT	NOT	NOT
ZNF469	UP	NOT	NOT	NA	NA	NA	NA	NOT	NA
PELI2	UP	NOT	UP	NOT	NOT	NOT	NOT	NOT	NOT
RANBP9	NOT	NOT	NOT	DOWN	NOT	NOT	NOT	NOT	DOWN

C2CD5	UP	NOT	NOT	NOT	NOT	NOT	NOT	NOT	NOT
UBR1	NOT	NOT	NOT	NA	NA	NA	NA	NOT	NA
PGRMC2	NOT	NOT	NOT	DOWN	NOT	NOT	NOT	NOT	NOT
ARHGAP21	UP	NOT	NOT	NA	NA	NA	NA	NOT	NA
BHLHE40	NOT	NOT	NOT	NOT	NOT	NOT	NOT	NOT	NOT
IPPK	NOT	NOT	NOT	NOT	NOT	NOT	NOT	NOT	NOT
UBE3A	NOT	NOT	NOT	NOT	NOT	NOT	NOT	NOT	NOT
SOCS6	NOT	NOT	NOT	NOT	NOT	NOT	NOT	NOT	NOT
PAG1	UP	UP	UP	NA	NA	NA	NA	NOT	NA
ZNF106	NOT	NOT	NOT	NOT	NOT	NOT	NOT	NOT	NOT
TCF12	NOT	NOT	NOT	NOT	NOT	NOT	NOT	NOT	NOT
USP37	UP	NOT	UP	NA	NA	NA	NA	NOT	NA
PCDH18	NOT	NOT	NOT	NA	NA	NA	NA	DOWN	NA
EP300	NOT	NOT	NOT	NOT	NOT	NOT	NOT	NOT	NOT
GOLGA3	NOT	NOT	NOT	NOT	NOT	NOT	NOT	NOT	NOT
DDX17	NOT	NOT	NOT	NOT	NOT	NOT	NOT	NOT	NOT
ZBTB10	NOT	NOT	NOT	NOT	NOT	NOT	NOT	NOT	NOT
MIB1	NOT	NOT	NOT	NA	NA	NA	NA	NOT	NA
PRR5L	NOT	NOT	NOT	NOT	NOT	NOT	NOT	NOT	NOT
CDK13	NOT	NOT	NOT	NOT	NOT	NOT	NOT	NOT	NOT
KCNQ4	NOT	NOT	NOT	NOT	NOT	NOT	NOT	NOT	NOT
ACAP2	NOT	NOT	NOT	NOT	NOT	NOT	NOT	NOT	NOT
LSM11	NOT	NOT	NOT	NA	NA	NA	NA	NOT	NA
GMNC	NOT	NOT	NOT	NA	NA	NA	NA	NA	NA
ARHGAP26	NOT	NOT	NOT	NOT	NOT	UP	NOT	NOT	NOT
FAM199X	NOT	NOT	NOT	NA	NA	NA	NA	NOT	NA
OTUD1	NOT	NOT	NOT	NA	NA	NA	NA	NOT	NA
UBE2E2	NOT	NOT	NOT	NA	NA	NA	NA	NOT	NA
UBE2K	NOT	NOT	NOT	NOT	NOT	NOT	NOT	NOT	NOT
DYRK1A	NOT	NOT	NOT	NOT	NOT	NOT	NOT	NOT	NOT
RTN1	NOT	NOT	NOT	NOT	NOT	NOT	NOT	NOT	NOT
MYCBP	NA	NA	NA	NA	NA	NA	NA	NA	NA
EPHA7	NOT	NOT	NOT	NOT	NOT	NOT	NOT	DOWN	NOT
SCML4	NOT	NOT	NOT	NA	NA	NA	NA	NOT	NA
SLC1A1	NOT	NOT	NOT	NOT	NOT	NOT	NOT	NOT	NOT
SLC26A4	DOWN	NOT	NOT	NOT	NOT	NOT	NOT	DOWN	UP
SESN3	NOT	NOT	NOT	NA	NA	NA	NA	NOT	NA
EAF1	NOT	NOT	NOT	NA	NA	NA	NA	NOT	NA
CSTF2	NOT	NOT	NOT	NOT	NOT	NOT	NOT	NOT	NOT
SBNO1	NOT	NOT	NOT	NOT	NOT	NOT	NOT	NOT	NOT
ZNF385B	NOT	NOT	NOT	NA	NA	NA	NA	NOT	NA
ERLIN1	NOT	NOT	NOT	NOT	NOT	NOT	NOT	NOT	NOT
CELSR1	NOT	NOT	NOT	NOT	NOT	NOT	NOT	NOT	NOT
CNR1	NOT	NOT	NOT	NOT	NOT	NOT	NOT	NOT	NOT
SLC25A20	NOT	NOT	NOT	NOT	NOT	NOT	NOT	NOT	NOT

SH3RF1	NOT	NOT	NOT	NA	NA	NA	NA	NOT	NA
CELF2	NOT	NOT	NOT	NOT	NOT	NOT	NOT	NOT	NOT
USP27X	NOT	NOT	NOT	NOT	NOT	NOT	NOT	NOT	NOT
SLC16A6	DOWN	NOT	NOT	DOWN	NOT	NOT	NOT	NOT	DOWN
KLF4	DOWN	NOT	NOT	DOWN	DOWN	DOWN	DOWN	DOWN	DOWN
UBE2W	NOT	NOT	NOT	NOT	NOT	NOT	NOT	NOT	NOT
OSBPL2	NOT	NOT	NOT	NOT	NOT	NOT	NOT	NOT	NOT
EDEM3	NOT	NOT	NOT	NOT	NOT	NOT	NOT	NOT	NOT
SAR1B	NOT	NOT	NOT	NOT	NOT	NOT	NOT	NOT	NOT
SEMA6D	NOT	NOT	NOT	NOT	NOT	NOT	NOT	NOT	NOT
RAB11A	NOT	NOT	NOT	NOT	NOT	NOT	NOT	NOT	NOT
HIPK1	NOT	NOT	NOT	NOT	NOT	NOT	NOT	NOT	NOT
CHSY1	NOT	NOT	NOT	NOT	NOT	NOT	NOT	NOT	NOT
VDAC1	NOT	NOT	NOT	NOT	NOT	NOT	NOT	NOT	NOT
TSPAN5	NOT	NOT	NOT	NOT	NOT	NOT	NOT	NOT	NOT
PLXNA2	NOT	NOT	NOT	NOT	NOT	NOT	NOT	NOT	NOT
TM4SF18	NOT	NOT	NOT	NA	NA	NA	NA	NOT	NA
RDH14	NA	NA	NA	NA	NA	NA	NA	NA	NA
KIAA1549L	NOT	NOT	NOT	NOT	NOT	NOT	NOT	NOT	NOT
PPP2R5A	NOT	NOT	NOT	NOT	NOT	NOT	NOT	NOT	NOT
PPP1R3D	NOT	NOT	NOT	NOT	NOT	NOT	NOT	NOT	NOT
PAK1	NOT	NOT	NOT	NOT	NOT	NOT	NOT	NOT	NOT
DRAM1	UP	NOT	NOT	NOT	NOT	NOT	NOT	NOT	NOT
TWF1	NOT	NOT	NOT	NOT	NOT	NOT	NOT	NOT	NOT
MINPP1	UP	NOT	NOT	NOT	NOT	NOT	NOT	NOT	NOT
COL22A1	NOT	NOT	NOT	NA	NA	NA	NA	NOT	NA
RAB21	NOT	NOT	NOT	NOT	NOT	NOT	NOT	NOT	NOT
MED13L	NOT	NOT	NOT	NOT	NOT	NOT	NOT	NOT	NOT
C18orf25	NOT	NOT	NOT	NOT	NOT	NOT	NOT	NOT	DOWN
KMT2C	NOT	NOT	NOT	NA	NA	NA	NA	NOT	NA
MNX1	NOT	NOT	NOT	NOT	UP	NOT	NOT	NOT	NOT
GTF2A1	NOT	NOT	NOT	NOT	NOT	NOT	NOT	NOT	NOT
USP25	NOT	NOT	NOT	NOT	NOT	NOT	NOT	NOT	NOT
RCN1	NOT	NOT	NOT	UP	NOT	NOT	NOT	NOT	NOT
IPMK	NOT	NOT	NOT	NA	NA	NA	NA	NOT	NA
ZCCHC24	DOWN	DOWN	DOWN	NOT	NOT	NOT	NOT	NOT	NOT
RYK	NOT	NOT	NOT	NOT	NOT	NOT	NOT	NOT	NOT
ACER3	NOT	NOT	NOT	NA	NA	NA	NA	NOT	NA
EIF5	NOT	NOT	NOT	NOT	NOT	NOT	NOT	NOT	NOT
WNT5A	NOT	NOT	NOT	NOT	DOWN	NOT	DOWN	NOT	NOT
NFE2L3	NOT	UP	UP	NOT	NOT	NOT	NOT	NOT	NOT
LARP4B	NOT	NOT	NOT	NOT	NOT	NOT	NOT	NOT	NOT
COL1A2	NOT	NOT	NOT	NOT	NOT	NOT	NOT	NOT	NOT
CEBPG	UP	NOT	NOT	NOT	NOT	NOT	NOT	NOT	NOT
CPEB2	DOWN	NOT	NOT	NA	NA	NA	NA	DOWN	NA
TMEM260	NOT	NOT	NOT	NOT	NOT	NOT	NOT	NOT	UP

CTDSP2	NOT	NOT	NOT	NOT	NOT	NOT	NOT	NOT	NOT
FAM160A1	DOWN	DOWN	DOWN	NA	NA	NA	NA	NOT	NA
SLC22A23	NOT	NOT	NOT	NA	NA	NA	NA	NOT	NA
YPEL1	NOT	NOT	NOT	NOT	NOT	NOT	NOT	NOT	NOT
KCNH7	NOT	NOT	NOT	NA	NA	NA	NA	NOT	NA
LMLN	NOT	NOT	NOT	NA	NA	NA	NA	NOT	NA
MATR3	NOT	NOT	NOT	NA	NA	NA	NA	NA	NA
AKIRIN1	NOT	NOT	NOT	NOT	NOT	NOT	NOT	NOT	NOT
WIPF2	NOT	NOT	NOT	NOT	NOT	NOT	NOT	NOT	NOT
CDKN1C	NOT	NOT	NOT	NOT	NOT	NOT	NOT	NOT	NOT
MFSD6	NOT	NOT	NOT	NOT	NOT	NOT	NOT	NOT	NOT
FUT9	NOT	NOT	NOT	NOT	NOT	NOT	NOT	NOT	NOT
TFAP2E	NOT	NOT	NOT	NA	NA	NA	NA	NOT	NA
HSPA13	UP	UP	UP	NOT	NOT	NOT	NOT	NOT	NOT
ZNF516	NOT	NOT	NOT	NOT	NOT	NOT	NOT	DOWN	NOT
PPM1H	NOT	NOT	NOT	NOT	NOT	NOT	NOT	NOT	UP
PLCL1	NOT	NOT	NOT	NOT	NOT	NOT	NOT	NOT	NOT
METAP2	NOT	NOT	NOT	NOT	NOT	NOT	NOT	NOT	NOT
ETF1	NOT	NOT	NOT	NOT	NOT	NOT	NOT	NOT	NOT
ZEB2	UP	NOT	NOT	NOT	NOT	NOT	NOT	DOWN	NOT
DIDO1	NOT	NOT	NOT	NOT	NOT	NOT	NOT	NOT	NOT
DNAJB5	NOT	NOT	NOT	NOT	NOT	NOT	NOT	NOT	NOT
GALNT7	NOT	NOT	NOT	NOT	NOT	UP	NOT	NOT	UP
TMEM248	NOT	NOT	NOT	NOT	NOT	NOT	NOT	NOT	NOT
TBL1XR1	NOT	NOT	NOT	NOT	NOT	NOT	NOT	NOT	NOT
SLC35E2B	NOT	NOT	NOT	NA	NA	NA	NA	NA	NA
USP3	NOT	NOT	NOT	NOT	NOT	NOT	NOT	NOT	NOT
UBE2D1	NOT	NOT	NOT	NOT	NOT	NOT	NOT	NOT	NOT
SOSTDC1	DOWN	DOWN	DOWN	DOWN	DOWN	DOWN	DOWN	DOWN	DOWN
B4GALT1	NOT	NOT	NOT	NOT	NOT	NOT	NOT	NOT	NOT
MLLT3	NOT	NOT	NOT	NOT	NOT	NOT	NOT	NOT	NOT
CAPZA1	NOT	NOT	NOT	NOT	NOT	NOT	NOT	NOT	NOT
IPO7	NOT	NOT	NOT	NOT	NOT	NOT	NOT	NOT	NOT
LINGO1	NOT	NOT	NOT	NA	NA	NA	NA	NOT	NA
REEP4	NOT	NOT	NOT	NOT	NOT	NOT	NOT	NOT	NOT
PDE7A	UP	NOT	NOT	NA	NA	NA	NA	NOT	NA
ARL4C	NOT	NOT	NOT	NOT	DOWN	NOT	NOT	NOT	NOT
RAB31	NOT	NOT	NOT	NOT	NOT	NOT	NOT	NOT	NOT
PDE4D	NOT	NOT	NOT	NOT	NOT	NOT	NOT	NOT	NOT
PURA	NOT	NOT	NOT	NOT	NOT	NOT	NOT	NOT	NOT
CCDC50	UP	NOT	NOT	NA	NA	NA	NA	NOT	NA
FAM172A	NOT	NOT	NOT	NOT	NOT	NOT	NOT	NOT	NOT
FBXO42	NOT	NOT	NOT	NOT	NOT	NOT	NOT	NOT	NOT
HECTD3	NOT	NOT	NOT	NOT	NOT	NOT	NOT	NOT	NOT
NEK6	NOT	NOT	NOT	NA	NA	NA	NA	NOT	NA
CCND2	NOT	DOWN	NOT	NOT	DOWN	NOT	DOWN	NOT	NOT

ADAM12	UP	NOT	NOT	NOT	NOT	NOT	NOT	NOT	NOT
RAP1A	NOT	NOT	NOT	NOT	NOT	NOT	NOT	NOT	NOT
ST8SIA4	NOT	NOT	NOT	NOT	NOT	NOT	NOT	NOT	NOT
PPP1R15B	NOT	NOT	NOT	NA	NA	NA	NA	NOT	NA
ZNRF3	NOT	NOT	NOT	NA	NA	NA	NA	NOT	NA
GPC4	NOT	NOT	NOT	NOT	NOT	NOT	NOT	NOT	NOT
RFX7	NOT	NOT	NOT	NOT	NOT	NOT	NOT	NOT	NOT
ROCK1	NOT	NOT	NOT	NOT	NOT	NOT	NOT	NOT	NOT
TMEM135	NOT	NOT	NOT	NOT	NOT	NOT	NOT	NOT	NOT
B4GALT4	NOT	NOT	NOT	NOT	UP	NOT	UP	NOT	NOT
NAGPA	NOT	NOT	NOT	NOT	NOT	NOT	NOT	NOT	NOT
ATXN7	NOT	NOT	NOT	NOT	NOT	NOT	NOT	NOT	NOT
PNRC1	NOT	NOT	NOT	NOT	NOT	NOT	NOT	NOT	NOT
LPAR3	NOT	NOT	NOT	NOT	NOT	NOT	NOT	NOT	NOT
CARM1	NA	NA	NA	NA	NA	NA	NA	NOT	NA
TMEM33	NOT	NOT	NOT	NOT	NOT	NOT	NOT	NOT	NOT
GATA4	NOT	NOT	NOT	NOT	NOT	NOT	NOT	NOT	NOT
UBTD2	UP	NOT	UP	NA	NA	NA	NA	NOT	NA
PFDN4	NOT	NOT	NOT	NOT	NOT	NOT	NOT	NOT	NOT
FAM118B	NOT	NOT	NOT	NA	NA	NA	NA	NOT	NA
TMEM64	NOT	NOT	NOT	NA	NA	NA	NA	NA	NA
NCAM2	NOT	NOT	NOT	NOT	NOT	NOT	NOT	NOT	NOT
RANBP10	NOT	NOT	NOT	NOT	NOT	NOT	NOT	NOT	NOT
CTNNBIP1	NOT	NOT	NOT	NOT	NOT	NOT	NOT	NOT	NOT
MSTN	NOT	NOT	NOT	NOT	NOT	NOT	NOT	NOT	NOT
HAPLN1	UP	NOT	NOT	NOT	NOT	NOT	NOT	NOT	NOT
COL11A1	NOT	NOT	NOT	NOT	NOT	NOT	NOT	NOT	NOT
DAB2	NOT	NOT	NOT	NOT	NOT	NOT	NOT	NOT	NOT
KCTD18	NOT	NOT	NOT	NA	NA	NA	NA	NOT	NA
DERL2	NOT	NOT	NOT	NOT	NOT	NOT	NOT	NOT	NOT
GRSF1	NOT	NOT	NOT	NOT	NOT	NOT	NOT	NOT	NOT
UBE2J1	NOT	NOT	NOT	NOT	NOT	NOT	NOT	NOT	NOT
MYPOP	UP	NOT	NOT	NA	NA	NA	NA	NOT	NA
TMEM178B	NOT	NOT	NOT	NA	NA	NA	NA	NA	NA
RAP1B	NOT	NOT	NOT	NOT	NOT	NOT	NOT	NOT	NOT
ARL6IP6	UP	UP	UP	NA	NA	NA	NA	NOT	NA
RP2	NOT	NOT	NOT	NOT	NOT	NOT	NOT	NOT	NOT
DMXL1	NOT	NOT	NOT	NOT	NOT	NOT	NOT	NOT	NOT
LMAN1	NOT	NOT	NOT	NOT	NOT	NOT	NOT	NOT	NOT
ZFHX3	NOT	NOT	NOT	NOT	NOT	NOT	NOT	NOT	NOT
LTBP1	NOT	NOT	NOT	NOT	NOT	NOT	NOT	DOWN	NOT

Table S8c. Expression profiles of predicted target genes of hsa-miR-146b-5p in GEO datasets.

Target genes	GSE63514(CvsN)	GSE63514(HvsN)	GSE63514(HCvsN)	GSE9750(CvsN)	GSE7803(CvsN)	GSE7803(HvsN)	GSE7803(HCvsN)	GSE52903(CvsN)	GSE27678(CvsN)
TRAF6	NOT	NOT	NOT	NOT	NOT	NOT	NOT	NOT	NOT
IRAK1	NOT	NOT	NOT	NOT	NOT	NOT	NOT	NOT	NOT
DCAF12	NOT	NOT	NOT	NA	NA	NA	NA	NOT	NA
IGSF1	NOT	NOT	NOT	NOT	NOT	NOT	NOT	NA	NOT
HIPK3	NOT	NOT	NOT	NOT	NOT	NOT	NOT	NOT	NOT
LCOR	NOT	NOT	NOT	NA	NA	NA	NA	NOT	NA
KLF7	UP	NOT	NOT	NOT	NOT	NOT	NOT	NOT	NOT
ZBTB2	UP	NOT	NOT	NA	NA	NA	NA	NOT	NA
ZNF367	UP	UP	UP	NA	NA	NA	NA	UP	NA
MMP16	NOT	NOT	NOT	NOT	NOT	NOT	NOT	NOT	NOT
SIAH2	UP	NOT	NOT	NOT	NOT	NOT	NOT	NOT	NOT
CD80	NOT	NOT	NOT	NOT	NOT	NOT	NOT	NOT	NOT
SLC10A3	NOT	NOT	NOT	NOT	NOT	NOT	NOT	NOT	NOT
HNRNPD	NOT	NOT	NOT	NOT	NOT	NOT	NOT	NOT	NOT
ZDHHC13	NOT	NOT	NOT	NOT	NOT	NOT	NOT	NOT	NOT
RFX7	NOT	NOT	NOT	NOT	NOT	NOT	NOT	NOT	NOT
FBXW2	NOT	NOT	NOT	NOT	NOT	NOT	NOT	NOT	NOT
ERBB4	NOT	NOT	NOT	NOT	NOT	NOT	NOT	DOWN	UP
ABL2	UP	NOT	UP	NOT	NOT	NOT	NOT	NOT	NOT
BCORL1	NOT	NOT	NOT	NOT	NOT	NOT	NOT	NOT	NOT
MYBL1	UP	UP	UP	NOT	NOT	NOT	NOT	NOT	NOT
EIF4G2	NOT	NOT	NOT	NOT	NOT	NOT	NOT	NOT	NOT
SEC23IP	NOT	NOT	NOT	NOT	NOT	NOT	NOT	NOT	NOT
WWC2	NOT	NOT	NOT	NA	NA	NA	NA	NOT	NA
RARB	NOT	NOT	NOT	NOT	NOT	NOT	NOT	NOT	UP
APPL1	NOT	NOT	NOT	NOT	NOT	NOT	NOT	NOT	NOT
NUMB	NOT	NOT	NOT	NA	NA	NA	NA	NOT	NA
ZNRF3	NOT	NOT	NOT	NA	NA	NA	NA	NOT	NA
CDKN2AIP	NOT	NOT	NOT	NOT	NOT	NOT	NOT	NOT	NOT
ZNF652	NOT	NOT	NOT	NOT	NOT	NOT	NOT	NOT	NOT
LRP2	NOT	NOT	NOT	NOT	NOT	NOT	NOT	NOT	NOT
ROBO1	NOT	NOT	NOT	NOT	NOT	NOT	NOT	NOT	NOT
DDHD1	UP	NOT	NOT	NA	NA	NA	NA	NOT	NA
C16orf72	NOT	NOT	NOT	NOT	NOT	NOT	NOT	NOT	NOT
SYT1	NOT	NOT	NOT	NOT	NOT	NOT	NOT	NOT	NOT
NOVA1	DOWN	NOT	NOT	NOT	NOT	NOT	NOT	DOWN	NOT
ZNF512B	NOT	NOT	NOT	NOT	NOT	NOT	NOT	NA	NOT
TMEM120B	NOT	NOT	NOT	NOT	NOT	NOT	NOT	NOT	NOT
PIP5K1B	NOT	NOT	NOT	NOT	NOT	NOT	NOT	NOT	UP
LFNG	NOT	NOT	NOT	NOT	NOT	NOT	NOT	NOT	NOT
PPP1R11	NOT	NOT	NOT	NOT	NOT	NOT	NOT	NOT	NOT
ZNF532	NOT	NOT	NOT	NOT	UP	UP	UP	NOT	NOT
STRBP	NOT	NOT	NOT	NA	NA	NA	NA	NOT	NA

AAK1	NOT	NOT	NOT	NOT	NOT	NOT	NOT	NOT	NOT
ACKR2	NOT	NOT	NOT	NOT	NOT	NOT	NOT	NOT	NOT
RIMS2	NOT	NOT	NOT	NOT	NOT	NOT	NOT	NOT	NOT
LRRC15	UP	NOT	NOT	NOT	NOT	NOT	NOT	NOT	NOT
XKR4	NOT	NOT	NOT	NA	NA	NA	NA	NOT	NA
CDS1	NOT	NOT	NOT	NOT	NOT	NOT	NOT	NOT	NOT
GALNT10	NOT	NOT	NOT	NOT	NOT	UP	NOT	NOT	UP
VASN	NOT	NOT	NOT	NA	NA	NA	NA	NOT	NA
RABGAP1	NOT	NOT	NOT	NOT	NOT	NOT	NOT	NOT	NOT
TBC1D20	NOT	NOT	NOT	NA	NA	NA	NA	NOT	NA
PTGFRN	UP	NOT	NOT	NA	NA	NA	NA	NOT	NA
SORT1	DOWN	NOT	NOT	NOT	NOT	NOT	NOT	NOT	NOT
GDNF	NOT	NOT	NOT	NOT	NOT	NOT	NOT	NOT	NOT
SCN3B	NOT	NOT	NOT	NOT	NOT	NOT	NOT	NOT	NOT
ARMC8	NOT	NOT	NOT	NOT	NOT	NOT	NOT	NOT	NOT
ESYT2	NOT	NOT	NOT	NA	NA	NA	NA	NOT	NA
SAMD8	NOT	NOT	NOT	NA	NA	NA	NA	NOT	NA
CARD10	NOT	NOT	NOT	NOT	NOT	NOT	NOT	NOT	NOT
CCDC6	NOT	NOT	NOT	NOT	NOT	NOT	NOT	NOT	NOT
FBXO28	NOT	NOT	NOT	NOT	NOT	NOT	NOT	NOT	NOT
RPA3	NOT	UP	UP	NOT	NOT	NOT	NOT	NOT	NOT
ZFYVE1	NOT	NOT	NOT	NA	NA	NA	NA	NOT	NA
BMPR1A	NOT	NOT	NOT	NOT	NOT	NOT	NOT	NOT	NOT
PRKAA2	NOT	NOT	NOT	NOT	NOT	NOT	NOT	NOT	NOT
SRSF12	NOT	NOT	NOT	NA	NA	NA	NA	NOT	NA
MYT1	NOT	NOT	NOT	NOT	NOT	NOT	NOT	NOT	NOT
USP3	NOT	NOT	NOT	NOT	NOT	NOT	NOT	NOT	NOT
PRX	NOT	NOT	NOT	NOT	NOT	NOT	NOT	NOT	NOT
ZNRF2	NOT	NOT	NOT	NA	NA	NA	NA	NOT	NA
SEMA3G	NOT	NOT	NOT	NOT	NOT	NOT	NOT	NOT	NOT
PTPRA	NOT	NOT	NOT	NOT	NOT	NOT	NOT	NA	NOT
GRID1	NOT	NOT	NOT	NA	NA	NA	NA	NOT	NA

Table S8d. Expression profiles of predicted target genes of hsa-miR-191-5p in GEO datasets.

Target genes	GSE63514(CvsN)	GSE63514(HvsN)	GSE63514(HCvsN)	GSE9750(CvsN)	GSE7803(CvsN)	GSE7803(HvsN)	GSE7803(HCvsN)	GSE52903(CvsN)	GSE27678(CvsN)
MAPRE2	NOT	NOT	NOT	NOT	NOT	NOT	NOT	NOT	NOT
PLCD1	NOT	NOT	NOT	NOT	NOT	NOT	NOT	NOT	NOT
TMOD2	NOT	NOT	NOT	NOT	NOT	NOT	NOT	NOT	NOT
NRCAM	NOT	NOT	NOT	NOT	NOT	NOT	NOT	NOT	NOT
AMMECR1	NOT	NOT	NOT	NOT	NOT	NOT	NOT	NOT	NOT
TJP1	NOT	NOT	NOT	NOT	NOT	NOT	NOT	NOT	NOT
ZCCHC14	NOT	NOT	NOT	NOT	NOT	NOT	NOT	NOT	NOT
ZBTB34	NOT	NOT	NOT	NA	NA	NA	NA	NOT	NA
SATB1	DOWN	NOT	NOT	NOT	NOT	NOT	NOT	NOT	NOT
NEURL4	NOT	NOT	NOT	NA	NA	NA	NA	NOT	NA

CDK6	NOT	NOT	NOT	NOT	NOT	NOT	NOT	NOT	NOT
MAPRE3	NOT	NOT	NOT	NOT	NOT	NOT	NOT	NOT	NOT
WIZ	DOWN	DOWN	DOWN	NOT	NOT	NOT	NOT	NOT	NOT
BDNF	NOT	NOT	NOT	NOT	NOT	NOT	NOT	NOT	NOT
TAF5	UP	UP	UP	NOT	NOT	NOT	NOT	NOT	NOT

Table S8e. Expression profiles of predicted target genes of hsa-miR-484 in GEO datasets.

Target genes	GSE63514(CvsN)	GSE63514(HvsN)	GSE63514(HCvsN)	GSE9750(CvsN)	GSE7803(CvsN)	GSE7803(HvsN)	GSE7803(HCvsN)	GSE52903(CvsN)	GSE27678(CvsN)
PTGER4	NOT	NOT	NOT	NOT	DOWN	DOWN	DOWN	NOT	DOWN
CREB3L3	NOT	NOT	NOT	NA	NA	NA	NA	NOT	NA
MYCBP2	NOT	NOT	NOT	NOT	NOT	NOT	NOT	NOT	NOT
AVL9	NOT	NOT	NOT	NOT	NOT	NOT	NOT	NOT	NOT
SORBS2	DOWN	NOT	DOWN	NOT	NOT	NOT	NOT	DOWN	NOT
SLC20A2	NOT	NOT	NOT	NOT	NOT	NOT	NOT	NOT	NOT
PCDH19	NOT	NOT	NOT	NA	NA	NA	NA	NOT	NA
HIVEP2	NOT	NOT	NOT	NOT	NOT	NOT	NOT	NOT	NOT
HK2	NOT	NOT	NOT	NOT	NOT	NOT	NOT	UP	NOT
DACH1	NOT	NOT	NOT	NOT	DOWN	NOT	NOT	DOWN	NOT
LAT	NOT	NOT	NOT	NOT	NOT	NOT	NOT	NOT	NOT
MEN1	NOT	NOT	NOT	NOT	NOT	NOT	NOT	NOT	NOT
SNRNP200	NOT	NOT	NOT	NA	NA	NA	NA	NOT	NA
DCBLD2	UP	UP	UP	NOT	NOT	NOT	NOT	NOT	NOT
C17orf112	NA	NA	NA	NA	NA	NA	NA	NA	NA
CYB561D1	NOT	NOT	NOT	NA	NA	NA	NA	NOT	NA
TRAT1	UP	NOT	NOT	NOT	NOT	NOT	NOT	NOT	NOT
MGAT5	NOT	NOT	NOT	NOT	NOT	NOT	NOT	NOT	NOT
SNN	NOT	NOT	NOT	NOT	NOT	NOT	NOT	NOT	NOT
FGF1	NOT	NOT	NOT	NOT	NOT	NOT	NOT	NOT	NOT
TSGA10	NOT	NOT	NOT	NOT	NOT	NOT	NOT	NOT	NOT
ANAPC7	NOT	NOT	NOT	NA	NA	NA	NA	NOT	NA
KIAA1549	NOT	NOT	NOT	NA	NA	NA	NA	NOT	NA
IL21R	NOT	NOT	NOT	NOT	NOT	NOT	NOT	NOT	NOT
RIMS2	NOT	NOT	NOT	NOT	NOT	NOT	NOT	NOT	NOT
KCNJ2	NOT	NOT	NOT	NOT	NOT	NOT	NOT	NOT	NOT
APLN	NOT	UP	UP	NA	NA	NA	NA	NOT	NA
XPO4	NOT	NOT	NOT	NOT	NOT	NOT	NOT	NOT	NOT
ADAM33	NOT	NOT	NOT	NA	NA	NA	NA	NOT	NA
TOPBP1	UP	UP	UP	UP	UP	NOT	UP	UP	NOT
S100PBP	UP	UP	UP	NOT	NOT	NOT	NOT	NOT	NOT
ACVR1B	NOT	NOT	NOT	NOT	NOT	NOT	NOT	NOT	NOT
IFNAR1	NOT	NOT	NOT	NOT	NOT	NOT	NOT	NOT	NOT
MTF2	UP	UP	UP	NOT	UP	NOT	NOT	NOT	NOT
SERTAD1	NOT	NOT	NOT	NA	NA	NA	NA	NOT	NA
ZNF37A	NOT	NOT	NOT	NOT	NOT	NOT	NOT	NOT	NOT

FOSL2	NOT	NOT	NOT	NOT	NOT	NOT	NOT	NOT	NOT
KMT2A	NOT	NOT	NOT	NOT	NOT	NOT	NOT	NOT	NOT
PLEKHG4	UP	UP	UP	NA	NA	NA	NA	NOT	NA
NOL9	NOT	NOT	NOT	NOT	NOT	NOT	NOT	NOT	NOT
XKR9	NA	NA	NA	NA	NA	NA	NA	NOT	NA
LRPAP1	NOT	NOT	NOT	NOT	NOT	NOT	NOT	NOT	NOT
STRN	NOT	NOT	NOT	NOT	NOT	NOT	NOT	NOT	NOT
HIPK1	NOT	NOT	NOT	NOT	NOT	NOT	NOT	NOT	NOT
HOXA5	NOT	NOT	NOT	NOT	NOT	NOT	NOT	NOT	NOT
NRG2	NOT	NOT	NOT	NOT	NOT	NOT	NOT	NOT	NOT
LAMB3	NOT	NOT	NOT	NOT	NOT	NOT	NOT	UP	NOT
PTPRE	NOT	NOT	NOT	NOT	NOT	NOT	NOT	NOT	NOT
CACTIN	NOT	NOT	NOT	NOT	NOT	NOT	NOT	NOT	NOT
GRAMD1C	NOT	NOT	NOT	NOT	NOT	NOT	NOT	NOT	NOT
WFS1	NOT	NOT	NOT	NOT	NOT	NOT	NOT	NOT	NOT
ORAI2	NOT	NOT	NOT	NOT	NOT	NOT	NOT	NOT	NOT
CSRNP2	NOT	NOT	NOT	NOT	NOT	NOT	NOT	NOT	NOT
KCNJ14	NOT	NOT	NOT	NOT	NOT	NOT	NOT	NOT	NOT
CRTC3	NOT	NOT	NOT	NOT	NOT	NOT	NOT	NOT	NOT
IL20RB	NOT	NOT	NOT	NA	NA	NA	NA	NOT	NA
EMX1	NOT	NOT	NOT	NOT	NOT	NOT	NOT	NOT	NOT
GRM1	NOT	NOT	NOT	NOT	NOT	NOT	NOT	NOT	NOT
RNF24	NOT	NOT	NOT	NOT	NOT	NOT	NOT	NOT	NOT
PRTG	NOT	NOT	NOT	NA	NA	NA	NA	NOT	NA
TPRX1	NA	NA	NA	NA	NA	NA	NA	NOT	NA
MMP14	NOT	NOT	NOT	NOT	NOT	NOT	NOT	NOT	NOT
CRTC2	NOT	NOT	NOT	NA	NA	NA	NA	NOT	NA
RHOBTB1	NOT	NOT	NOT	NOT	NOT	NOT	NOT	NOT	NOT
APBA1	NOT	NOT	NOT	NOT	NOT	NOT	NOT	NA	NOT
PIKFYVE	NOT	NOT	NOT	NOT	NOT	NOT	NOT	NOT	NOT
PCDH7	NOT	NOT	NOT	NOT	NOT	NOT	NOT	NOT	UP
CDK9	NOT	NOT	NOT	NOT	NOT	NOT	NOT	NOT	NOT
MBD1	NOT	NOT	NOT	NOT	NOT	NOT	NOT	NOT	NOT
BSDC1	NOT	NOT	NOT	NOT	NOT	NOT	NOT	NOT	NOT
ABR	DOWN	NOT	NOT	NOT	NOT	NOT	NOT	NOT	NOT
TCHP	NOT	NOT	NOT	NA	NA	NA	NA	NOT	NA
DBNDD2	NA	NA	NA	NA	NA	NA	NA	NA	NA
POT1	UP	NOT	NOT	NOT	NOT	NOT	NOT	NOT	NOT
PIK3CD	NOT	NOT	NOT	NOT	NOT	NOT	NOT	NOT	NOT
GPR63	NOT	NOT	NOT	NOT	NOT	NOT	NOT	NOT	NOT
CPLX3	NOT	NOT	NOT	NOT	NOT	NOT	NOT	NOT	NOT
FBXO11	NOT	NOT	NOT	NOT	NOT	NOT	NOT	NOT	NOT
RBM24	NOT	NOT	NOT	NA	NA	NA	NA	DOWN	NA
SORBS1	NOT	NOT	NOT	NOT	NOT	NOT	NOT	DOWN	NOT
ATP6V0A2	NOT	NOT	NOT	NOT	NOT	NOT	NOT	NOT	NOT
MED8	NOT	NOT	NOT	NOT	NOT	NOT	NOT	NOT	NOT

PNRC1	NOT	NOT	NOT	NOT	NOT	NOT	NOT	NOT	NOT
NCR3	NOT	NOT	NOT	NOT	NOT	NOT	NOT	NOT	NOT
UAP1L1	NOT	NOT	NOT	NOT	NOT	NOT	NOT	NOT	NOT
AMPD2	NOT	NOT	NOT	NOT	NOT	NOT	NOT	NOT	NOT
CYB5RL	NOT	NOT	NOT	NA	NA	NA	NA	NOT	NA
ZSWIM6	NOT	NOT	NOT	NA	NA	NA	NA	NOT	NA
RNF8	NOT	NOT	NOT	NOT	NOT	NOT	NOT	NOT	NOT
DAG1	NOT	NOT	NOT	NOT	NOT	NOT	NOT	NOT	NOT
LAIR1	NOT	NOT	NOT	NOT	NOT	NOT	NOT	NOT	NOT
PRM1	NOT	NOT	NOT	NOT	NOT	NOT	NOT	NOT	NOT
WWC1	NOT	NOT	NOT	NOT	NOT	NOT	NOT	NOT	NOT
SPSB4	NOT	NOT	NOT	NA	NA	NA	NA	NOT	NA
TPT1	NOT	NOT	NOT	NOT	NOT	NOT	NOT	NOT	NOT
DISC1	NOT	NOT	NOT	NOT	NOT	NOT	NOT	NA	NOT
PYGO2	NOT	NOT	NOT	NA	NA	NA	NA	NOT	NA
CTAGE1	NOT	NOT	NOT	NOT	NOT	NOT	NOT	NOT	NOT
LPL	NOT	NOT	NOT	NOT	NOT	NOT	NOT	NOT	NOT
ZNF667	DOWN	DOWN	DOWN	NOT	NOT	NOT	NOT	NOT	NOT
C22orf46	NOT	NOT	NOT	NOT	NOT	NOT	NOT	NOT	NOT
THBD	NOT	NOT	NOT	NOT	NOT	NOT	NOT	NOT	DOWN
GCSAM	NOT	NOT	NOT	NA	NA	NA	NA	NOT	NA
EIF4G2	NOT	NOT	NOT	NOT	NOT	NOT	NOT	NOT	NOT
FAM71E2	NOT	NOT	NOT	NA	NA	NA	NA	NOT	NA
SAMD4B	NOT	NOT	NOT	NOT	NOT	NOT	NOT	NOT	NOT
FRMD5	NOT	NOT	NOT	NA	NA	NA	NA	NOT	NA
TNFSF9	DOWN	NOT	NOT	NOT	NOT	NOT	NOT	NOT	NOT
TSTD2	NOT	NOT	NOT	NA	NA	NA	NA	NA	NA
FLOT1	NOT	NOT	NOT	NOT	NOT	NOT	NOT	NOT	NOT
CRTAP	NOT	NOT	NOT	NOT	NOT	NOT	NOT	NOT	NOT
DDX6	NOT	NOT	NOT	NOT	NOT	NOT	NOT	NOT	NOT
STARD3NL	UP	NOT	NOT	NA	NA	NA	NA	NOT	NA
UBE2R2	NOT	NOT	NOT	NA	NA	NA	NA	NOT	NA
CSF1	NOT	NOT	NOT	NOT	NOT	NOT	NOT	NOT	NOT
CACHD1	DOWN	NOT	NOT	NA	NA	NA	NA	DOWN	NA
MBNL3	NOT	NOT	NOT	NOT	NOT	NOT	NOT	NOT	NOT
ENY2	NOT	NOT	NOT	NOT	NOT	NOT	NOT	NOT	NOT
IGBP1	NOT	NOT	NOT	NOT	NOT	NOT	NOT	NOT	NOT
HLA-DOB	NOT	NOT	NOT	NOT	UP	NOT	UP	NOT	NOT
PKNOX1	NOT	NOT	NOT	NOT	NOT	NOT	NOT	NOT	NOT
KSR2	NOT	NOT	NOT	NA	NA	NA	NA	NOT	NA
RABGAP1L	NOT	NOT	NOT	NOT	NOT	NOT	NOT	NOT	NOT
GJC1	UP	NOT	NOT	NOT	NOT	NOT	NOT	NOT	NOT
SMARCA2	NOT	NOT	NOT	NOT	NOT	NOT	NOT	NOT	NOT
TK2	NOT	NOT	NOT	NOT	NOT	NOT	NOT	NOT	NOT
PDGFA	NOT	NOT	NOT	NOT	NOT	UP	NOT	NOT	NOT

LMAN2L	NOT	NOT	NOT	NOT	NOT	UP	NOT	NOT	NOT
SOX5	NOT	NOT	NOT	NOT	NOT	NOT	NOT	DOWN	NOT
KCNC1	NOT	NOT	NOT	NOT	NOT	NOT	NOT	NOT	NOT
ABLIM2	DOWN	NOT	NOT	NA	NA	NA	NA	NOT	NA
ZMIZ1	NOT	NOT	NOT	NOT	NOT	NOT	NOT	NOT	NOT
HHIPL2	NOT	NOT	NOT	NOT	NOT	NOT	NOT	NOT	NOT
PITPNA	NOT	NOT	NOT	NOT	DOWN	NOT	DOWN	NOT	NOT
SLC43A2	NOT	NOT	NOT	NA	NA	NA	NA	NOT	NA
TEX261	NOT	NOT	NOT	NOT	NOT	NOT	NOT	NOT	NOT
THPO	NOT	NOT	NOT	NOT	NOT	NOT	NOT	NOT	NOT
PRR14L	NOT	NOT	NOT	NOT	NOT	NOT	NOT	NOT	NOT
SCP2	NOT	NOT	NOT	NOT	NOT	NOT	NOT	NOT	NOT
CCNL1	UP	NOT	UP	NOT	NOT	DOWN	NOT	NOT	DOWN
RFX3	NOT	NOT	NOT	NOT	NOT	NOT	NOT	NOT	NOT
ST6GAL1	NOT	NOT	NOT	NOT	NOT	UP	UP	NOT	UP
HNF1A	NOT	NOT	NOT	NOT	NOT	NOT	NOT	NOT	NOT
SLC17A9	NOT	NOT	NOT	NOT	NOT	NOT	NOT	NOT	NOT
BCL7B	NOT	NOT	NOT	NOT	NOT	NOT	NOT	NOT	NOT
CLOCK	NOT	NOT	NOT	NOT	NOT	NOT	NOT	NOT	NOT
NCAN	NOT	NOT	NOT	NOT	NOT	NOT	NOT	NOT	NOT
PPP6R3	NOT	NOT	NOT	NOT	NOT	NOT	NOT	NOT	NOT
TSPAN17	NOT	NOT	NOT	NA	NA	NA	NA	NOT	NA
SLC6A1	NOT	NOT	NOT	NOT	NOT	NOT	NOT	NOT	NOT
ALPK3	NOT	NOT	NOT	NOT	NOT	NOT	NOT	NOT	NOT
CALM1	NOT	NOT	NOT	NOT	NOT	NOT	NOT	NOT	NOT
FMNL3	UP	UP	UP	NA	NA	NA	NA	NOT	NA
CCR9	NOT	NOT	NOT	NOT	NOT	NOT	NOT	NOT	NOT
MPRIP	NOT	NOT	NOT	NOT	NOT	NOT	NOT	NOT	NOT
LRTM2	NOT	NOT	NOT	NA	NA	NA	NA	NOT	NA
MCTP1	NOT	NOT	NOT	NOT	NOT	NOT	NOT	NOT	NOT
ZFYVE1	NOT	NOT	NOT	NA	NA	NA	NA	NOT	NA
GLDN	NOT	NOT	NOT	NA	NA	NA	NA	NOT	NA
PAK3	NOT	NOT	NOT	NOT	NOT	NOT	NOT	NOT	NOT
FNDC5	NOT	NOT	NOT	NA	NA	NA	NA	NOT	NA
MAP4K5	NOT	NOT	NOT	NOT	NOT	NOT	NOT	NOT	NOT
ATP1B2	NOT	NOT	NOT	NOT	NOT	NOT	NOT	NOT	NOT
TNRC6C	NOT	NOT	NOT	NA	NA	NA	NA	NOT	NA
ANO2	NOT	NOT	NOT	NOT	NOT	NOT	NOT	NOT	NOT
WNT2B	NOT	UP	UP	NOT	NOT	NOT	NOT	NOT	NOT
NFAM1	NOT	NOT	NOT	NA	NA	NA	NA	NOT	NA
MAPKAPK2	NOT	NOT	NOT	NOT	NOT	NOT	NOT	NOT	NOT
DLG2	NOT	NOT	NOT	NOT	NOT	NOT	NOT	NOT	NOT
SMIM7	NOT	NOT	NOT	NOT	NOT	NOT	NOT	NOT	NOT
YTHDF3	NOT	NOT	NOT	NOT	NOT	NOT	NOT	NOT	NOT
ADAM10	NOT	NOT	NOT	NOT	NOT	NOT	NOT	NOT	NOT
SAMD8	NOT	NOT	NOT	NA	NA	NA	NA	NOT	NA

DCHS1	NOT	NOT	NOT	NOT	NOT	NOT	NOT	NOT	NOT
TNR	NOT	NOT	NOT	NOT	NOT	NOT	NOT	NOT	NOT
FAM178B	NOT	NOT	NOT	NA	NA	NA	NA	NOT	NA
PDE1C	NOT	NOT	NOT	NOT	NOT	NOT	NOT	NOT	NOT
STC1	NOT	NOT	NOT	NOT	NOT	NOT	NOT	NOT	NOT
TRPS1	DOWN	NOT	NOT	DOWN	DOWN	NOT	DOWN	DOWN	NOT
PEX16	NOT	NOT	NOT	NOT	NOT	NOT	NOT	NOT	NOT
ZNF25	NOT	NOT	NOT	NA	NA	NA	NA	NOT	NA
PEA15	NOT	NOT	NOT	NOT	NOT	NOT	NOT	NOT	NOT
TAF11	NOT	NOT	NOT	NOT	NOT	NOT	NOT	NOT	NOT
CYB5B	NOT	NOT	NOT	NOT	NOT	NOT	NOT	NOT	NOT
LTBP2	NOT	NOT	NOT	NOT	NOT	NOT	NOT	NOT	NOT
LYSMD1	NOT	NOT	NOT	NA	NA	NA	NA	NOT	NA
VKORC1	NOT	NOT	NOT	NOT	NOT	NOT	NOT	NOT	NOT
ZNF24	NOT	NOT	NOT	NOT	NOT	NOT	NOT	NOT	NOT
ARF4	NOT	NOT	NOT	NOT	NOT	NOT	NOT	NOT	NOT
ELMO2	NOT	NOT	NOT	NOT	NOT	NOT	NOT	NOT	NOT
TBC1D24	UP	NOT	NOT	NA	NA	NA	NA	NOT	NA
ZNF581	NOT	NOT	NOT	NA	NA	NA	NA	NA	NA
MLXIP	UP	NOT	NOT	NOT	NOT	NOT	NOT	NOT	NOT
GPR155	NOT	NOT	NOT	NA	NA	NA	NA	NOT	NA
ABCB10	NOT	NOT	NOT	NA	NA	NA	NA	NOT	NA
PRRT2	NOT	NOT	NOT	NA	NA	NA	NA	NOT	NA
DPYSL2	UP	NOT	NOT	NOT	NOT	NOT	NOT	NOT	NOT
ABHD2	NOT	NOT	NOT	NOT	NOT	NOT	NOT	NOT	NOT
TMEM184A	DOWN	NOT	DOWN	NA	NA	NA	NA	NOT	NA
LMX1B	NOT	NOT	NOT	NOT	NOT	NOT	NOT	NOT	NOT
RAP2A	NOT	NOT	NOT	NOT	NOT	NOT	NOT	NOT	NOT
TOX2	NOT	NOT	NOT	NA	NA	NA	NA	NA	NA
TXNRD3	NA	NA	NA	NA	NA	NA	NA	NA	NA
PEX6	NOT	NOT	NOT	NOT	NOT	NOT	NOT	NOT	NOT
UBR2	NOT	NOT	NOT	NOT	NOT	NOT	NOT	NOT	NOT
WDR3	NOT	NOT	NOT	NOT	NOT	NOT	NOT	NOT	NOT
GRPEL2	NOT	NOT	NOT	NA	NA	NA	NA	NOT	NA
MAP2	NOT	NOT	DOWN	NOT	NOT	NOT	NOT	NOT	NOT
DAGLA	NOT	NOT	NOT	NOT	NOT	NOT	NOT	NOT	NOT
ALS2CL	DOWN	NOT	NOT	NOT	NOT	NOT	NOT	NOT	NOT
IPO11	NOT	NOT	NOT	NA	NA	NA	NA	NA	NA
VIPR1	NOT	NOT	NOT	NOT	NOT	NOT	NOT	NOT	NOT
KLHDC3	NOT	NOT	NOT	NOT	NOT	NOT	NOT	NOT	NOT
RIMS3	NOT	NOT	NOT	NOT	NOT	NOT	NOT	NOT	NOT
IL6R	NOT	NOT	NOT	NOT	NOT	NOT	NOT	NOT	NOT
DNAJC5G	NOT	NOT	NOT	NA	NA	NA	NA	NOT	NA
EMC6	NOT	NOT	NOT	NOT	NOT	NOT	NOT	NOT	NOT
B3GNT9	NOT	NOT	NOT	NA	NA	NA	NA	NOT	NA
EFR3B	NOT	NOT	NOT	NOT	NOT	NOT	NOT	NOT	NOT

ETS1	NOT	NOT	NOT	NOT	NOT	NOT	NOT	NOT	NOT
NUP153	UP	NOT	NOT	NOT	NOT	NOT	NOT	NOT	NOT
HOXA11	NOT	NOT	NOT	NOT	NOT	NOT	NOT	NOT	NOT
SPOCK2	UP	NOT	NOT	NOT	NOT	NOT	NOT	NOT	NOT
KHK	NOT	NOT	NOT	NOT	NOT	NOT	NOT	NOT	NOT
HYDIN	NOT	NOT	NOT	NA	NA	NA	NA	NOT	NA
SAMD5	DOWN	NOT	DOWN	NA	NA	NA	NA	NOT	NA
DENND5A	NOT	NOT	NOT	NOT	NOT	NOT	NOT	NOT	NOT
STX5	NOT	NOT	NOT	NOT	NOT	NOT	NOT	NOT	NOT
C9orf62	NOT	NOT	NOT	NA	NA	NA	NA	NOT	NA
PTPN14	NOT	NOT	NOT	NOT	NOT	NOT	NOT	NOT	NOT
C1QTNF7	DOWN	DOWN	DOWN	NA	NA	NA	NA	DOWN	NA
NR6A1	NOT	NOT	NOT	NOT	NOT	NOT	NOT	NOT	NOT
SNX17	NOT	NOT	NOT	NOT	NOT	NOT	NOT	NOT	NOT
C3orf62	NOT	NOT	NOT	NA	NA	NA	NA	NA	NA
OGDH	NOT	NOT	NOT	NOT	NOT	NOT	NOT	NOT	NOT
EPHA5	NOT	NOT	NOT	NOT	NOT	NOT	NOT	NOT	NOT
CNNM1	NOT	NOT	NOT	NOT	NOT	NOT	NOT	NOT	NOT
CDYL2	UP	NOT	NOT	NA	NA	NA	NA	NOT	NA
TGM2	NOT	NOT	NOT	NOT	NOT	NOT	NOT	NOT	UP
COLQ	NOT	NOT	NOT	NOT	NOT	NOT	NOT	NOT	NOT
MINK1	NOT	NOT	NOT	NOT	NOT	NOT	NOT	NOT	NOT
NFATC4	NOT	NOT	NOT	NOT	NOT	NOT	NOT	NOT	NOT
SP6	DOWN	DOWN	DOWN	NA	NA	NA	NA	NOT	NA
PROM2	DOWN	NOT	NOT	NA	NA	NA	NA	NOT	NA
GPX1	NOT	NOT	NOT	NOT	NOT	UP	NOT	NOT	NOT
CEP170B	NOT	NOT	NOT	NOT	NOT	NOT	NOT	NOT	NOT
LRRC32	NOT	NOT	NOT	NOT	NOT	NOT	NOT	NOT	NOT
TRIM66	NOT	NOT	NOT	NOT	NOT	NOT	NOT	NOT	NOT
TINF2	NOT	NOT	NOT	NOT	NOT	NOT	NOT	NOT	NOT
NAT8L	NOT	NOT	NOT	NA	NA	NA	NA	NOT	NA
EIF4A2	NOT	NOT	NOT	NA	NA	NA	NA	NOT	NA
CARHSP1	NOT	NOT	NOT	NOT	NOT	NOT	NOT	NOT	NOT
STEAP3	NOT	NOT	NOT	NOT	NOT	UP	NOT	NOT	UP
NEO1	NOT	NOT	NOT	NOT	NOT	NOT	NOT	NOT	NOT
TRIM74	NA	NA	NA	NA	NA	NA	NA	NA	NA
HS3ST3B1	NOT	NOT	NOT	NOT	NOT	NOT	NOT	NOT	NOT
C7orf43	NA	NA	NA	NA	NA	NA	NA	NA	NA
TRIM67	NA	NA	NA	NA	NA	NA	NA	NOT	NA
IRGQ	NOT	NOT	NOT	NOT	NOT	NOT	NOT	NOT	NOT
SUSD2	DOWN	DOWN	DOWN	NA	NA	NA	NA	NOT	NA
DCLK3	NOT	NOT	NOT	NA	NA	NA	NA	NOT	NA
CCNG1	NOT	NOT	NOT	NOT	NOT	NOT	NOT	NOT	NOT
CLEC6A	NA	NA	NA	NA	NA	NA	NA	NOT	NA
PPME1	NOT	NOT	NOT	NOT	NOT	NOT	NOT	NOT	NOT
SH3PXD2A	NOT	NOT	NOT	NOT	NOT	NOT	NOT	NOT	NOT

GATAD2B	NOT	NOT	NOT	NA	NA	NA	NA	NOT	NA
ATP7B	NOT	NOT	NOT	NOT	NOT	NOT	NOT	NOT	NOT
EME1	NOT	NOT	NOT	NA	NA	NA	NA	NOT	NA
KIAA0513	DOWN	NOT	NOT	NOT	NOT	NOT	NOT	NOT	NOT

Table S8f. Expression profiles of predicted target genes of hsa-miR-574-3p in GEO datasets.

Target genes	GSE63514(CvsN)	GSE63514(HvsN)	GSE63514(HCvsN)	GSE9750(CvsN)	GSE7803(CvsN)	GSE7803(HvsN)	GSE7803(HCvsN)	GSE52903(CvsN)	GSE27678(CvsN)
BACE1	NOT	NOT	NOT	NOT	NOT	NOT	NOT	NOT	NOT
STRN3	UP	NOT	NOT	NOT	NOT	NOT	NOT	NOT	NOT
SNCB	NOT	NOT	NOT	NOT	NOT	NOT	NOT	NOT	NOT

Table S8g. Expression profiles of predicted target genes of hsa-miR-625-3p in GEO datasets.

Target genes	GSE63514(CvsN)	GSE63514(HvsN)	GSE63514(HCvsN)	GSE9750(CvsN)	GSE7803(CvsN)	GSE7803(HvsN)	GSE7803(HCvsN)	GSE52903(CvsN)	GSE27678(CvsN)
MAP2K6	NOT	NOT	NOT	NOT	NOT	NOT	NOT	NOT	UP
EGFLAM	NOT	NOT	NOT	NA	NA	NA	NA	NOT	NA
IRAK2	NOT	NOT	NOT	NA	NA	NA	NA	NOT	NA
ZFPM2	UP	NOT	NOT	NOT	NOT	NOT	NOT	DOWN	NOT
CAMK4	NOT	NOT	NOT	NOT	NOT	NOT	NOT	NOT	NOT
KIF13A	NOT	NOT	NOT	NOT	NOT	NOT	NOT	NOT	NOT
ALAS1	NOT	NOT	NOT	NOT	NOT	NOT	NOT	NOT	NOT
ZSCAN29	NOT	NOT	NOT	NA	NA	NA	NA	NOT	NA
EXOC8	NOT	NOT	NOT	NA	NA	NA	NA	NOT	NA
DNAJA1	NOT	NOT	NOT	NOT	NOT	NOT	NOT	NOT	NOT
PAWR	NOT	NOT	NOT	NOT	NOT	NOT	NOT	NOT	NOT
GALNT1	NOT	NOT	NOT	NOT	NOT	NOT	NOT	NOT	NOT
PYGO1	NOT	NOT	NOT	NOT	NOT	NOT	NOT	NOT	NOT
RBM12B	UP	NOT	UP	NOT	NOT	NOT	NOT	NOT	NOT
USP9X	NOT	NOT	NOT	NOT	NOT	NOT	NOT	NOT	NOT
CPEB4	NOT	NOT	NOT	NA	NA	NA	NA	NOT	NA
RBM27	NOT	NOT	NOT	NA	NA	NA	NA	NOT	NA
GSE1	NOT	NOT	NOT	NOT	NOT	NOT	NOT	NOT	NOT
ZC3H6	NOT	NOT	NOT	NA	NA	NA	NA	NOT	NA
PRPF4	UP	NOT	NOT	NOT	NOT	NOT	NOT	NOT	NOT
HMGB3	UP	NOT	UP	UP	UP	NOT	UP	NOT	UP
ABHD17B	NOT	NOT	NOT	NOT	NOT	NOT	NOT	NOT	NOT
CYP3A7	NA	NA	NA	NA	NA	NA	NA	NA	NA
THRAP3	NOT	NOT	NOT	NOT	NOT	NOT	NOT	NOT	NOT
ZBTB44	NOT	NOT	NOT	NOT	NOT	NOT	NOT	NOT	NOT
DNAJA2	NOT	NOT	NOT	NOT	NOT	NOT	NOT	NOT	NOT
RUNDC3B	NOT	NOT	NOT	NOT	NOT	NOT	NOT	NOT	NOT
ZC3H12B	NOT	NOT	NOT	NA	NA	NA	NA	NOT	NA
WNK3	UP	UP	UP	NA	NA	NA	NA	NOT	NA
PPP6C	NOT	NOT	NOT	NOT	NOT	NOT	NOT	NOT	NOT
TGFBR1	NOT	NOT	NOT	NOT	NOT	DOWN	NOT	NOT	NOT
LHX8	NOT	NOT	NOT	NA	NA	NA	NA	NOT	NA

CNTN4	NOT	NOT	NOT	NA	NA	NA	NA	DOWN	NA
LEPROTL1	UP	NOT	NOT	NOT	NOT	NOT	NOT	NOT	NOT
FLG2	NOT	NOT	NOT	NA	NA	NA	NA	NOT	NA
OSBPL8	NOT	NOT	NOT	NOT	NOT	NOT	NOT	NOT	NOT
TWISTNB	NOT	NOT	NOT	NOT	NOT	NOT	NOT	NOT	NOT
RBFOX2	NOT	NOT	NOT	NOT	NOT	NOT	NOT	NOT	NOT
ATF7IP	NOT	NOT	NOT	NOT	NOT	NOT	NOT	NOT	NOT
PIP5K1B	NOT	NOT	NOT	NOT	NOT	NOT	NOT	NOT	UP
FAM151B	NA	NA	NA	NA	NA	NA	NA	NOT	NA
LGR5	NOT	NOT	NOT	NOT	NOT	NOT	NOT	NOT	NOT
GABRA4	NOT	NOT	NOT	NOT	NOT	NOT	NOT	NOT	NOT
NEUROD1	NOT	NOT	NOT	NOT	NOT	NOT	NOT	NOT	NOT
FAM19A1	NA	NA	NA	NA	NA	NA	NA	NA	NA
MAD2L1	UP	UP	UP	UP	UP	NOT	UP	UP	UP
STRN	NOT	NOT	NOT	NOT	NOT	NOT	NOT	NOT	NOT
COL4A3BP	NA	NA	NA	NA	NA	NA	NA	NA	NA
ECT2L	NA	NA	NA	NA	NA	NA	NA	NA	NA
TJP1	NOT	NOT	NOT	NOT	NOT	NOT	NOT	NOT	NOT
SLC39A10	UP	NOT	NOT	NA	NA	NA	NA	NOT	NA
NDUFAF5	NOT	NOT	NOT	NOT	NOT	NOT	NOT	NOT	NOT
ZBTB2	UP	NOT	NOT	NA	NA	NA	NA	NOT	NA
WDR76	UP	UP	UP	UP	NOT	NOT	NOT	UP	NOT
HOOK3	NOT	NOT	NOT	NA	NA	NA	NA	NOT	NA
ZSWIM6	NOT	NOT	NOT	NA	NA	NA	NA	NOT	NA
ASIC5	NOT	NOT	NOT	NA	NA	NA	NA	NOT	NA
GGPS1	NOT	NOT	NOT	NOT	NOT	NOT	NOT	NOT	NOT
SMC6	UP	NOT	UP	NOT	NOT	NOT	NOT	NOT	NOT
ATRNL1	NOT	NOT	NOT	NOT	NOT	NOT	NOT	NOT	NOT
B3GNT5	UP	NOT	UP	NA	NA	NA	NA	NOT	NA
FOXN2	UP	NOT	NOT	NOT	NOT	NOT	NOT	NOT	NOT
HOXA7	NOT	NOT	NOT	NOT	NOT	NOT	NOT	NOT	NOT
HIF1A	NOT	NOT	NOT	NOT	NOT	NOT	NOT	NOT	NOT
IAPP	NOT	NOT	NOT	NOT	NOT	NOT	NOT	NOT	NOT
SRGAP1	UP	NOT	NOT	NA	NA	NA	NA	NOT	NA
STARD4	UP	UP	UP	NA	NA	NA	NA	NOT	NA
SOX11	NOT	NOT	NOT	NOT	NOT	NOT	NOT	NOT	NOT
TNFSF11	NOT	NOT	NOT	NOT	NOT	NOT	NOT	NOT	NOT
ANKRD20A3	NA	NA	NA	NA	NA	NA	NA	NA	NA
ATG3	NOT	NOT	NOT	NOT	NOT	NOT	NOT	NOT	NOT
ESCO1	NOT	NOT	NOT	NA	NA	NA	NA	NOT	NA
TMEFF2	DOWN	NOT	NOT	NA	NA	NA	NA	NOT	NA
DESI2	UP	NOT	NOT	NOT	NOT	NOT	NOT	NOT	NOT
ATRN	NOT	NOT	NOT	NOT	NOT	NOT	NOT	NOT	NOT

ELL2	NOT	NOT	NOT	DOWN	DOWN	DOWN	DOWN	NOT	DOWN
DGKH	NOT	NOT	NOT	NOT	NOT	NOT	NOT	NOT	NOT
KLHL4	NOT	NOT	NOT	NOT	NOT	NOT	NOT	NOT	NOT
FMNL2	NOT	NOT	NOT	NA	NA	NA	NA	NOT	NA
ANKRD20A4	NA	NA	NA	NA	NA	NA	NA	NA	NA
ETV1	UP	NOT	NOT	NOT	NOT	NOT	NOT	NOT	NOT
RBM17	NOT	NOT	NOT	NOT	NOT	NOT	NOT	NOT	NOT
DPY19L2	NOT	NOT	NOT	NA	NA	NA	NA	NOT	NA
FREM2	NOT	NOT	NOT	NA	NA	NA	NA	NOT	NA
SMIM15	NOT	NOT	NOT	NA	NA	NA	NA	NOT	NA
MXI1	NOT	NOT	NOT	NOT	NOT	NOT	NOT	NOT	NOT

Table S9. Most likely target genes selected by differential analysis.

miRNAs	Most likely target genes
**hsa-miR-26b-5p**	*ZNF462, ST6GAL2, TOB1, FRMD4B, RBM24, RBM20, HPGD, MSMO1, EPHA2, RPS6KA6, POLR3G, ESR1, MAP2, PIM1, CPM, ALDH5A1, SLC19A2, ZFHX4, ZCCHC24, KLF4, ADAMTS19, ADM, FAM160A1, ZNF710, SOSTDC1, CPED1, RANBP9, DUSP5, NIPAL2, CCND2, PGRMC2, PCDH18, WNK1, SLC16A6, WNT5A, EPHA7, ARL4C, CPEB2, ZNF516, C18orf25, LTBP1*
**hsa-miR-146b-5p**	*NOVA1, SORT1*
**hsa-miR-191-5p**	*SATB1, WIZ*
**hsa-miR-484**	*TRPS1 , SORBS2 , CACHD1 , C1QTNF7 , DACH1 , PTGER4*
**hsa-miR-574-3p**	___
**hsa-miR-625-3p**	*TMEFF2 , ELL2 , TGFBR1 , CNTN4*
